# NDR kinase SAX-1 controls dendrite branch-specific elimination during neuronal remodeling in *C. elegans*

**DOI:** 10.1038/s44318-026-00841-w

**Published:** 2026-06-27

**Authors:** Paola V Figueroa-Delgado, Shaul Yogev

**Affiliations:** 1 Department of Cell Biology, Yale School of Medicine, New Haven, CT USA; 2 Department of Neuroscience, Yale School of Medicine, New Haven, CT USA

**Keywords:** Development, Membranes & Trafficking, Neuroscience

## Abstract

Neuronal remodeling is crucial for proper nervous system development and function. Despite significant advances, the underlying mechanisms that govern this process remain poorly understood. Here, we adapted *C. elegans* IL2 sensory neurons as a model system to study developmental and organismal stress-associated dendrite remodeling. Upon entering developmental diapause, IL2 dendrites grow a complex dendritic arbor, which is later pruned when reproductive development resumes. We identified unexpected specificity in the pruning process, with distinct genetic requirements to direct branch-specific elimination of secondary, tertiary, and quaternary branches. The serine/threonine kinase SAX-1/NDR promotes elimination of secondary and tertiary, but not quaternary, dendrites. SAX-1 functions with its conserved interactors SAX-2/Furry and MOB-2 in the removal of both dendritic branches. The guanine-nucleotide exchange factor RABI-1/Rabin8 and the small GTPase RAB-11.2 mediate the elimination of secondary branches with SAX-1, but their effect on tertiary branches is minimal. Consistent with the known roles of RABI-1 and RAB-11.2 in regulating membrane dynamics, we find that SAX-1 promotes endocytosis during remodeling. Together, our findings reveal distinct mechanisms for dendrite branch-specific elimination during neuronal remodeling.

## Synopsis

The mechanisms governing neuronal remodeling during development and in response to stress remain poorly understood. Using *C. elegans* IL2 sensory neurons as a model for developmental and organismal stress-induced remodeling, this study shows that the conserved SAX-1/NDR kinase directs selective IL2 dendrite pruning via distinct genetic pathways.

- SAX-1/NDR and its conserved interactors SAX-2/Furry and MOB-2 are required for the elimination of IL2Q secondary and tertiary dendrites.
- RABI-1/Rabin8 and RAB-11.2 primarily mediate secondary dendrite elimination, potentially via RABI-1/Rabin8-mediated activation of RAB-8 and RAB-10.
- Loss of *sax-1/NDR* results in a retention of SAX-2/Furry puncta and a decrease in endocytic events following induction of dendrite pruning.

## Abstract (web summary)

Dendritic pruning during *C. elegans* development shows unexpected specificity in genetic requirements for elimination of secondary, tertiary, and quaternary branches.

## Graphical abstract

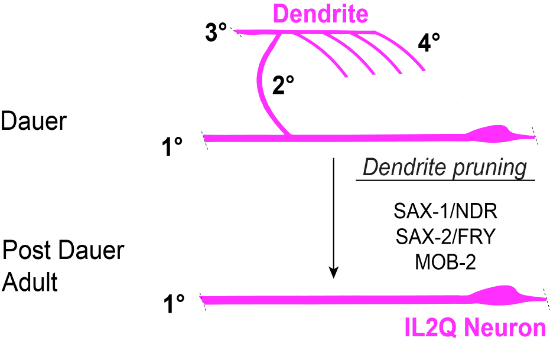

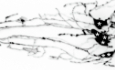

## Introduction

Neuronal remodeling is essential for proper nervous system development and function. Dendrite remodeling can be initiated by stereotyped developmental programs, activity-dependent mechanisms or stress (Brunson et al, 2005; Furusawa and Emoto, 2021; McEwen et al, 2016; Riccomagno and Kolodkin, 2015; Schuldiner and Yaron, 2015). For example, in *Drosophila*, dendrite pruning is regulated by developmental ecdysone signaling in the mushroom body and in the peripheral sensory neurons during morphogenesis (Kuo et al, 2005; Lee et al, 2000). In contrast, in mammalian hippocampal neurons, chronic stress has been shown to promote dendrite retraction (Christian et al, 2011; Magariños et al, 1996; McEwen et al, 2016; Vyas et al, 2002). Although deregulated neuronal remodeling has been suggested to be involved in neurodevelopmental and neuropsychiatric disorders such as Autism Spectrum Disorder and Schizophrenia (Riccomagno and Kolodkin, 2015), the mechanisms that govern this process remain poorly understood.

A myriad of cell biological mechanisms cooperate to direct dendrite pruning, including protease activation, transport, cytoskeletal dynamics, and membrane dynamics (Furusawa and Emoto, 2021; Krämer et al, 2019; Kuo et al, 2006; Lin et al, 2015; Riccomagno and Kolodkin, 2015; Rui, 2024; Rumpf et al, 2019; Schuldiner and Yaron, 2015; Williams and Truman, 2005). Evidence for the role of membrane dynamics comes primarily from *Drosophila* class IV da neurons, where localized endocytic events at proximal dendrites correlate with membrane thinning and precede pruning of the dendritic arbor (Kanamori et al, 2015, 2013; Zhang et al, 2014). In the same system, the recycling endosome protein Rab11 is required for dendrite pruning, at least partially through the removal of the cell surface protein Neuroglian (Krämer et al, 2019; Lin et al, 2020; Zhang et al, 2014). Rab11 can bind the guanine-nucleotide exchange factor (GEF) Rabin8, which can activate Rab8 and Rab10 (Feng et al, 2015; Homma and Fukuda, 2016; Knödler et al, 2010; Westlake et al, 2011). Although Rab11 has been implicated in membrane retrieval and removal of surface Neuroglian, whether Rabin8, and its associated GTPases, function in dendrite pruning has not been investigated.

Nuclear Dbf2-related (NDR) kinases are AGC family serine/threonine kinases that are evolutionarily conserved from yeast to humans (Hergovich et al, 2006; Santos et al, 2023; Tamaskovic et al, 2003). NDR kinases regulate cell shape, growth, and polarity (Chen et al, 2019; Geng et al, 2000; Hergovich et al, 2006; Verde et al, 1998; Zallen et al, 2000). Mutations in NDR kinases lead to ectopic membrane growth in *C. elegans* and mammalian neurons (Gallegos and Bargmann, 2004; Roşianu et al, 2023; Zallen et al, 2000), and to excessive dendrite branching in *Drosophila*, consistent with a role in restricting cellular growth (Emoto et al, 2004). The mechanism by which NDR kinases restrict cellular growth remains unclear, although several NDR substrates, including Rabin8, regulate membrane dynamics (Roşianu et al, 2023; Ultanir et al, 2012). Whether NDR kinases, in addition to restricting neurite growth, promote neurite elimination is unknown.

Here, we establish *C. elegans* inner labia 2 dorsal and ventral (IL2Q) neurons as a genetically tractable model for dendrite pruning. IL2Q primary dendrites elaborate new branches when *C. elegans* enters an organismal stress-induced and developmentally encoded quiescence-state known as dauer arrest (Androwski et al, 2017; Schroeder et al, 2013). These newly generated branches are then eliminated upon return to favorable conditions (Schroeder et al, 2013). We find that IL2Q pruning shows branch-specific and context-specific genetic requirements: the NDR kinase SAX-1 is required for pruning secondary and tertiary, but not quaternary branches. SAX-1 promotes branch elimination in post dauer larvae induced by the *daf-7/*TGF-β or *daf-2/*Insulin-receptor pathways, but not by starvation. SAX-1 functions with its conserved interactors SAX-2/Furry and MOB-2 to regulate membrane dynamics during IL2Q pruning. SAX-1 functions with the guanine-nucleotide exchange factor RABI-1/Rabin8 and the small GTPase RAB-11.2, which are primarily required for secondary dendrite elimination. These results provide insights into cell-biological mechanisms that underpin remodeling of complex dendritic arbors during development and in response to changes in environmental conditions.

## Results

### *shy87* mutants disrupt dendrite remodeling

**Figure 1:**
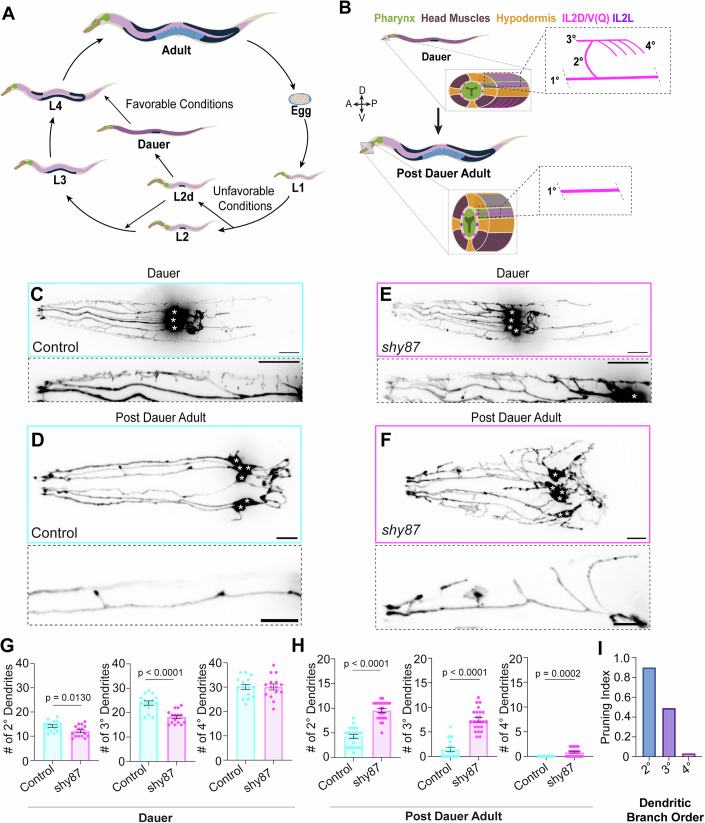
*shy87* mutants exhibit a remodeling defect in post dauer adults. (**A**) Schematic depicting *C. elegans* life cycle. Adapted from WormAtlas. Under unfavorable conditions, the nematode arrests into an alternative developmental molt (dauer). Upon re-exposure to favorable conditions, reproductive development is resumed into adulthood. (**B**) Schematic of IL2 dorsal and ventral (Q) (magenta) neuron morphology in *C*. elegans dauer and post dauer adult. Oblique transverse schematic of IL2Q neurons at dauer arrest (top) and in a post dauer adult worm (bottom). Pharynx (green); Head muscles (purple); Hypodermis (yellow). At dauer, the IL2Q neurons extend a stereotypical dendritic arbor characterized by a 2° dendrite extending from a 1° dendrite towards the dorsal midline; 3° dendrites bifurcate from 2° dendrite towards the anterior and posterior in parallel to 1° dendrite; 4° dendrites extending into the head muscle quadrants (dark purple). When reproductive development is resumed, post dauer adult worms remove the higher-order dendrites, leaving the 1° dendrite intact. (**C**, **D**) Z-projection of control dauer (**C**) and post dauer adult (**D**) expressing *tba-6*p::tagRFP in IL2s (top). Zoomed inset of select IL2 dorsal neuron (bottom, dashed box). Asterisks indicate IL2 cell bodies. (**E**, **F**) Maximum intensity Z-projection of *shy87* mutant dauer (**E**) and post dauer adult (**F**) expressing cytosolic tagRFP under *tba-6*p (top). Zoomed inset of select IL2 dorsal neuron (bottom, dashed box). Scale bar, 10μm. Asterisks indicate IL2 cell bodies. (**G**) Quantification of the number of IL2Q 2°-4° dendrites at dauer comparing *shy87* mutants (*n* = 15) to control animals (*n* = 15). Unpaired *t* test. Error bars represent ± SEM. (**H**) Quantification of the number of IL2Q higher-order dendrites in control (*n* = 20) versus *shy87* mutants post dauer adults (*n* = 24). Unpaired *t* test with Welch’s correction or one sample *t* and Wilcoxon test (post dauer adult 4°). Error bars represent ± SEM. (**I**) Pruning index calculated as: 1 –  [(*shy87*_(dauer median – post dauer adult median)_/(control _(dauer median – post dauer adult median)_)]. Values were calculated separately for secondary, tertiary, and quaternary dendrites. Values approaching 0 indicate pruning comparable to control; values approaching 1 indicate an absolute failure to prune. The pruning index reveals deficits in 2°and 3° dendrites in *shy87* mutants. Source data are available online for this figure.

**Figure EV1:**
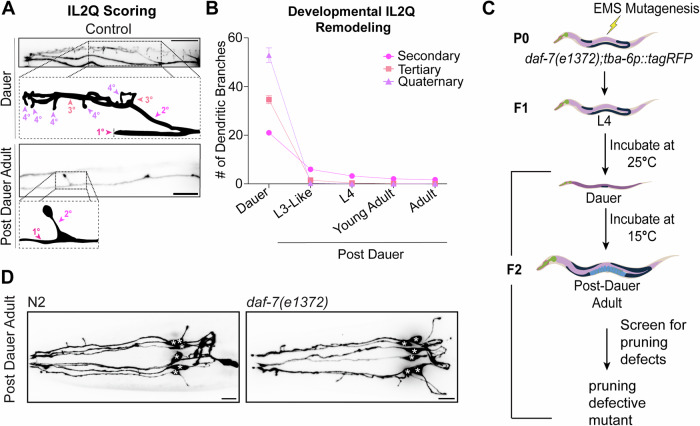
Experimental paradigm for IL2Q neuronal remodeling and scoring strategy. (**A**) IL2Q scoring: Representative Z-projection insets of select IL2Q neurons in control animals at dauer and post dauer adults, with schematic overlays indicating (with arrowhead) branch order (1°–4°) used for scoring dendrites. Control insets were intentionally reused from Fig. 1C,D to demonstrate the scoring approach used for quantification. Scale bar, 10 μm. (**B**) Line graph depicting the progressive elimination of IL2Q higher-order dendrites following dauer recovery towards adulthood, *n* = 11-53. (**C**) Schematic depicting strategy for the genetic screen. (**D**) Representative maximum intensity Z-projections of post dauer adults in N2 (left) and *daf-7(e1372)* mutants (right) show no observable difference in post dauer adults IL2Q morphology or pruning. Asterisks represent IL2 cell bodies. Scale bar, 10 μm. Source data are available online for this figure.

To study dendrite remodeling, we adapted the Inner Labial 2 (IL2) sensory neurons of *C. elegans* as an experimental model. IL2s extend an anterior primary dendrite that terminates in a sensory cilium at the tip of the nose, and project short axons that run posteriorly to the nerve ring, where they turn circumferentially (White et al, 1986). The IL2 dendrite is unbranched in well-fed animals undergoing reproductive development. However, two IL2 pairs (IL2D and IL2V, referred to as IL2Q) undergo extensive and stereotypic branching during dauer arrest—an alternative developmental diapause that is induced by organismal stress conditions such as overpopulation or starvation (Fig. 1A,B). In dauer, IL2Q primary (1°) dendrites extend secondary (2°), tertiary (3°), and quaternary (4°) branches at roughly 90° to each other, forming a characteristic dendrite arbor (Figs. 1C and EV1A). Upon re-exposure to favorable conditions, IL2Q eliminate most of their dauer-generated dendritic branches (Figs. 1B,D and EV1A,B), leaving primary dendrites intact (Schroeder et al, 2013). The factors that control IL2Q dendrite elimination following dauer recovery are unknown.

To uncover new regulators of neuronal remodeling, we first optimized conditions for an unbiased visual genetic screen for IL2Q remodeling-defective mutants. IL2 neurons were visualized by expressing a cytosolic TagRFP driven by the *tba-6* promoter, an α-tubulin isoform that is enriched in IL2s (Hurd et al, 2010; Schroeder et al, 2013; Nishida et al, 2021). We synchronized dauer entry and exit using a temperature-sensitive allele of the TGF-β homolog *daf-7(e1372)*, which causes constitutive dauer entry at 25 °C and allows recovery from dauer and the resumption of reproductive development at 15 °C (Ren et al, 1996; Riddle et al, 1981). IL2Q morphology at dauer and in post dauer adults was qualitatively similar in *daf-7(e1372)* and wild-type N2 animals (Fig. EV1D). Hence, unless otherwise noted, we use *daf-7(e1372)* as a control strain.

**Figure EV2:**
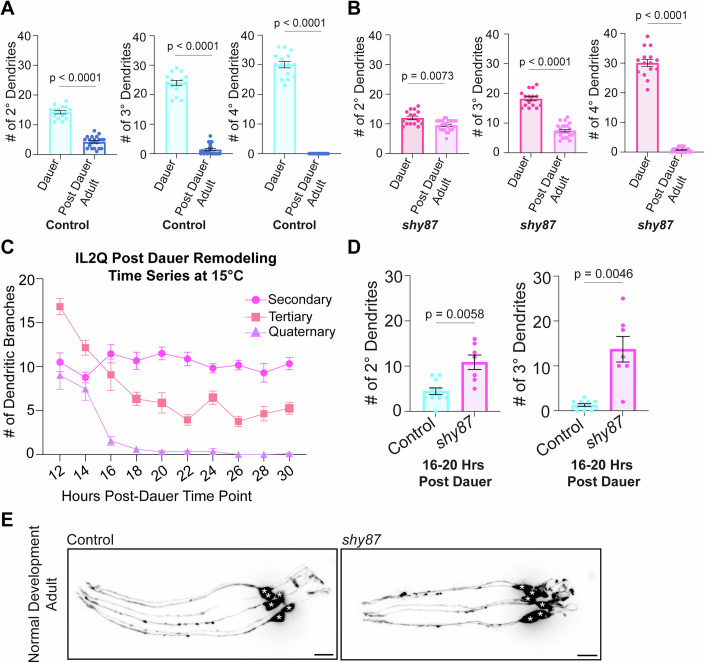
*sax-1* is required for branch elimination, not inhibiting branch growth. (**A**) Internal comparison of the total number of neurites in control dauer (*n* = 15) versus control post dauer adults (*n* = 20), whereas there is a significant reduction in the number of IL2Q in post dauer adults when compared to dauer. Suggesting successful elimination of the IL2Q dendritic arbor post-dauer. Statistical significance was determined using a Mann–Whitney test for control 3° and 4° dendrites and Welch’s *t* test for 2° dendrites. Statistical significance was determined using a Mann–Whitney test for control 3° and 4° dendrites and Welch’s *t* test for 2° dendrites. Error bars represent ±SEM. (**B**) Internal comparison of the total number of IL2Q neurites in *shy87* mutant dauers (*n* = 15) versus post dauer adults (*n* = 24). The number of 2° dendrites in post dauer adult *shy87* mutants remained comparable to that of *shy87* mutant dauers; meanwhile, 3° dendrites slightly decreased in number and 4° dendrites were entirely eliminated in post dauer adults. Suggesting a maintenance of the dendritic arbor, specifically 2° dendrites and a subset of 3° dendrites post dauer. Statistical significance was determined using a Mann–Whitney test for control 2° and 3° dendrites and Welch’s *t* test for 3° dendrites. Error bars represent ±SEM. (**C**) Line graph depicting the elimination of IL2Q higher-order dendrites over time (hours) post dauer (transfer to 15°C) in control animals, *n *= 6–17. Error bars represent ±SEM. (**D**) Quantification of total 2° and 3° dendrite numbers in control (*n* = 11) and *shy87* mutants (*n* = 7) at 16–20 h post dauer. Unpaired *t* test with Welch’s correction. Error bars represent ±SEM. (**E**) Representative confocal images of adult control and *shy87* mutant animals under normal reproductive development. Asterisks indicate IL2 cell bodies. Scale bar, 10 μm. Source data are available online for this figure.

From a screen of roughly 3000 haploid genomes (screen strategy outlined in Fig. EV1C), we isolated a mutant, *shy87*, that exhibited a striking maintenance of IL2Q dendrite branches in post dauer adults. *shy87* mutants eliminated 4° dendritic branches nearly as efficiently as controls, but showed excessive maintenance of 2° and 3° dendritic branches (Fig. 1C–I, EV2A,B), suggesting that *shy87* is required for the elimination of secondary and tertiary dendritic branches. To test whether this defect reflects a failure to eliminate dauer-generated branches or an earlier developmental defect, we quantified the total number of IL2Q dendrites across 2°-4° branch orders in control and *shy87* mutants at dauer (Fig. 1G). *shy87* mutant dauers showed a minor reduction in secondary and tertiary branches compared to control (Fig. 1G). These results suggest that *shy87* is predominantly required for the elimination of dauer-generated dendrite branches, although earlier roles cannot be ruled out.

### The conserved serine/threonine kinase SAX-1/NDR is required for dendrite branch-specific elimination

**Figure 2:**
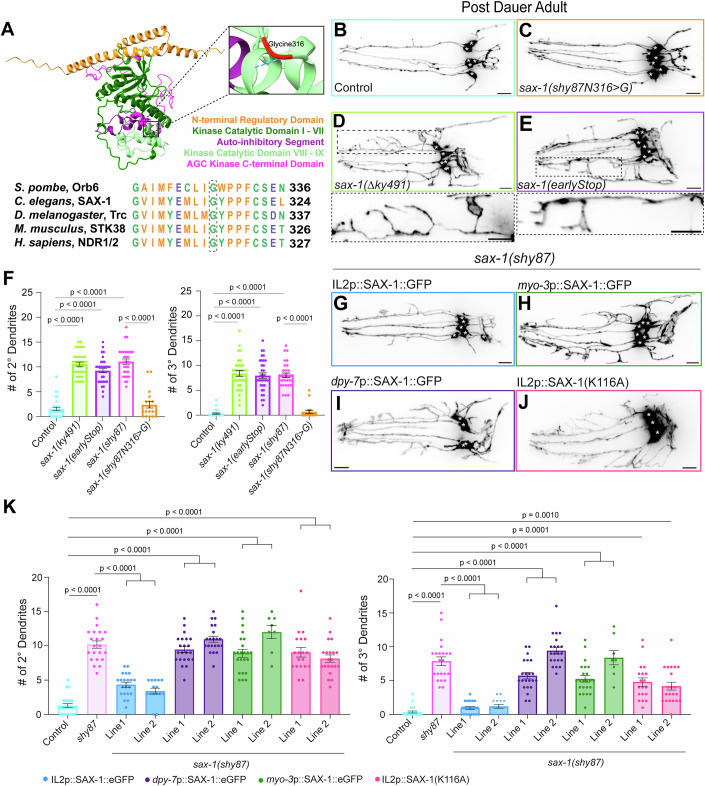
SAX-1/NDR promotes IL2Q remodeling in a cell-specific and kinase activity-dependent manner. (**A**) AlphaFold predicted structure of SAX-1 with key functional domains indicated: N-terminal regulatory domain (1–79 AA, orange), kinase catalytic domain I-VII (80–270 AA, dark green), auto-inhibitory sequence (270–310 AA, dark purple), kinase catalytic domain VIII-IX (310–370 AA, light green), and the AGC family kinase C-terminal domain (370–446 AA, magenta). Zoomed view of Glycine316 (red), which is conserved across species from yeast to human homologs of NDR1/2 kinases and mutated into an asparagine in *shy87* mutants. (**B**–**E**) Maximum intensity Z-projection of control (**B**), engineered repair of the *shy87* allele (**C**), *ky491* deletion (**D**), and *early stop* (**E**) post dauer adult animals, demonstrating that loss of *sax-1* function is responsible for the pruning defects in *shy87* mutants. Zoomed inset of select IL2Q are shown for *ky491* deletion (**D**) and *early stop* (**E**) with a dashed box. Scale bars, 10 μm. Asterisks indicate IL2 somas. (**F**) Quantifications of IL2Q 2° and 3° dendrite number in post dauer adults of control (*n* = 34) and *sax-1* alleles, including *ky491* (*n* = 32), *earlyStop* (*n* = 34), and *shy87N316* > *G* (*n* = 18). Brown–Forsythe and Welch ANOVA with Dunnett’s correction (2° dendrites) or Kruskal–Wallis test with Dunn’s correction (3° dendrites), all genotypes compared to control (*n* = 34) and *shy87* mutants (*n* = 30). All *sax-1* mutant alleles phenocopied *shy87*. Repair of the *shy87* missense point mutation rescued the mutant phenotype back to control numbers. (**G**–**J**) Maximum intensity Z-projection of *shy87* post dauer adult mutants expressing wild-type SAX-1 cDNA fused to GFP under tissue-specific promoters in IL2 neurons (*tba-*6p; **G**), muscle (*myo-3*p; **H**), and hypodermis (*dpy-*7p; **I**). (**J**) SAX-1 kinase-catalytic dead/dominant negative construct (K116A) was expressed under an IL2-specific promoter (*tba-6*p). Scale bars, 10 μm. Asterisks indicate IL2 cell bodies. (**K**) Quantification of the total number of IL2Q 2° and 3°dendrites in post dauer adults from control animals (*n* = 25), *sax-1* mutants (*n* = 22), IL2-specific transgenic lines expressing SAX-1::GFP (*n* = 15–21), muscle-specific transgenic lines expressing SAX-1::GFP (*n* = 8–23), hypodermis-specific transgenes expressing SAX-1::GFP (*n* = 21–22), or SAX-1 kinase-catalytic dead lines (*n* = 13–14). Kruskal–Wallis with Dunn’s correction, with comparisons made to control animals. Error bars represent ± SEM. Source data are available online for this figure.

**Figure EV3:**
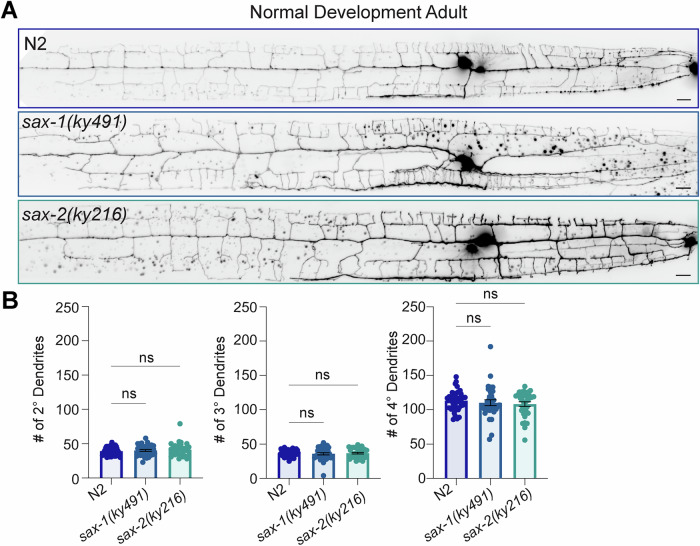
SAX-1 and SAX-2 are not required for PVD neuron branching. (**A**) Representative images of polymodal PVD neurons in N2 (*n* = 40), *sax-1* (*n *= 33), and *sax-2* (*n* = 33) mutant animals. Scale Bar, 10 µm. (**B**) Quantifications of total number of PVD dendrite orders (2°–4°). Brown–Forsythe and Welch ANOVA with Dunnett’s multiple comparison. Error bars are ±SEM. Source data are available online for this figure.

We next used whole-genome sequencing and SNP mapping to identify a signature Ethyl Methanesulfonate (EMS) mutation (G > A) that converts a glycine to an asparagine at position 316 of the conserved serine/threonine kinase SAX-1 in *shy87* mutants (Fig. 2A). We validated *sax-1(shy87)* as the causal mutation for pruning defects with three complementary approaches. First, we tested a partial deletion allele, *sax-1(ky491)*, and found that it phenocopied *shy87* (Fig. 2D,F). Second, we generated an early stop codon in *sax-1* in control animals using CRISPR-Cas9, which led to pruning defects indistinguishable from *shy87* (Fig. 2E,F). Lastly, we reverted the mutated asparagine 316 back to a glycine in *shy87* mutants and found that this rescued the mutant phenotype (Fig. 2C,F) comparable to control (Fig. 2B). Together, these results indicate that *shy87* is a mutation in *sax-1*, and that *sax-1* is required for IL2Q dendrite remodeling. To test whether *sax-1* has a broader requirement in dendrite morphogenesis, we tested if its loss affected dendrite elaboration in the highly arborized sensory neuron PVD. Mutations in *sax-1* or its interactor *sax-2* (discussed ahead) did not affect PVD arbor development (Fig. EV3), consistent with recent work that examined the role of *sax-1* in PVD neurons (Zhao et al, 2022).

NDR kinases are required to restrict cell size across organisms (Emoto et al, 2004; Gallegos and Bargmann, 2004; Hergovich et al, 2006; Roşianu et al, 2023; Tamaskovic et al, 2003; Tay et al, 2019). In *C. elegans* and mice, NDR mutants show excessive growth of neuronal membranes, and in *Drosophila*, mutations in the NDR homolog Tricornered lead to excessive dendrite branching and loss of self-avoidance (Emoto et al, 2004; Gallegos and Bargmann, 2004; Roşianu et al, 2023; Zallen et al, 2000). It is therefore unexpected that in IL2Q *sax-1* is required to eliminate existing processes rather than to restrict growth. To confirm that dendrite branch maintenance in *sax-1* mutants is not due to ectopic regrowth after initial elimination, we compared the total number of IL2Q branches between dauer and post dauer adults in control and *sax-1(shy87)* mutants (Fig. EV2A,B). Control post dauer adults exhibited a significant reduction when compared to control dauers (Fig. EV2A), consistent with the elimination of the dendritic arbor. In contrast, *shy87* mutants largely maintained their dendritic arbors, with 2° branches mostly preserved and a subset of 3° branches only mildly reduced (Fig. EV2B). Next, we systematically characterized the time course of dendrite remodeling following recovery from dauer arrest. In control animals, 4° dendrites were mostly eliminated by 16 h. Most 3° branches were eliminated between 16 and 22 h (Fig. EV2C), whereas 2° branches were eliminated closer to the L4 molt. Examination of *sax-1* mutants at 16–20 h, when most 3° branches undergo elimination, revealed that these branches are maintained in the mutants (Fig. EV2D). This result indicates that excessive dendrite branches in *sax-1* mutant adults reflect a failure to eliminate dauer-born branches rather than ectopic branch regrowth.

**Figure EV4:**
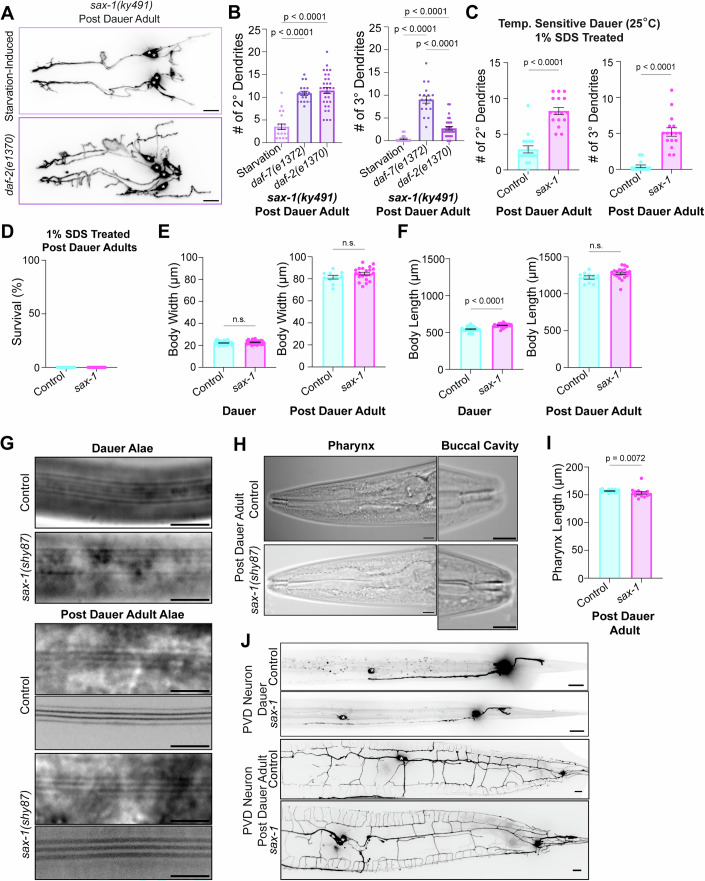
Validation of dauer exit in *sax-1* mutants. (**A**) Z-projection of *sax-1(ky491)* post dauer adults following dauer induction via starvation (top) or a dauer constitutive mutant, *daf-2/Insulin-receptor* (bottom). Scale bar, 10 μm. (**B**) Quantification of 2° and 3° dendrites of *sax-1(ky491)* mutants following dauer induction via *daf-7(e1372)* (*n* = 18), *daf-2(e1370)* (*n* = 31), and starvation conditions (*n* = 21). Brown–Forsythe and Welch ANOVA with Dunnett’s correction. Error bars are ±SEM, with individual data points shown. (**C**) Quantification of 2° and 3° dendrites in control (*n* = 17) and *sax-1* mutant (*n* = 16) post dauer adults. Dauers were treated with 1% SDS for true dauer selection and exclusion of partial dauers. Mann–Whitney U test. Error bars represent ±SEM. (**D**) Quantification of the percentage of post dauer adults that survived 1% SDS treatment. (**E**, **F**) Quantifications of body width (μm) and length (μm) in control (*n* = 33 and *sax-1* mutant (*n *= 28) animals at dauer and post dauer adults. Statistical significance was determined using Welch’s *t* test or unpaired *t* test. Error bars represent ±SEM. (**G**) Micrographs of dauer and post dauer adult alae for control and *sax-1* mutants. Scale bar, 10 μm. (**H**) Micrographs of post dauer adult control and *sax-1* mutant pharynx and buccal cavity (zoomed inset). Scale bar, 10 μm. (**I**) Quantification of pharynx length of control (*n* = 16) versus *sax-1* mutants (*n* = 15). Mann–Whitney *U* test. Error bars represent ±SEM. Post dauer adults exhibit normal pharyngeal and buccal cavity morphology, suggesting no persistent morphological differences from dauer arrest. (**J**) Representative micrographs of the multi-dendritic neuron PVD (mid to posterior dendrite) in dauer and post dauer adults of control and *sax-1* mutants. Scale bar, 10 μm. PVD remains unbranched at dauer and branches normally after resuming reproductive development, suggesting no stark post dauer branching defects. Source data are available online for this figure.

Dauer entry involves drastic changes to animal physiology (Burnell et al, 2005; Cassada and Russell, 1975; Gerisch et al, 2001; Golden and Riddle, 1984). Furthermore, the method of dauer induction can affect the characteristics of the dauer larvae (Golden and Riddle, 1982; Hu, 2007; Karp, 2018). For example, the transcriptomes of *daf-7* and *daf-2* mutant dauers are more similar to each other than to heat-induced N2 dauers. Both mutants can also induce dauer formation in dauer-defective *(Daf-d)* mutants, whereas starvation cannot (Corchado et al, 2026; Karp, 2018). We therefore asked whether the requirement for *sax-1* in controlling IL2Q dendrite morphology depends on the selected method of dauer induction. First, we confirmed that passage through dauer was required for the dendritic phenotypes in *sax-1* mutants (Fig. EV2E). Next, we tested whether the requirement for *sax-1* is specific to dauers induced by the temperature-sensitive *daf-7*/TGF-β mutation. For this, we examined IL2Q morphology in *sax-1* mutants following dauer arrest induced by starvation or by using a mutant allele of the insulin receptor *daf-2(e1370)*, which promotes dauer entry through a different pathway than *daf-7(e1372)* (Gottlieb and Ruvkun, 1994; Hu, 2007; Karp, 2018; Ren et al, 1996; Riddle et al, 1981). Surprisingly, starved animals did not require *sax-1* for dendrite remodeling, whereas in *daf-2* mutants, similar to *daf-7* mutants, required *sax-1* for dendrite remodeling (Fig. EV4A,B). One possible interpretation of these results is that *sax-1* mutants impair dauer exit in *daf-7* and *daf-2* mutants, leading to maintenance of some dauer characteristics such as IL2Q branches. However, careful examination of dauer and post-dauer hallmarks (e.g. SDS-sensitivity, body size, buccal plug, alae and pharynx morphology) in control and *daf-7;sax-1* double-mutants did not reveal apparent differences (Fig. EV4C–I). To test a neuron-specific marker for dauer exit, we examined PVD—a sensory neuron in which dendrite elaboration is arrested during dauer and resumed after dauer exit (Richardson et al, 2019). *daf-7; sax-1* double mutants had a fully elaborated PVD arbor in post dauer adults (Fig. EV4J), indicative of successful dauer exit. We conclude that *sax-1* does not interfere with dauer exit in *daf-7* mutants. Therefore, it is likely that the differential requirement for *sax-1* in IL2Q dendrite pruning reflects a difference between N2 dauers and *daf-2* or *daf-7* dauers.

### SAX-1/NDR acts cell-autonomously and is kinase activity dependent

To determine whether SAX-1 functions cell autonomously in IL2Q to promote dendrite pruning, we expressed *sax-1* cDNA fused to GFP with the IL2-specific *tba-6* promoter in *shy87* mutants (Fig. 2G). SAX-1::GFP localization was cytosolic, as previously reported (Zallen et al, 2000) and its expression in IL2 led to a robust decrease in the number of IL2Q 2° and 3° dendrites (Fig. 2K). Ectopic expression of SAX-1 in the hypodermis and the muscle, driven by the *dpy-7* and *myo-*3 promoters, respectively, failed to rescue the post dauer *shy87* mutant phenotype (Fig. 2H,I,K). These data suggest that *sax-1* functions cell-autonomously in IL2Q to mediate dendrite pruning.

To test whether SAX-1 function depends on its kinase activity, we expressed SAX-1 cDNA with a mutation in a conserved lysine (K116A) that is required for catalytic activity (Carrera et al, 1993; Millward et al, 1998; Ultanir et al, 2012) in *shy87* mutants. SAX-1(K116A) did not rescue the branching defects of *sax-1* mutants across multiple transgenic lines tested (Fig. 2J,K). Together, these data indicate that SAX-1/NDR functions cell-autonomously in IL2Q in a kinase-activity-dependent manner.

### MOB-2 and SAX-2/Furry function with SAX-1/NDR to direct IL2Q dendrite elimination

**Figure 3:**
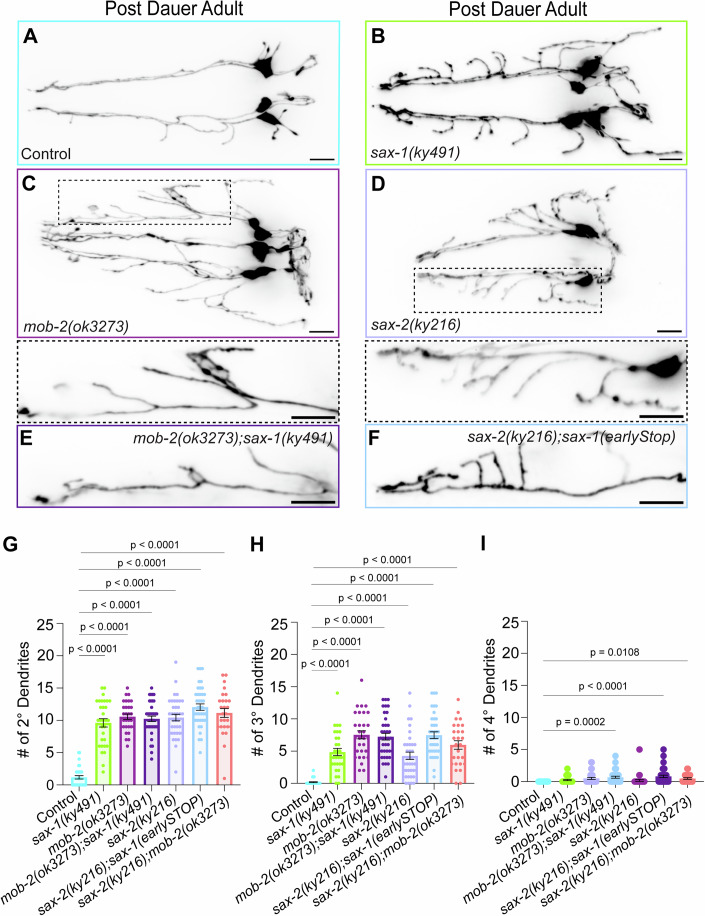
MOB-2 and SAX-2/Furry function with SAX-1/NDR to direct IL2Q dendrite elimination. Post dauer adult confocal images of (**A**) control (*n* = 32) and (**B**) *sax-1* loss-of-function (*n* = 31), (**C**) *mob-2* (*n* = 30), (**D**) *sax-1;mob-2* double mutants (*n* = 38), (**E**) *sax-2* (*n* = 37), (**F**) *sax-1;sax-2* double-mutants (*n* = 39), and *mob-2;sax-2* double mutants (*n* = 27). Zoomed inset of select IL2Q are shown for *mob-*2 and *sax-2* single mutants with a dashed box. Scale bars, 10 μm. (**G**–**I**) Quantification of the total number of 2° (**G**), 3° (**H**), and 4° (**I**) dendrites in post dauer adults for genotypes shown in (**A**–**F**). Biological replicates consisted of individual worms. Each point represents the total number of indicated IL2Q branches in a single animal. Kruskal–Wallis test with Dunn’s correction. Error bars are ±SEM, with individual data points shown. Source data are available online for this figure.

To identify proteins that function with SAX-1 in IL2Q dendrite elimination, we conducted a candidate genetic screen (Table 1). From this screen, we found that the conserved NDR activator MOB-2 and the binding partner SAX-2/Furry were required for the elimination of IL2Q branches. Similar to *sax-1* (Fig. 3B)—and in contrast to control (Fig. 3A)—*mob-2* (Fig. 3C) and *sax-2* (Fig. 3D) mutants exhibited retention of secondary and tertiary branches in adults, while quaternary branches were largely eliminated (Fig. 3G–I). Mps*1*-binder-related (MOB) proteins bind the N-terminal Regulatory (NTR) region of NDR kinases and promote their activation (Bichsel et al, 2004; Devroe et al, 2004; Gógl et al, 2015; Hergovich, 2011). SAX-2/Furry is a ~ 300 kD protein containing Armadillo repeats that may act as a scaffold for SAX-1/NDR (Chiba et al, 2009; Cong et al, 2001; Nagai and Mizuno, 2014) and functions with NDR kinases in neurite growth and dendrite tiling (Zallen et al, 2000; Emoto et al, 2004; Gallegos and Bargmann, 2004). To assess if *sax-1*, *mob-2*, and *sax-2* function in the same genetic pathway, we generated *sax-1;mob-*2, *sax-1;sax*-2, and *mob-2;sax-2* double-mutants and compared their phenotypes to those of single mutants and controls (Fig. 3A,E–I). Double-mutants did not show an additive effect, suggesting that they act within the same genetic pathway. These results suggest that a conserved SAX-1/SAX-2/MOB-2 complex is required for IL2Q dendrite pruning.

**Table 1:** Candidate genetic screen for SAX-1/NDR kinase interactors and substrates.

Gene	Allele	Mammalian ortholog	Phenotype	References
**Cell growth and size regulators**				
*sax-2*	*ky216*	Furry	IL2Q pruning defect post dauer	Cervino et al, 2021; Fang et al, 2010; He et al, 2005; Nagai and Mizuno, 2014; Roşianu et al, 2023
*F09A5.4/mob-2*	*ok3273*	Mob2	IL2Q pruning defect post dauer	Devroe et al, 2004
*yap-1*	*tm1416*	YAP1	No phenotype observed	Irie et al, 2020; Zhang et al, 2015
*rict-1*	*mg360*	Rictor	No phenotype observed	Koike-Kumagai et al, 2009; Wu et al, 2013
*unc-82*	*e1323*	NUAK1	No phenotype observed	Suzuki et al, 2006
**Actin Regulators**				
*unc-43*	*e408*	CaMKII	No phenotype observed	Rehberg et al, 2014; Zallen et al, 2000
*wsp-1*	*gm324*	WASP	No phenotype observed	Natarajan et al, 2015
**Endocytosis and membrane trafficking**				
*sel-5*	*ok363*	Aak1	No phenotype observed	Roşianu et al, 2023; Ultanir et al, 2012
*pifk-1*	*tm2348*	PI4KB	No phenotype observed	Roşianu et al, 2023
*mig-10*	*ok2499*	Raph1	No phenotype observed	Roşianu et al, 2023
*F58G6.1/amph-1*	*tm1060*	BIN1	No phenotype observed	Roşianu et al, 2023
*F54C9.11/rabi-1*	*tm2518*	Rabin8	IL2Q pruning defect post dauer	Chiba et al, 2013; Deretic et al, 2023; Fresquez et al, 2025; Ultanir et al, 2012
*rab-11.1*	*tm2251*	Rab11A	No phenotype observed	Burguete et al, 2024; Chiba et al, 2013; Roşianu et al, 2023
*rab-11.2*	*tm2081*	Rab11A	IL2Q pruning defect post dauer	Burguete et al, 2024; Chiba et al, 2013; Roşianu et al, 2023
*rab-10*	*ok1494*	Rab10	IL2Q branching defect at dauer	Chiba et al, 2013; Fresquez et al, 2025; Homma and Fukuda, 2016
*rab-8*	*tm2991*	Rab8B	ILQ branching defect at dauer and post dauer	Chiba et al, 2013; Fresquez et al, 2025; Homma and Fukuda, 2016
**Mitochondrial quality control**				
*pink-1*	*ok3538*	PINK1	No phenotype observed	Wu et al, 2013

### SAX-2/Furry localization depends on SAX-1

**Figure 4:**
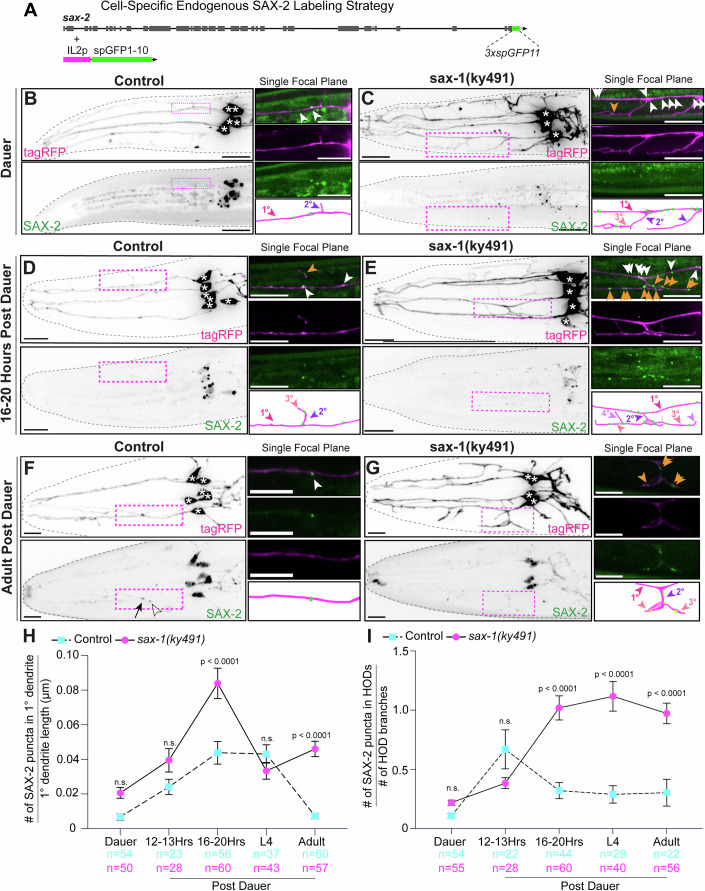
SAX-2/Furry localization depends on SAX-1 kinase. (**A**) Schematic illustrating the strategy to endogenously label SAX-2 in IL2 neurons using split-GFP, with GFP1-10 expressed under IL2 specific promoter. (**B**, **C**) Representative images of endogenous SAX-2 (pseudo-colored green) in IL2 neurons (tagRFP, pseudo-colored magenta) at dauer arrest in control (**B**) and *sax-1* mutants (**C**). Magenta boxes indicate the regions shown in the single focal plane insets. Zoomed insets highlight the primary (1°) dendrite. White arrowheads indicate SAX-2 puncta along the 1° dendrite; orange arrowheads indicate puncta in higher-order dendrites. Cartoon schematics indicate the regions shown in the magnified insets. Asterisks denote cell bodies. Scale bar, 10 μm. (**D**, **E**) Representative images of IL2Q neurons 16–20 h post dauer in control (**D**) and *sax-1* mutants (**E**). Insets show single IL2Q 1° dendrites. Arrowheads as in (**B**, **C**). Scale bar, 10 μm. (**F**, **G**) Representative images of endogenous SAX-2 puncta in IL2Q neurons in post dauer adults in control (**F**) and *sax-1* (**G**) mutants. In control, the white arrowhead points to a typical SAX-2 puncta, meanwhile, the black arrow points at SAX-2 puncta that overlap with cytosolic accumulations. Scale bar, 10 μm. Asterisks represent IL2 cell bodies. (**H**) Quantification of the total number of SAX-2 puncta in 1° dendrites normalized by the total length of the 1° dendrite for dauer and multiple post dauer time points. (**I**) Quantification of total number of endogenous SAX-2 puncta in higher order dendrites normalized to the total number of higher order branches for dauer and multiple post dauer time points. Control and *sax-1* mutant sample sizes for each timepoint are reported in each graph and are representative of individual neurons measured (*n*), to capture neuron-to-neuron variability. Two-way ANOVA with Tukey’s multiple comparison test. Error bars are ±SEM. Source data are available online for this figure.

To gain insights into the functions of SAX-1 and SAX-2 in dendrite remodeling, we inserted three copies of spGFP11 (3xspGFP11) at the endogenous C-terminus of SAX-2 with CRISPR-Cas9 and visualized it in IL2Q by expressing spGFP1-10 under the *tba-6* promoter (Fig. 4A). Tagging of SAX-2 with spGFP11 did not lead to dendrite remodeling defects, suggesting that the tag is functional (Fig. 4F). In control animals, SAX-2 was concentrated in the cell body in punctate structures, reminiscent of its localization in other neurons and tissues (Gallegos and Bargmann, 2004; Park et al, 2024). Within dendrites, SAX-2 signal was generally faint, with puncta rarely appearing along the primary dendrite or at varicosities and higher-order branches (Fig. 4B,D,F). Interestingly, 12–13 h after dauer exit, dendritic SAX-2 levels increased, suggesting that SAX-2 may act locally within the dendrite during IL2Q remodeling (Fig. 4D,F,H,I). In *sax-1* mutants, we observed a robust increase of dendritic SAX-2 puncta (Fig. 4C,E,G–I), predominantly along secondary and tertiary branches at multiple time points. We quantified the abundance of SAX-2 separately for primary and higher order dendrites, since primary dendrites are preserved, whereas higher-order dendrites are largely eliminated in control animals 16–20 h post dauer (Fig. 4H,I). These results indicate that *sax-1* is required for the dendritic localization of SAX-2. The increase in SAX-2 puncta in *sax-1* mutants is consistent with a scenario where SAX-2 is turned over during normal pruning, such that its persistence reflects disrupted pruning in the absence of SAX-1. Nonetheless, additional models cannot be excluded (see “Discussion”).

### SAX-1 coordinates IL2Q remodeling with RABI-1/Rabin8 and RAB-11.2

**Figure 5:**
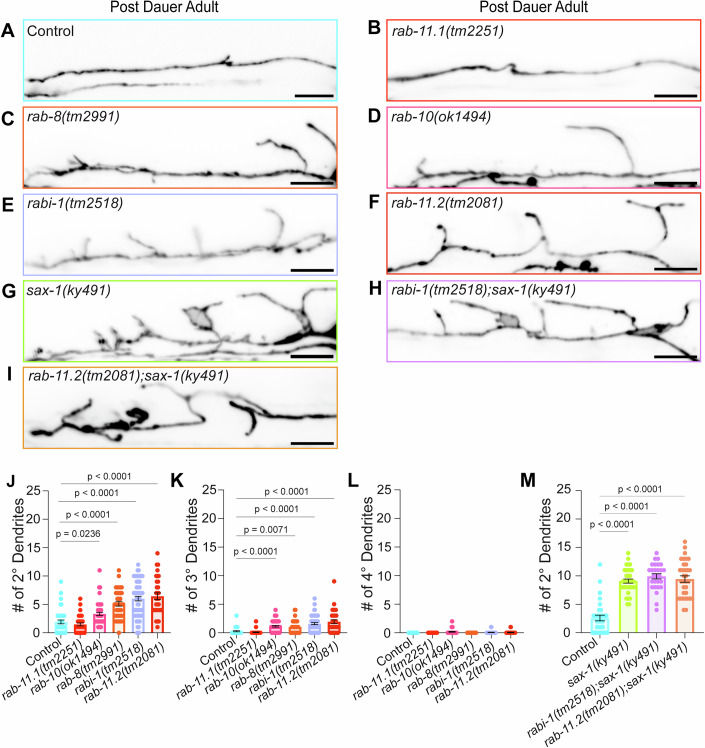
SAX-1 coordinates IL2Q remodeling with RABI-1/Rabin8 and RAB-11.2. (**A**–**I**) Representative confocal images of select IL2Q insets for (**A**) control (*n* = 41/32), (**B**) *rab-11.1* (*n* = 33), (**C**) *rab-8* (*n* = 37), (**D**) *rab-10* (*n* = 58), (**E**) *rabi-1* (*n* = 62), (**F**) *rab-11.2* (*n* = 32), (**G**) *sax-1* (*n* = 35), (**H**) *rabi-1;sax-1* (*n* = 27), and (**I**) *rab-11.2*;*sax-1* (*n* = 33) post dauer adult mutants. Scale bar, 10 μm. Error bars represent ±SEM. (**J**–**L**) Quantification of total number of 2° (**J**), 3° (**K**), and 4° (**L**) dendrites for (**A**–**F**). Biological replicates consisted of individual worms. Each point represents the total number of indicated IL2Q branches in a single animal. Kruskal–Wallis with Dunn’s correction. Error bars represent ±SEM. (**M**) Quantification of total number of 2° dendrites in indicated genotypes. Each data point represents the total number of 2° dendrites in a single animal. Kruskal–Wallis with Dunn’s correction. Error bars are ±SEM, with individual data points shown. Source data are available online for this figure.

Since SAX-1/NDR kinases are emerging as key regulators of membrane dynamics (Fresquez et al, 2025; Roşianu et al, 2023), we tested whether specific Rab GTPases involved in membrane recycling, along with other regulators of membrane dynamics, act as effectors of SAX-1/NDR1/2-mediated dendrite remodeling (Table 1). We found that mutants of RABI-1/Rabin8, a Rab8 guanine-nucleotide exchange factor (GEF), that was previously identified as a direct phosphorylation target of NDR kinases (Ultanir et al, 2012; Chiba et al, 2013; Roşianu et al, 2023), failed to eliminate their 2° dendrites post dauer (Fig. 5E,J). Tertiary dendrites were also affected (Fig. 5K), but the phenotype was very subtle when compared to *sax-1, sax-2* or *mob-2* mutants, while 4° were efficiently eliminated (Fig. 5L). Double-mutants of *sax-1* and *rabi-1* did not show an additive effect and phenocopied the severity of *sax-1* single mutants (Fig. 5G,H,M). These results suggest that *rabi-1* and *sax-1* function in the same genetic pathway that coordinates secondary dendrite elimination, while *sax-1* likely directs elimination of tertiary dendrites with additional downstream regulators.

Rabin8 functions as a GEF for Rab8 and Rab10, promoting their activation during membrane trafficking (Feng et al, 2012; Hattula et al, 2002; Homma and Fukuda, 2016; Knödler et al, 2010). We found that *rab-8* and *rab-10* mutants showed mild pruning defects of secondary dendrites, reminiscent to *rabi-1* mutants (Fig. 5C,D,J,K). These results suggest that RABI-1/Rabin8 likely functions with RAB-8 and RAB-10 during dendrite pruning.

Rabin8 recruitment to recycling endosomes depends on its interaction with GTP-bound Rab11 (Chiba et al, 2013; Feng et al, 2015; Homma and Fukuda, 2016; Fresquez et al, 2025). To test the involvement of Rab11 in IL2Q dendrite remodeling post dauer, we tested mutant alleles of two closely related Rab11 homologues, *rab-11.1* and *rab-11.2*. Similar to control (Fig. 5A), *rab-11.1* mutants showed grossly normal IL2Q morphology during dauer and had no pruning defect following dauer exit (Fig. 5B,J–L). In contrast, *rab-11.2* mutants showed a pruning defect following dauer exit that was similar to *rabi-1* mutants (Fig. 5F,J–L). In addition, *rab-11.2;sax-1* double mutants did not enhance the *sax-1* mutant phenotype (Fig. 5I,M), suggesting that they function in the same genetic pathway that eliminates secondary dendrites. Together, these results suggest that RABI-1/Rabin8 and RAB-11.2 function with SAX-1/NDR kinase to eliminate secondary dendrites, likely by coordinating membrane dynamics during IL2Q pruning.

### SAX-1 coordinates membrane retrieval

**Figure 6:**
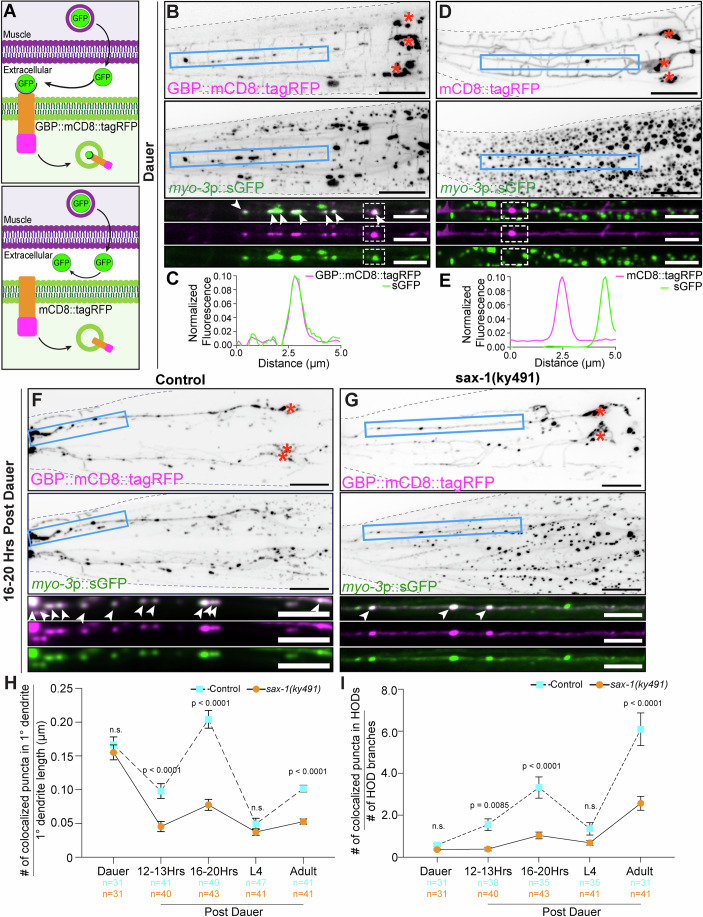
*SAX-1/NDR* promotes endocytic events during pruning. (**A**) (Top) Schematic of generic endocytosis reporter. mCD8::tagRFP (magenta) fused to a GFP-binding nanobody are expressed under an IL2-specific promoter. Secreted GFP (green) from muscle binds the nanobody, enabling visualization of endocytosed GFP-mCD8 complexes as magenta/green colocalized puncta. (Bottom) Schematic of a control endocytosis reporter chimera lacking the GFP-binding nanobody. (**B**–**E**) Representative confocal images of IL2Q neurons at dauer expressing either the control endocytosis reporter (**B**) or a construct lacking the GFP-binding nanobody (**D**). Blue boxes indicate regions shown in the single focal plane insets. Zoomed insets show a single IL2Q 1° dendrite. White arrowheads indicate colocalized puncta along 1° dendrites. Asterisks mark cell bodies. Scale bars, 10 μm. (**C**) Normalized fluorescence linescan of endocytic reporter showing co-localization of a select magenta-green puncta (white dashed box) at dauer. (**E**) Normalized fluorescence linescan of construct lacking GFP-binding nanobody showing lack of co-localization of magenta-green puncta (white dashed box) at dauer. (**F**, **G**) Representative images of endocytosis reporter in IL2Q neurons at 16–20 h post dauer in control (**F**) and *sax-1* mutants (**G**). Blue boxes indicate regions shown in single focal plane insets. Zoomed insets show a single IL2Q 1° dendrite. White arrowheads depict magenta-green colocalized puncta. Asterisks (orange) indicate IL2 cell bodies. Scale bar, 10μm. GFP (green) puncta outside the neuron represent either extracellular (secreted) or muscle-associated (non-secreted) GFP. Due to select Z-projection of the representative images, some puncta may appear absent; these structures are visible in the complete Z-stack. (**H**) Quantification of the total number of colocalized magenta-green puncta in 1° dendrites normalized by 1° dendrite length for control and *sax-1* mutants at multiple time points. (**I**) Quantification of the total number of colocalized puncta in higher order dendrites normalized to the total number of higher order branches for dauer and multiple post dauer timepoints. Control and *sax-1* mutant sample sizes for each timepoint are reported in each graph and are representative of individual neurons measured (*n*). Sample sizes are reported in each graph. Two-way ANOVA with Tukey’s multiple comparison test. Error bars represent ±SEM. Source data are available online for this figure.

The involvement of *rab-11.2* in *sax-1-*mediated dendrite remodeling suggests that SAX-1 may function to coordinate membrane retrieval during branch elimination. To visualize endocytosis during IL2Q remodeling, we used an established genetically encoded reporter (Richardson et al, 2019). We expressed secreted GFP harboring a signal peptide from muscle and expressed in IL2 neurons a chimera consisting of RFP and the transmembrane domain of mCD8 fused to an anti-GFP nanobody (GBP) (Fig. 6A). GFP binding to GBP leads to GFP accumulating on the surface of IL2Q, and their endocytosis appears as GFP and RFP colocalized puncta (Fig. 6B,C) (Richardson et al, 2019). We used a chimera lacking GBP as a control and found that this prevented the recruitment of muscle-secreted GFP to IL2Q surface or endocytic puncta (Fig. 6D,E).

We observed that the number of colocalized puncta increased in control IL2Q around the peak of pruning (16–20 h following dauer recovery) and in post dauer adults (Fig. 6F,H,I). In contrast, *sax-1* mutants showed no significant increase following dauer exit, and the number of colocalized puncta remained consistently lower than in control animals across multiple timepoints (Fig. 6G–I). These results suggest that *sax-1* is required for the formation or stabilization of endocytic sites during IL2Q dendrite pruning following dauer recovery.

## Discussion

The mechanisms that govern neuronal remodeling during development and in response to external conditions remain poorly understood. Here, by adapting *C. elegans* IL2Q dendrites as a model for developmentally encoded and organismal stress-related dendrite remodeling, we identified a novel role for the conserved SAX-1/NDR kinase in dendrite branch-specific elimination. Our results indicate that SAX-1/NDR and its conserved interactors SAX-2/Furry and MOB-2 are required for the elimination of IL2Q secondary and tertiary dendrites. SAX-1/NDR is required for dendrite pruning following recovery from a developmental diapause induced by manipulating *daf-7/TGF-β* or *daf-2/Insulin-receptor* signaling, but is dispensable when diapause is induced by starvation. Additionally, we find that SAX-1 functions with the guanine-nucleotide exchange factor RABI-1/Rabin8 and the small GTPase RAB-11.2 to direct the elimination of 2° dendrite branches in post dauer animals. RABI-1/Rabin8 may activate small GTPases RAB-8 and RAB-10 to mediate 2° dendrite elimination and, consequently, regulate membrane retrieval. The known involvement of these proteins in membrane trafficking (Chiba et al, 2013; Fresquez et al, 2025; Homma and Fukuda, 2016; Ultanir et al, 2012; Westlake et al, 2011) and our results with a genetically encoded endocytosis reporter suggest that SAX-1 promotes dendrite elimination by regulating membrane dynamics. Together, these results reveal unexpected state- and branch-specific dendrite elimination mechanisms during neuronal remodeling, governed by a conserved regulator of polarized cell growth.

SAX-1/NDR kinases are conserved regulators of polarized cell growth (Chen et al, 2019; Das et al, 2009; Gupta and McCollum, 2011; Tamaskovic et al, 2003; Verde et al, 1998). Loss of NDR activity leads to increased cell growth in fission yeast and mammals (Das et al, 2009; Demiray et al, 2018; Hergovich et al, 2006). In *C. elegans* and *Drosophila* neurons, mutants in *sax-1* or its homolog *Trc*, respectively, fail to terminate axon growth or display excessive dendrite branching (Gallegos and Bargmann 2004; Emoto et al, 2004; Chung et al, 2016). More recently, it was shown that in hippocampal CA1 neurons, combined loss of NDR1 and NDR2 results in ectopic membrane protrusions (Roşianu et al, 2023), a phenotype that is notably similar to that of *sax-2* mutants in *C. elegans* (Park et al, 2024). Together, these results suggest a general role in restricting cell size. However, the role we identified for *sax-1* and *sax-2* in IL2Q is not in restricting cellular growth, as *sax-1* and *sax-2* mutants did not exhibit overgrowth of IL2Q branches at dauer arrest. Instead, following the temporal progression of IL2Q pruning, we found that the aberrant dendritic branches observed in mutant adults are not ectopic outgrowths, but rather the result of failed dendrite elimination. Therefore, NDR kinases are able to both inhibit growth and promote the elimination of cellular processes. How these two functions are coordinated remains to be determined. NDR kinases likely engage distinct substrates in a context-dependent manner, enabling it to differentially regulate cellular outcomes. Alternatively, since the elaboration of cellular processes such as dendritic branches involves coordinated growth and retraction (Shi et al, 2026; Smith et al, 2010), it is possible that some of the excessive branching observed in NDR kinase mutants reflects a failure in branch retraction.

How does SAX-1 promote 2° and 3° dendrite pruning? Our findings support that SAX-1 acts as a kinase and functions cell autonomously with its conserved interactors SAX-2/Furry and MOB-2. NDR kinases are activated through binding to MOB family proteins, which enhance NDR phosphorylation and stabilize their active conformation (Bichsel et al, 2004; Hergovich et al, 2005), while Furry proteins may serve as scaffolds to support kinase function (Chiba et al, 2009; Nagai and Mizuno, 2014). NDR kinases can direct growth by regulating the cytoskeleton (Das et al, 2009; Norkett et al, 2020) and membrane dynamics, including regulation of exocytosis, endocytosis, membrane asymmetry, intracellular trafficking, and autophagy (Joffre et al, 2015; Ogura et al, 2023; Roşianu et al, 2023; Tay et al, 2019). We found that *sax-1* acts with *rab-11.2, rabi-1/Rabin8* as well as with *rab-8* and *rab-10* to eliminate IL2Q 2° dendrites post dauer, consistent with a role for SAX-1 in regulating membrane dynamics. The preferential elimination of 2° dendrites in these mutants suggests that membrane trafficking is differentially regulated across branch orders during pruning. We also found that *sax-1* is required for SAX-2 distribution, with an increased abundance of SAX-2 in *sax-1* mutant dendrites. If and how this localization relates to the regulation of membrane trafficking by SAX-1 is unclear. Since some increase in SAX-2 puncta is observed outside the pruning window, it is unlikely that SAX-2 levels are directly instructive for IL2Q pruning. Instead, we hypothesize that SAX-1 and SAX-2 regulate membrane dynamics at baseline levels throughout development and in adults, with their activity heightened during the pruning window. The increased dendritic localization of SAX-2 in *sax-1* mutants could suggest that SAX-2 puncta normally form and dissolve at different stages of membrane trafficking, and that the *sax-1* mutation arrests this process after SAX-2 puncta formation, potentially impairing membrane retrieval. Since the compartment to which SAX-2 localizes is unclear (Park et al, 2024), future work will be required to test this hypothesis.

A key and unexpected finding of this study is that different dendritic branches rely on distinct genetic requirements for their elimination, likely reflecting the partial nature of pruning in this system. Unlike in *Drosophila* da and mushroom body γ neurons, where entire dendritic arbors are eliminated, *C. elegans* IL2Q neurons selectively eliminate only dauer-induced branches. This points to mechanisms that discriminate between primary and higher-order dendrites during pruning. Moreover, the observation that the mode of dauer induction alters the pruning mechanism underscores the complexity of this process. We hypothesize that the differential requirement for *sax-1* between starvation-induced N2 dauers and *daf-2* or *daf-7* dauers reflects divergent characteristics of the dauer state in these backgrounds. *daf-2* and *daf-7* differ from N2 dauers in multiple ways, including their transcriptome and their rate and synchronicity of dauer exit upon return to favorable conditions. We hypothesize that the relevant difference for the underlying dependence on *sax-1* may be the expression of specific cell-surface proteins. These proteins show differential expression between dauer types, and their endocytosis has been shown to be important for dendrite pruning in other systems (Corchado et al, 2026; Zhang et al, 2014).

In summary, we established *C. elegans* IL2Q as a genetically tractable model to study dendrite pruning and identified SAX-1 and its interactors as key regulators of this process. The specificity of pruning in IL2Q for dauer-generated dendrites, along with the branch-specific requirement for *sax-1*, underscores the intricate cell-biological mechanisms that are employed during neuronal remodeling. Leveraging this system in future studies could reveal how physiological states or external cues regulate the cell-biological processes that drive branch-specific elimination programs.

## Methods

**Table:** Reagents and tools table

Reagent/resource	Reference or source	Identifier or catalog number
**Experimental models**		
*Escherichia coli:* OP50 Strain	*Caenorhabditis* Genetics Center (CGC)	OP50
*Escherichia coli:* DH10B, chemical competent cells	ThermoFisher	Cat# 18297010
*myIs13*[*klp-6*p::GFP] (III)	*myIs13* from Schroeder Lab	PT2519
*shyIs66*[*tba-6*p::tagRFP;*tba-6*p::ZIF-1] (III)	This study	MTS1544
*shyIs62*[*egas-1*p::tagRFP] (X)	This study	MTS1531
*daf-7(e1372)* (III); *shyIs66*[*tba-6*p::tagRFP;*tba-6*p::ZIF-1] (III)	This study	MTS3357
*daf-7(e1372)* (III); *shyIs62*[*egas-1*p::tagRFP] (X)	This study	MTS3392
*daf-7(e1372)* (III); *myIs13*[*klp-6*p::GFP] (III)	This study	MTS542
*sax-1(ky491)* (X); *shyIs62*[*egas-1*p::tagRFP] (X)	This study	MTS1463
*sax-2(ky216)* (III); *shyIs62*[*egas-1*p::tagRFP] (X)	This study	MTS1470
*sax-1(ky491)* (X); *daf-7(e1372)* (III); *myIs13*[*klp-6*p::GFP] (III)	This study	MTS3358
*sax-1(shy87)* (X); *daf-7(e1372)* (III); *shyIs66*[*tba-6*p::tagRFP;*tba-6*p::ZIF-1] (III)	This study	MTS1199
*sax-1(shy280)* (X); *daf-7(e1372)* (III); *shyIs66*[*tba-6*p::tagRFP;*tba-6*p::ZIF-1] (III)	This study	MTS3393
*sax-1(shy277)* (X); *daf-7(e1372)* (III); *shyIs66*[*tba-6*p::tagRFP;*tba-6*p::ZIF-1] (III)	This study	MTS3351
shyEx860 [*tba-6*p::SAX-1::GFP;*elt-7*p::eGFP::NLS]; *sax-1(shy87)* (X); *daf-7(e1372)* (III); *shyIs66*[*tba-6*p::tagRFP;*tba-6*p::ZIF-1] (III)	This study	MTS3663
shyEx861 [*tba-6*p::SAX-1::GFP;*elt-7*p::eGFP::NLS]; *sax-1(shy87)* (X); *daf-7(e1372)* (III); *shyIs66*[*tba-6*p::tagRFP;*tba-6*p::ZIF-1] (III)	This study	MTS3664
shyEx862 [*tba-6*p::SAX-1(K116A);*elt-7*p::eGFP::NLS]; *sax-1(shy87)* (X); *daf-7(e1372)* (III); *shyIs66*[*tba-6*p::tagRFP;*tba-6*p::ZIF-1] (III)	This study	MTS3665
shyEx863 [*tba-6*p::SAX-1(K116A);*elt-7*p::eGFP::NLS]; *sax-1(shy87)* (X); *daf-7(e1372)* (III); *shyIs66*[*tba-6*p::tagRFP;*tba-6*p::ZIF-1] (III)	This study	MTS3666
shyEx864 [*dpy-7*p::SAX-1::GFP;*elt-7*p::eGFP::NLS]; *sax-1(shy87)* (X); *daf-7(e1372)* (III); *shyIs66*[*tba-6*p::tagRFP;*tba-6*p::ZIF-1] (III)	This study	MTS3667
shyEx865 [*dpy-7*p::SAX-1::GFP;*elt-7*p::eGFP::NLS]; *sax-1(shy87)* (X); *daf-7(e1372)* (III); *shyIs66*[*tba-6*p::tagRFP;*tba-6*p::ZIF-1] (III)	This study	MTS3668
shyEx866 [*myo-3*p::SAX-1::GFP;*elt-7*p::eGFP::NLS]; *sax-1(shy87)* (X); *daf-7(e1372)* (III); *shyIs66*[*tba-6*p::tagRFP;*tba-6*p::ZIF-1] (III)	This study	MTS3669
shyEx867 [*myo-3*p::SAX-1::GFP;*elt-7*p::eGFP::NLS]; *sax-1(shy87)* (X); *daf-7(e1372)* (III); *shyIs66*[*tba-6*p::tagRFP;*tba-6*p::ZIF-1] (III)	This study	MTS3671
*mob-2(ok3273)* (X); *daf-7(e1372)* (III); *shyIs66*[tba-6p::tagRFP;tba-6p::ZIF-1] (III)	This study	MTS3384
*mob-2(ok3273)* (X); *sax-1(ky491)* (X); *daf-7(e1372)* (III); *shyIs66*[tba-6p::tagRFP;tba-6p::ZIF-1] (III)	This study	MTS3364
*sax-1(ky491)* (X); *daf-7(e1372)* (III); *shyIs62*[egas-1p::tagRFP] (X)	This study	MTS3390
*sax-2(ky216)* (III); *daf-7(e1372)* (III); *shyIs62*[egas-1p::tagRFP] (X)	This study	MTS3350
*sax-2(ky216)* (III); *sax-1(shy280)* (X); *daf-7(e1372)* (III); *shyIs62*[egas-1p::tagRFP] (X)	This study	MTS3393
*sax-2(ky216)* (III); *sax-1(ky491)* (X); *daf-7(e1372)* (III); *shyIs62*[egas-1p::tagRFP] (X)	This study	MTS3347
*sax-2(ky216)* (III); *mob-2(ok3273)* (X); *daf-7(e1372)* (III); *shyIs62*[egas-1p::tagRFP] (X)	This study	MTS3670
wdIs52[*F49H12.4*p::GFP + unc-119(+)] (II)	*wdIs52* from Hammarlund Lab	NC1687
wdIs52[*F49H12.4*p::GFP + unc-119(+)] (II); *daf-7(e1372)* (III)	This study	MTS3672
wdIs52[*F49H12.4*p::GFP + unc-119(+)] (II); *sax-1(ky491)* (X); *daf-7(e1372)* (III)	This study	MTS3673
*sax-1(ky491)* (X); wdIs52[F49H12.4::GFP + unc-119( + )](II)	This study	MTS1981
*sax-2(ky216)* (III); wdIs52[F49H12.4::GFP + unc-119(+)] (II)	This study	MTS3332
*daf-7(e1372)* (III); shyEx349[tba-6p::tagRFP;tba-6p::GFP1-10]	This study	MTS3410
*sax-2(shy279*[*sax-2*::GFP11x3]*)* (III); *daf-7(e1372)* (III); shyEx349[tba-6p::tagRFP;tba-6p::GFP1-10]	This study	MTS3411
*sax-2(shy279*[*sax-2*::GFP11x3]*)* (III); *sax-1(ky491)* (X); *daf-7(e1372)* (III); shyEx349[tba-6p::tagRFP;tba-6p::GFP1-10]	This study	MTS3412
*rab-11.1(tm2251)* (I); *daf-7(e1372)* (III); *shyIs66*[tba-6p::tagRFP;tba-6p::ZIF-1] (III)	This study	MTS3413
*rab-8(tm2991)* (I); *daf-7(e1372)* (III); *shyIs66*[tba-6p::tagRFP;tba-6p::ZIF-1] (III)	This study	MTS3414
*rab-10(ok1494)* (I); *daf-7(e1372)* (III); *shyIs66*[tba-6p::tagRFP;tba-6p::ZIF-1] (III)	This study	MTS3415
*rabi-1(tm2518)* (II); *daf-7(e1372)* (III); *shyIs66*[tba-6p::tagRFP;tba-6p::ZIF-1] (III)	This study	MTS3416
*rabi-1(tm2518)* (II); *sax-1(ky491)* (X); *daf-7(e1372)* (III); *shyIs66*[tba-6p::tagRFP;tba-6p::ZIF-1] (III)	This study	MTS3359
*rab-11.2(tm2081)* (I); *daf-7(e1372)* (III); *shyIs66*[tba-6p::tagRFP;tba-6p::ZIF-1] (III)	This study	MTS3417
*rab-11.2(tm2081)* (I); *sax-1(ky491)* (X); *daf-7(e1372)* (III); *shyIs66*[tba-6p::tagRFP;tba-6p::ZIF-1] (III)	This study	MTS3418
shyEx828 [tba-6p::spGDP::mCD8::tagRFP, myo-3p::sp::scFV::GFP]; *daf-7(e1372)* (III)	This study	MTS3419
shyEx828 [tba-6p::spGDP::mCD8::tagRFP, myo-3p::sp::scFV::GFP]; *sax-1(ky491)* (X); *daf-7(e1372)* (III)	This study	MTS3420
shyEx829 [tba-6p::mCD8::tagRFP, myo-3p::sp::scFV::GFP]; *daf-7(e1372)* (III)	This study	MTS3422
shyEx829 [tba-6p::mCD8::tagRFP, myo-3p::sp::scFV::GFP]; *sax-1(ky491)* (X); *daf-7(e1372)* (III)	This study	MTS3423
*yap-1(tm1416)* (X); *daf-7(e1372)* (III); *shyIs66*[tba-6p::tagRFP;tba-6p::ZIF-1] (III)	This study	MTS3421
*rict-1(mg360)* (II); *daf-7(e1372)* (III); *shyIs66*[tba-6p::tagRFP;tba-6p::ZIF-1] (III)	This study	MTS3424
*unc-82(e1323)* (II); *daf-7(e1372)* (III); *shyIs66*[tba-6p::tagRFP;tba-6p::ZIF-1] (III)	This study	MTS3425
*unc-43(e408)* (IV); *daf-7(e1372)* (III); *shyIs66*[tba-6p::tagRFP;tba-6p::ZIF-1] (III)	This study	MTS3426
*wsp-1(gm324)* (IV); *daf-7(e1372)* (III); *shyIs66*[tba-6p::tagRFP;tba-6p::ZIF-1] (III)	This study	MTS3427
*sel-5(ok363)* (III); *daf-7(e1372)* (III); *shyIs66*[tba-6p::tagRFP;tba-6p::ZIF-1] (III)	This study	MTS3366
*pifk-1(tm2348)* (X); *daf-7(e1372)* (III); *shyIs66*[tba-6p::tagRFP;tba-6p::ZIF-1] (III)	This study	MTS3428
*mig-10(ok2499)* (III); *daf-7(e1372)* (III); *shyIs66*[tba-6p::tagRFP;tba-6p::ZIF-1] (III)	This study	MTS3381
*F58G6.1/amph-1(tm1060)* (IV); *daf-7(e1372)* (III); *shyIs66*[tba-6p::tagRFP;tba-6p::ZIF-1] (III)	This study	MTS3389
*pink-1(ok3538)* (II); *daf-7(e1372)* (III); *shyIs66*[tba-6p::tagRFP;tba-6p::ZIF-1] (III)	This study	MTS3429
**Recombinant DNA**		
*tba-6*p::tagRFP	This study	pGLS8
*tba-6*p::ZIF-1	This study	pGLS9
*tba-6*p::spGFP1-10	This study	pOVG6
*egas-1*p::tagRFP	This study	pPFD12
*tba-6*p::SAX-1::GFP	This study	pPFD33
*tba-6*p::SAX-1(K116A)	This study	pPFD38
*dpy-7*p::SAX-1::GFP	This study	pPFD46
*myo-3*p::SAX-1::GFP	This study	pPFD45
*tba-6*p::spGDP::mCD8::tagRFP	This study	pPFD57
*myo-3*p::sp::scFV::GFP	Richardson Lab	pCER229
*tba-6*p::mCD8::tagRFP	This study	pPFD74
**Oligonucleotides and other sequence-based reagents**		
For *sax-1(shy87N316G)* gRNA targeting sequence ( + PAM): TGTGGAAGCTCAGAGCAGAATGG	Sigma-Aldrich	olPFD140
For *sax-1(shy87N316G)* ssODN repair template: tgtaccaacactcttcaaaacttaataattttcagGCTACTCACCATTCTGCTCTGAGCTTCCACAAGAAACGTAT	Sigma-Aldrich	olPFD142
For *sax-1(earlyStop)* gRNA targeting sequence ( + PAM): GGAAATATCGCAGTACACAAAGG	Sigma-Aldrich	olPFD196
For *sax-1(earlyStop)* ssODN repair template: AGAAATTGCACCGGAAATGGAAATATCGCAGTACAGGAAATGGAAATATCGCAGTACAataattaGGATCCctaattaaCAAAGTATAAGgtaatttcattattagaattcgaaacatta	Sigma-Aldrich	olPFD197
For site-directed mutagenesis of *sax-1* cDNA (S279A): tcgcgcatatgcatacgctacggtcggaactc	Sigma-Aldrich	olPFD333
For *sax-2::3xspGFP11* gRNA targeting sequence ( + PAM): TTAATCATAATGATCTGATGAGG	Sigma-Aldrich	olPFD343
For *sax-2::3xspGFP11* dsODN repair template: AGCGTCAATGACCGAATCATTCGCACAATTGCCTTACTCATGGTGGCTCTGGAGGTCGTGACCACATGGTCCTTCATGAGTATGTAAATGCTGCTGGGATTACAGGTGGCTCTGGAGGTAGAGATCATATGGTTCTCCACGAATACGTTAACGCCGCAGGCATCACTGGCGGTAGTGGAGGACGCGACCATATGGTACTACATGAATATGTCAATGCAGCCGGAATAACCTAACAGATCATTATGATTAAattagtggaattgcgttg	IDT	olPFD344
**Chemicals, enzymes and other reagents**		
Ethyl methanesulfonate	Sigma-Aldrich	Cat# M0880-1G
Sodium Dodecyl Sulfate	AmericanBio	Cat# AB01920
*S. pyrogenes* Cas9 3NLS, 10 μg/μL	IDT	Cat# 1081058
Levamisole Hydrochloride	ChemCruz	Cat# 205730
EnGen sgRNA Synthesis Kit, *S. pygenes*	NEB	Cat# E3322S
QuikChange Lightning Multi-Site-Directed Mutagenesis Kit	Agilent	Cat# 210513
Monarch RNA Cleanup Kit	NEB	Cat# T2040
**Software**		
MiMOD	Galaxy	https://toolshed.g2.bx.psu.edu
Prism 10	GraphPad	https://www.graphpad.com/
Illustrator 29.0	Adobe	https://www.adobe.com/products/illustrator.html
ImageJ2 (2.14.0/1.54 f)	NIH	https://imagej.net/software/imagej2/
AlphaFold	Google DeepMind EMBL-EBI	https://alphafold.ebi.ac.uk
Zotero7	Zotero	https://www.zotero.org/download/

### *C. elegans* strains and maintenance

All *C. elegans* strains were cultured on Nematode Growth Medium (NGM) seeded with *Escherichia coli* OP50. Animals were examined at multiple developmental stages, including normal development adults, dauer larva, 16–20 h post dauer larva, and post dauer adults. Normal development adults were grown at 15 °C. Dauer arrest was induced and dauer larva were maintained at 25 °C. Post dauer animals were transferred to and maintained at 15 °C, unless otherwise indicated. A detailed list of *C. elegans* strains used in the study is provided in the Reagents and Tools Table.

### Molecular cloning

Plasmids were constructed using conventional restriction-ligation methods or Gibson Assembly. Genes of interest were ordered as gene fragments from IDT and PCR cloned. Mutagenesis was performed using the QuikChange Lightning Multi-Site-Directed Mutagenesis Kit (Agilent, Cat# 210513). Plasmids were confirmed by Sanger Sequencing. A list of plasmids used in this study are listed in the Reagents and Tools Table. pCER229 was a gift from Claire Richardson. Plasmids were injected at the following concentrations to generate transgenic *C. elegans* lines: pRF4 (40/μL), pGLS8 (15 ng/μL), pGLS9 (10 ng/μL), pOVG6 (15 ng/μL), pPFD12 (40 ng/μL), pPFD33 (10 ng/μL), pPFD38 (10 ng/μL), pPFD45 (10 ng/μL), pPFD46(10 ng/μL), pPFD57 (5 ng/μL), pCER229 (10 ng/μL), and pPFD74 (5 ng/μL). Plasmid sequences and constructs are available upon request.

### *C. elegans* transgenic strain generation

Transgenic strains were generated by microinjecting plasmid DNA into the gonads of young adult animals following established protocols (Mello and Fire, 1995). Plasmids containing *unc-122p::eGFP, unc-122p::tagRFP, elt-7p::eGFP::NLS*, and *elt-7p::TagRFP-T::NLS* were used as co-injection markers. Following microinjection, F1 progeny transmitting the fluorescent reporter or co-injection marker were individually isolated, and their F2 progeny were screened under a compound microscope to identify appropriate expression levels. Integrated strains were generated using the TMP/UV method, outcrossed a minimum of six times, and subsequently mapped to specific chromosomes using standard mapping strains prior to experimental use.

### Dauer induction and exit

#### Starvation

Dauer formation was induced by allowing worms to starve on plates at 20 °C. To select for a large number of dauer larvae, starved plates were washed with M9 and pelleted at 1500 rpm for 3 min. The pellet was resuspended in fresh 1% sodium dodecyl sulfate (SDS). Worms were then incubated in 1% SDS for approximately 30 min (Cassada and Russell, 1975) on a rotator shaker at a slow setting. The treated worms were subsequently subjected to five washes with M9 to remove residual SDS. This treatment selectively eliminated non-dauer larvae and partial dauers, leaving a population of ‘true’ dauers for analysis. For dauer recovery, animals were transferred to fresh NGM plates seeded with *E. coli* OP50 to resume reproductive development or unseeded for dauer analyses. For a small selection of dauer larvae, morphologically distinct larvae were picked from starved plates, using an eyebrow pick, and transferred into a 200 µL drop of fresh 1% SDS within a 35 mm × 15 mm Petri dish. Worms were incubated in 1% SDS for 30 min on a rotator shaker at a slow setting. Surviving dauer larvae were then manually transferred to a fresh NGM plate. Alternatively, a 22 × 22 mm coverslip can be placed within a 60 mm × 15 mm Petri dish to serve as the surface for 200 µL drop of 1% SDS, facilitating dauer transfer to unseeded NGM plates by washing the worms off with 500 µL of M9. Then, manually transferring the dauer larvae to fresh NGM plates.

#### Temperature-sensitive *Daf* constitutive alleles

Dauer induction and exit were controlled using the temperature-sensitive, constitutive *daf-7/TGFβ* mutant allele *e1372*. Larva 4 (L4) stage animals were maintained at 25 °C to allow dauer induction. Then, dauer larva were manually transferred to NGM plates with freshly seeded *E. coli* OP50 and shifted to 15 °C to prompt dauer exit towards reproductive development. The same strategy was employed for constitutive *daf-2*/*InsR* mutant allele *e1370*.

### Genetic screen to isolate IL2Q pruning regulators

To identify novel IL2Q neuron pruning regulators, we conducted an unbiased forward genetic screen (Fig. EV1C). Animals (L4 larva) carrying the temperature-sensitive *daf-7(e1372)* mutant allele were exposed to ethyl methanesulfonate (EMS) following previous protocols (Brenner, 1974) to induce random genomic point mutations. After mutagenesis, animals were maintained at 15 °C to allow for normal development. F1 animals (~ 3 per plate) were then transferred to 25 °C to induce dauer arrest in F2 progeny. Dauer larvae were then manually transferred to NGM plates with freshly seeded *E. coli* OP50 and incubated at 15 °C to prompt dauer exit. Nematodes at the post dauer day 1 adult stage were subsequently mounted on a 2% agarose pad, immobilized with 10 mM levamisole dissolved in M9, and screened under a fluorescent compound microscope for IL2Q remodeling defects. Mutant candidates were isolated and outcrossed at least once to our control strain to confirm the heritability of the observed phenotypes. The causal variant for *shy87*, described in this study, were mapped by whole-genome sequencing and SNP linkage mapping with the MiModD software package run in a Galaxy server. To further confirm the identity of the isolated shy87 allele, we employed a transgenic rescue strategy utilizing a *sax-1* containing fosmid and were able to successfully rescue the mutant phenotype.

### IL2Q remodeling timeseries

Synchronized populations of dauer larva induced at 25 °C, carrying a temperature-sensitive *daf-7(e1372)* mutation, were manually transferred to NGM plates with freshly seeded *E. coli* OP50 and incubated at 15 °C to prompt dauer exit towards reproductive development. At 8 h post-transfer, we screened under a dissecting microscope and selected for larva that exhibited pharyngeal pumping and foraging behavior. The larvae were then kept at 15 °C for further examination. Animals were then mounted on 2% agarose pads and 10 mM levamisole dissolved in M9 for imaging at defined timepoints (12, 14, 16, 18, 20, 22, 24, 26, 28, 30 h). Image acquisition settings were maintained throughout all timepoints. Quantification of remodeling events was performed as described in the “IL2Q neurite scoring” section.

### Fluorescence microscopy and sample preparation

Nematodes were mounted on a 2% agarose pad and paralyzed in a droplet of 10 mM Levamisole dissolved in M9 buffer. Animals were imaged at the following developmental stages: adults (normal development), dauer larva, 12–13 h post dauer larva, 16–20 h post dauer larva, L4, and post dauer 1-day-old adults, as indicated in the Figures. Images were acquired with a Laser Sage DMi8 inverted microscope (Leica) equipped with a VT-iSIM system (BioVision) and an ORCA-Flash 4.0 camera (Hamamatsu) controlled by MetaMorph Advanced Confocal Acquisition Software Package or VisiView® Software. The microscope was equipped with an HC PL APO 100×/1.47NA Oil, HC PL APO 63×/1.40NA Oil CS2, a HC PL APO 40×/1.30NA Oil CS2, and a HC PL APO 20×/0.8NA Air objective. Cytosolic GFP or TagRFP reporters were imaged using a 488 nm and 561 nm laser, respectively, with a 100 ms exposure time. Endogenous SAX-2 puncta were visualized using a 488 nm laser excitation with an exposure time of 200 ms. Endocytic events were imaged using a 488 nm laser excitation for GFP (100 ms exposure time) and 561 nm laser excitation for mCD8::TagRFP (100 ms exposure time). Z-stacks were acquired at step sizes ranging from 0.2 to 1.0 µm. Maximum intensity projections were generated in FIJI (ImageJ2).

### CRISPR/Cas9 genome editing

All CRISPR-Cas9-engineered strains were generated following the Mello Lab published protocol (Dokshin et al, 2018). We synthesized sgRNA from single-stranded DNA utilizing the EnGen sgRNA Synthesis Kit, *S. pygenes* (NEB Cat#E3322S), followed by sgRNA purification utilizing the Monarch RNA cleanup kit (NEB #T2040). A detailed list of sgRNA target sites is provided in the Reagents and Tools Table, along with corresponding repair templates listed.

#### *sax-1(shy280)*

Utilizing CRISPR-Cas9, we engineered a *sax-1* allele with an early stop codon by designing guide RNAs targeting the first exon. Homology-directed repair with a single-stranded DNA donor template (ssODN) introduced the premature stop sequence into the locus. *daf-7(e1372)* worms were microinjected with a mixture containing synthesized sgRNA (91.5 pmol/3700 ng), ssODN (1 µg/µl), Cas9 protein (30.6 pmol/5.02 µg), pRF4 (40 ng/µl) as a co-injection marker, and nuclease-free water to a final volume of 20 µl. sgRNA target site provided in the Reagents and Tools Table. Following microinjection, P0 worms were single-picked onto seeded NGM plates, and F1 progeny were screened for rollers to assess injection efficiency. We then selected up to three parental plates with the highest number of rollers Paix et al, 2017. Non-roller F1 worms from these plates were then single-picked and allowed to lay progeny.

#### *sax-1(shy277)*

We utilized CRISPR-Cas9 to repair the EMS-induced point mutation N316 > G in *sax-1(shy87)* mutants. The sgRNA target site and ssODN containing the desired point mutation edit are provided in the Reagents and Tools Table. Microinjections were performed in *sax-1(shy87);daf-7(e1372)* mutant worms, as previously described.

#### *sax-2(shy279)*

We engineered a *sax-2* allele utilizing CRISPR-Cas9 to insert three copies of the split-GFP tag (3xGFP11) at the C-terminus, within the last exon. Homology-directed repair was achieved using a double-stranded DNA donor template (dsODN) that was synthesized as a gene fragment ordered through IDT. sgRNA and dsODN repair template sequences are listed in the Reagents and Tools Table. Microinjections were performed in a strain expressing GFP1-10 under the control of the pan-neuronal *rab-3* promoter. Following screening for successful CRISPR-Cas9 edits as described above, we crossed the generated *sax-2(shy279)* allele into the *daf-7(e1372);shyEx349[tba-6*p::tagRFP*;tba-6*p::spGFP1-10*]* strain to visualize IL2 neuron-specific expression of endogenous SAX-2.

### IL2Q neurite scoring

Raw imaging files were imported to ImageJ2 for manual IL2Q neurite scoring. Scoring was based on the stereotypical arborization pattern of IL2Q neurons (Fig. EV1A). In control animals, primary (1°) dendrites extend anteriorly from the cell body toward the nose along the pharyngeal axis. Secondary (2°) dendrites emerge perpendicularly from the 1° dendrite, extending toward the ventral and dorsal midlines. Tertiary (3°) dendrites arise from the 2° dendrites and bifurcate along the anterior-posterior axis. Quaternary (4°) dendrites extend perpendicularly from the 3° dendrites toward the body-wall muscle quadrants. In addition to anterior arborization, neurons also project posteriorly directed neurites, but these were excluded for all analysis conducted at each developmental stage. For each animal, IL2D and IL2V pairs (four) neurons and all dendrite orders (1°–4°) were scored, and data were compiled to determine the total number of 2°, 3°, and 4° dendrites per genotype. For all post dauer animals studied, the previously described scoring strategy was employed.

To validate pruning in *shy87* mutant, a pruning index was calculated (Fig. 1I) using the following equation:

$${Pruning}\,{Index}=1-\left[\frac{{\Delta {shy}87}_{\left({Dauer}{Median}-{Post}{Dauer}{Adult}{Median}\right)}}{{\Delta {Control}}_{\left({Dauer}{Median}-{Post}{Dauer}{Adult}{Median}\right)}}\right]$$

Measurements were calculated separately for each branch order (2°, 3°, and 4°). For each branch order, the median value at the dauer stage was compared to the median value at the post dauer adult stage. The resulting ratio represents the relative change between developmental stages in *shy87* mutants normalized to the corresponding change observed in control animals. Values approaching zero indicate pruning events that are consistent to those observed in the control condition, whereas larger values indicate reduced pruning relative to control.

### PVD neurite scoring

PVD multi-dendritic neurons were visualized under a fluorescent compound microscope in worms expressing GFP under the control of the *F49H12.4* promoter. Scoring was conducted on young adult worms, and the stereotypical dendritic “menorah” branch order (2°–4°) was quantified. PVD primary dendrites extend along the anterior-posterior axis, from which secondary dendrites branch laterally at 90°. Tertiary dendrites extend from secondary dendrites and further bifurcate into quaternary dendrites, each extending at ~90° respective to the preceding order. Both anterior and posterior dendritic branch orders were scored and included in the quantifications. Images of representative PVD neurons were stitched pair-wise using a FIJI (ImageJ2) plug-in, and maximum intensity projections were generated from the resulting composite images (Preibisch et al, 2009).

### Fluorescence quantification

Following acquisition, raw imaging files were processed in ImageJ2. For quantification of endogenous and cell-specific SAX-2 puncta in control and *sax-1* mutants, we manually counted GFP(+) puncta across the length of the IL2D/V primary (1°) dendrite and higher-order dendrites (1°–4°). For quantification of the generic endocytosis reporter, normalized fluorescence line scans were generated by tracing the length of an IL2D/V primary (1°) dendrite and measuring fluorescence intensity along the neurite using the *Plot Profile* function. Colocalized puncta between secreted GFP and IL2-expressed mCD8::tagRFP were manually counted from images of live animals. Only puncta colocalized in IL2Q dendritic arbor were considered for quantitative analysis and statistical testing. For Figs. 4 and  6, individual neurons were counted as independent observations (*n*) when quantifying SAX-2 or colocalized GFP and mCD8::tagRFP puncta, since the mosaicism of the extrachromosomal array led to less than 4 cells expressing the markers in each animal.

### Statistical analysis

Statistical analyses were performed on GraphPad Prism 10. Data were tested for normality, and parametric or nonparametric tests used as appropriate. Statistical significance was determined using unpaired *t* test, unpaired *t* test with Welch’s correction, or Mann–Whitney *U* test. For multiple comparisons, we used Brown–Forsythe and Welch ANOVA test or Kruskal–Wallis test followed by a post test. Sample sizes are described in figure legends. Error bars are represented as ±SEM, with individual data points shown.

### Materials availability

Plasmids and transgenic *C. elegans* strains generated for this study are available from the lead contact upon request.

## Supplementary information

Peer Review File

Source data Fig. 1

Source data Fig. 2

Source data Fig. 3

Source data Fig. 4

Source data Fig. 5

Source data Fig. 6

Figure EV1 Source Data

Figure EV2 Source Data

Figure EV3 Source Data

Figure EV4 Source Data

Expanded View Figures

## Data Availability

Raw data used for this study are available from the lead contact upon request. The source data of this paper are collected in the following database record: biostudies:S-SCDT-10_1038-S44318-026-00841-w.
