# Supplementary material for: NDR kinase SAX-1 controls dendrite branch-specific elimination during neuronal remodeling in C. elegans
Source: EMBO J. 2026 Jun 27;45(15):5267–91. doi: 10.1038/s44318-026-00841-w (PMC13434639; doi:10.1038/s44318-026-00841-w)
Supplement: Supplementary file 1 [file 44318_2026_841_MOESM12_ESM.pdf]

Expanded View Figures

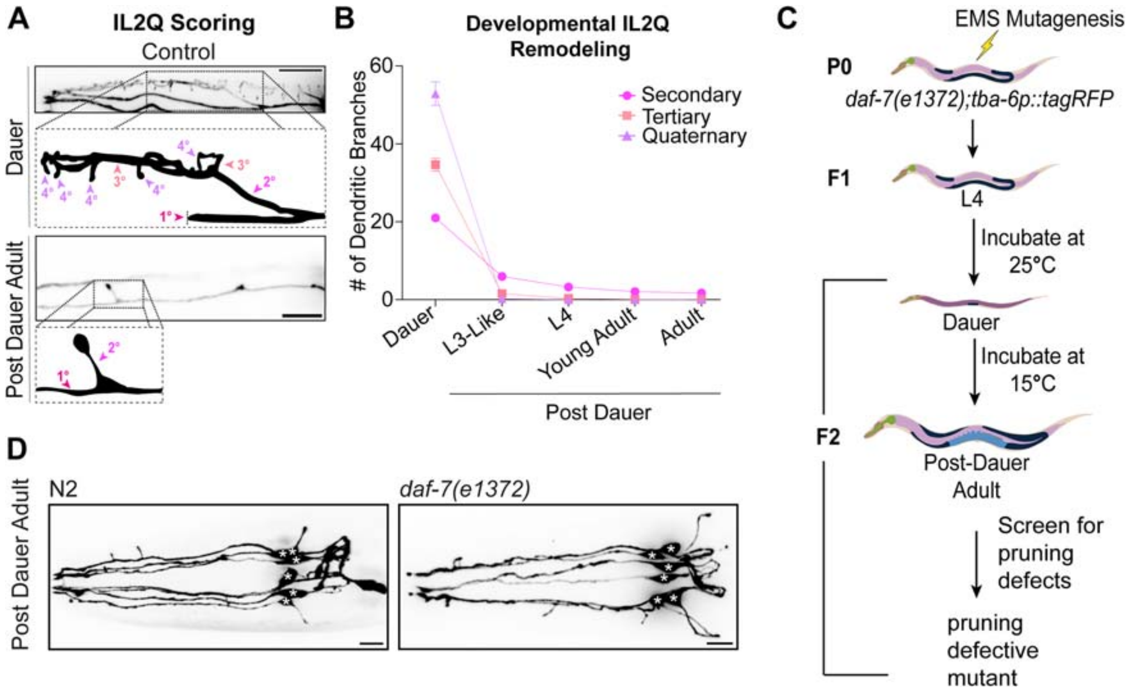

Figure EV1. Experimental paradigm for IL2Q neuronal remodeling and scoring strategy.

(**A**) IL2Q scoring: Representative Z-projection insets of select IL2Q neurons in control animals at dauer and post dauer adults, with schematic overlays indicating (with arrowhead) branch order (1°–4°) used for scoring dendrites. Control insets were intentionally reused from Fig. 1C,D to demonstrate the scoring approach used for quantification. Scale bar, 10 µm. (**B**) Line graph depicting the progressive elimination of IL2Q higher-order dendrites following dauer recovery towards adulthood, $n = 11\text{-}53$. (**C**) Schematic depicting strategy for the genetic screen. (**D**) Representative maximum intensity Z-projections of post dauer adults in N2 (left) and *daf-7(e1372)* mutants (right) show no observable difference in post dauer adults IL2Q morphology or pruning. Asterisks represent IL2 cell bodies. Scale bar, 10 µm. Source data are available online for this figure.

© The Author(s)

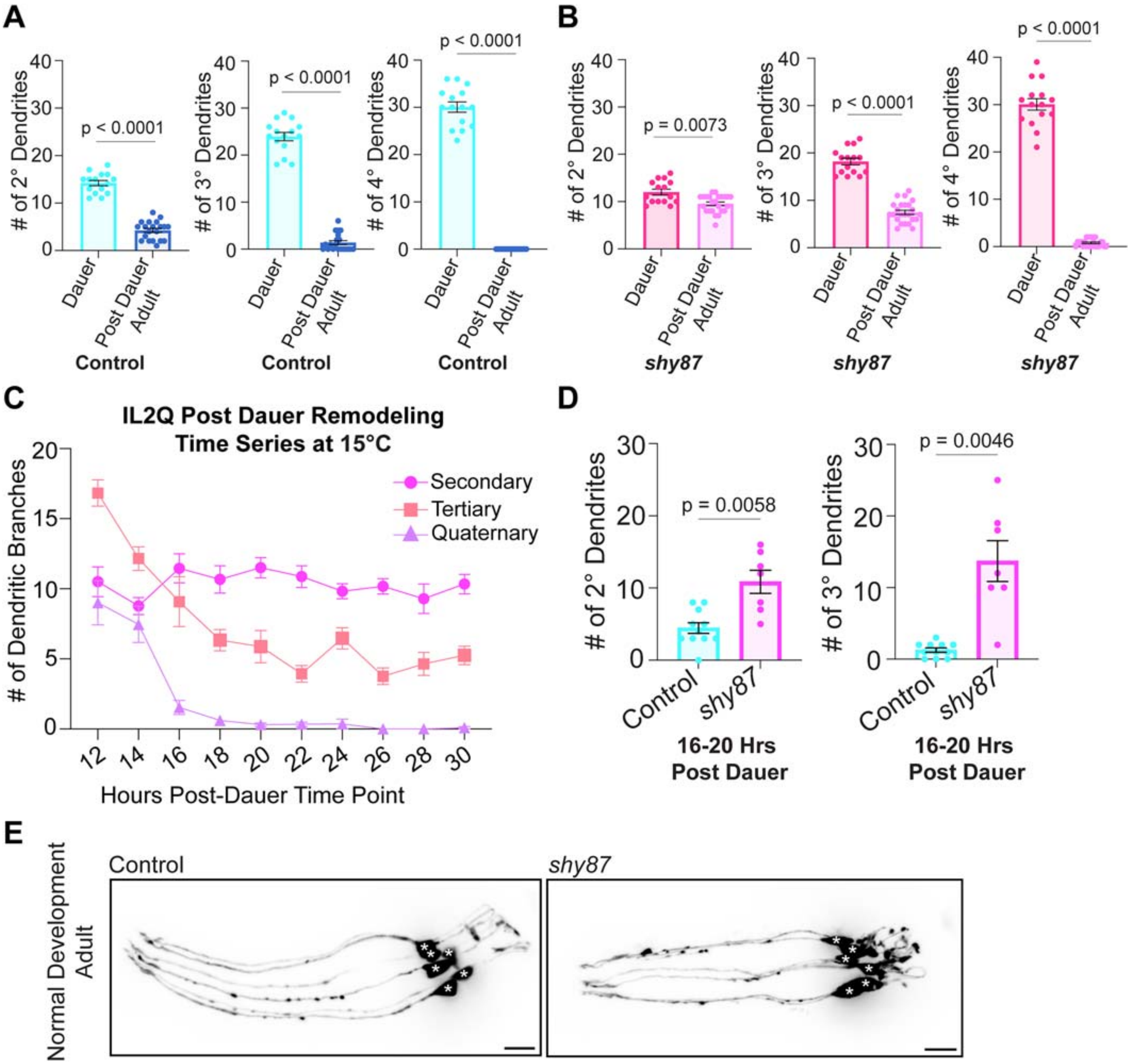

Figure EV2. ***sax-1* is required for branch elimination, not inhibiting branch growth.**

(**A**) Internal comparison of the total number of neurites in control dauer ($n = 15$) versus control post dauer adults ($n = 20$), whereas there is a significant reduction in the number of IL2Q in post dauer adults when compared to dauer. Suggesting successful elimination of the IL2Q dendritic arbor post-dauer. Statistical significance was determined using a Mann–Whitney test for control 3° and 4° dendrites and Welch's *t* test for 2° dendrites. Statistical significance was determined using a Mann–Whitney test for control 3° and 4° dendrites and Welch's *t* test for 2° dendrites. Error bars represent ±SEM. (**B**) Internal comparison of the total number of IL2Q neurites in *shy87* mutant dauers ($n = 15$) versus post dauer adults ($n = 24$). The number of 2° dendrites in post dauer adult *shy87* mutants remained comparable to that of *shy87* mutant dauers; meanwhile, 3° dendrites slightly decreased in number and 4° dendrites were entirely eliminated in post dauer adults. Suggesting a maintenance of the dendritic arbor, specifically 2° dendrites and a subset of 3° dendrites post dauer. Statistical significance was determined using a Mann–Whitney test for control 2° and 3° dendrites and Welch's *t* test for 3° dendrites. Error bars represent ±SEM. (**C**) Line graph depicting the elimination of IL2Q higher-order dendrites over time (hours) post dauer (transfer to 15°C) in control animals, $n = 6$–17. Error bars represent ±SEM. (**D**) Quantification of total 2° and 3° dendrite numbers in control ($n = 11$) and *shy87* mutants ($n = 7$) at 16–20 h post dauer. Unpaired *t* test with Welch's correction. Error bars represent ±SEM. (**E**) Representative confocal images of adult control and *shy87* mutant animals under normal reproductive development. Asterisks indicate IL2 cell bodies. Scale bar, 10 μm. Source data are available online for this figure.

© The Author(s)

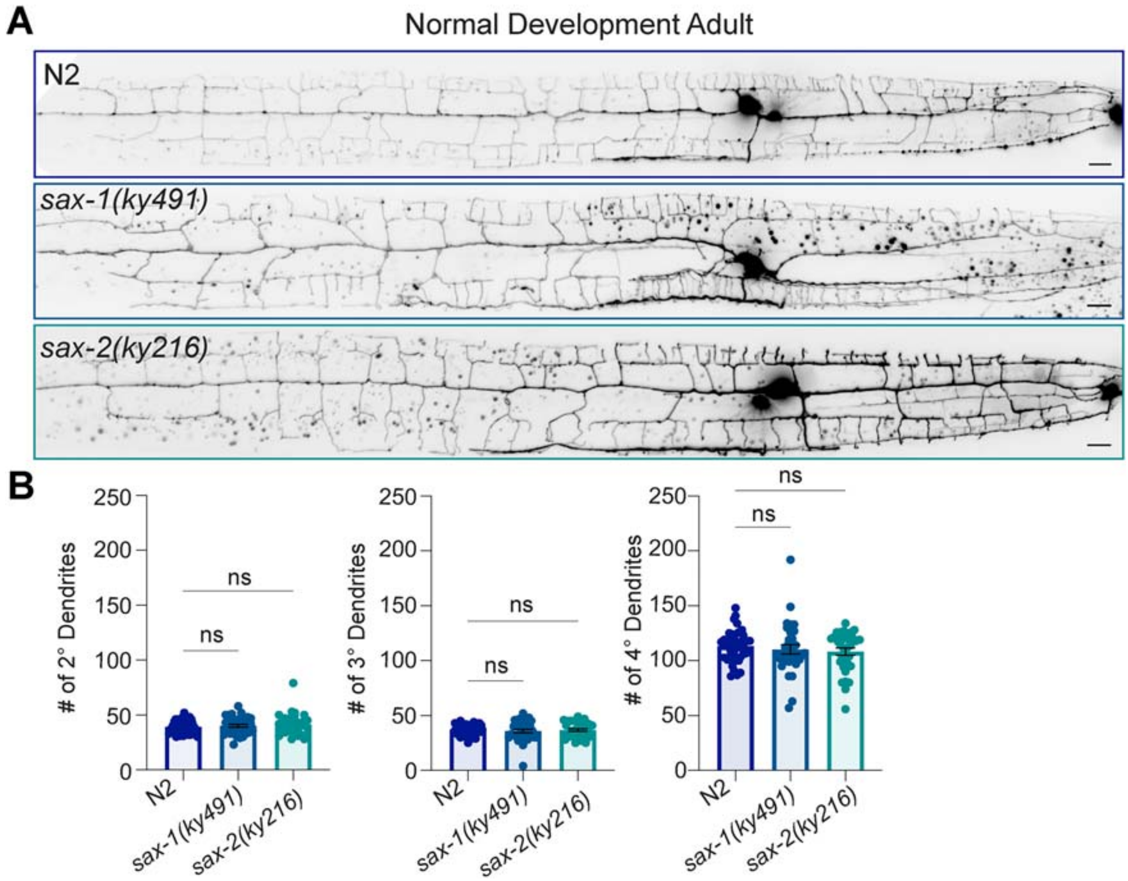

Figure EV3. SAX-1 and SAX-2 are not required for PVD neuron branching.

(**A**) Representative images of polymodal PVD neurons in N2 ($n = 40$), *sax-1* ($n = 33$), and *sax-2* ($n = 33$) mutant animals. Scale Bar, 10 µm. (**B**) Quantifications of total number of PVD dendrite orders (2°–4°). Brown–Forsythe and Welch ANOVA with Dunnett's multiple comparison. Error bars are ±SEM. Source data are available online for this figure.

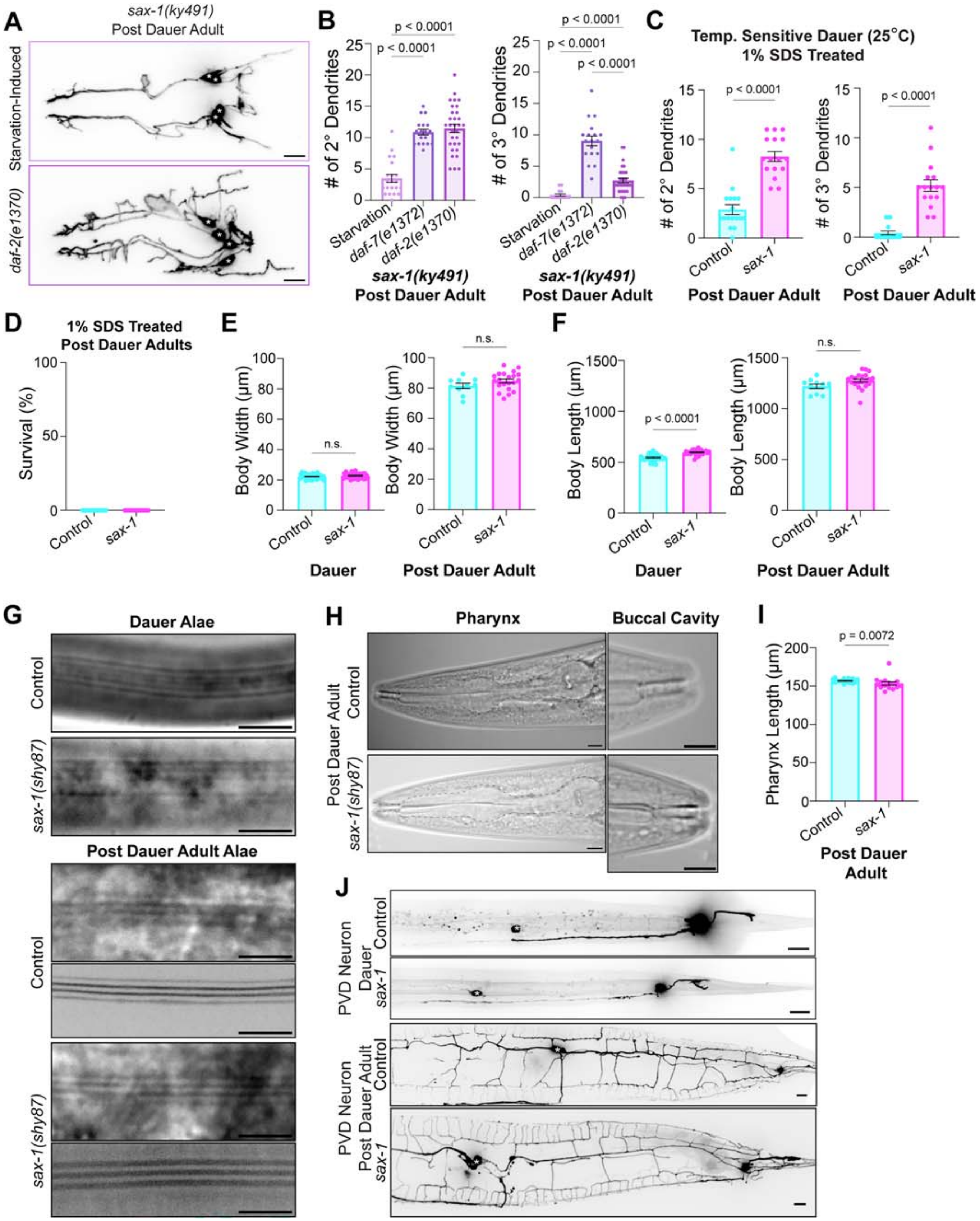
A
sax-1(ky491)
Post Dauer Adult
Starvation-Induced
daf-2(e1370)
B
p < 0.0001
p < 0.0001
# of 2° Dendrites
Starvation
daf-7(e1372)
daf-2(e1370)
sax-1(ky491)
Post Dauer Adult
p < 0.0001
p < 0.0001
p < 0.0001
# of 3° Dendrites
C
Temp. Sensitive Dauer (25°C)
1% SDS Treated
p < 0.0001
# of 2° Dendrites
p < 0.0001
# of 3° Dendrites
Control
sax-1
Post Dauer Adult
Post Dauer Adult
D
1% SDS Treated
Post Dauer Adults
Survival (%)
E
Body Width (µm)
n.s.
Dauer
Post Dauer Adult
F
Body Length (µm)
p < 0.0001
n.s.
G
Dauer Alae
Control
sax-1(shy87)
Post Dauer Adult Alae
H
Pharynx
Buccal Cavity
Post Dauer Adult
I
p = 0.0072
Pharynx Length (µm)
Post Dauer
Adult
J
PVD Neuron
Dauer
PVD Neuron
Post Dauer Adult

Figure EV4. Validation of dauer exit in *sax-1* mutants.

(**A**) Z-projection of *sax-1(ky491)* post dauer adults following dauer induction via starvation (top) or a dauer constitutive mutant, *daf-2/Insulin-receptor* (bottom). Scale bar, 10 µm. (**B**) Quantification of 2° and 3° dendrites of *sax-1(ky491)* mutants following dauer induction via *daf-7(e1372)* ($n = 18$), *daf-2(e1370)* ($n = 31$), and starvation conditions ($n = 21$). Brown–Forsythe and Welch ANOVA with Dunnett's correction. Error bars are ±SEM, with individual data points shown. (**C**) Quantification of 2° and 3° dendrites in control ($n = 17$) and *sax-1* mutant ($n = 16$) post dauer adults. Dauers were treated with 1% SDS for true dauer selection and exclusion of partial dauers. Mann–Whitney U test. Error bars represent ±SEM. (**D**) Quantification of the percentage of post dauer adults that survived 1% SDS treatment. (**E**, **F**) Quantifications of body width (µm) and length (µm) in control ($n = 33$ and *sax-1* mutant ($n = 28$) animals at dauer and post dauer adults. Statistical significance was determined using Welch's *t* test or unpaired *t* test. Error bars represent ±SEM. (**G**) Micrographs of dauer and post dauer adult alae for control and *sax-1* mutants. Scale bar, 10 µm. (**H**) Micrographs of post dauer adult control and *sax-1* mutant pharynx and buccal cavity (zoomed inset). Scale bar, 10 µm. (**I**) Quantification of pharynx length of control ($n = 16$) versus *sax-1* mutants ($n = 15$). Mann–Whitney *U* test. Error bars represent ±SEM. Post dauer adults exhibit normal pharyngeal and buccal cavity morphology, suggesting no persistent morphological differences from dauer arrest. (**J**) Representative micrographs of the multi-dendritic neuron PVD (mid to posterior dendrite) in dauer and post dauer adults of control and *sax-1* mutants. Scale bar, 10 µm. PVD remains unbranched at dauer and branches normally after resuming reproductive development, suggesting no stark post dauer branching defects. Source data are available online for this figure.

© The Author(s)
