# Supplementary material for: NDR kinase SAX-1 controls dendrite branch-specific elimination during neuronal remodeling in C. elegans
Source: EMBO J. 2026 Jun 27;45(15):5267–91. doi: 10.1038/s44318-026-00841-w (PMC13434639; doi:10.1038/s44318-026-00841-w)
Supplement: Supplementary file 2 [file 44318_2026_841_MOESM1_ESM.pdf]

NDR kinase SAX-1 controls dendrite branch-specific pruning during neuronal remodeling in C. elegans

Paola Figueroa Delgado and Shaul Yogev

Corresponding authors: Shaul Yogev (shaul.yogev@yale.edu)

Review Timeline:

Transferred from Review Commons:	22nd Aug 25
Editorial Decision:	1st Sep 25
Revision Received:	17th Mar 26
Editorial Decision:	5th May 26
Revision Received:	29th May 26
Accepted:	11th Jun 26

Editor: Ieva Gailite

Transaction Report:

This manuscript was transferred to The EMBO Journal following peer review at Review Commons.

(Note: With the exception of the correction of typographical or spelling errors that could be a source of ambiguity, letters and reports are not edited. Depending on transfer agreements, referee reports obtained elsewhere may or may not be included in this compilation. Referee reports are anonymous unless the Referee chooses to sign their reports.)

Review #1

1. Evidence, reproducibility and clarity:

Evidence, reproducibility and clarity (Required)

This interesting study uses an unbiased genetic screen in C. elegans to identify SAX-1/NDR kinase as a regulator of dendritic branch elimination. Loss of SAX-1 results in an excess branching phenotype that is striking and highly penetrant. The authors identify several additional regulators of branch elimination (SAX-2, MOB-1, RABI-1, RAB-11.2) by using a candidate genetic screen aimed at factors that interact physically or genetically with SAX-1. They propose that SAX-1 acts by promoting membrane retrieval based on the nature of these interactors and the results of an imaging-based in vivo assay for endocytic puncta.

Major comments.

1. My biggest concern is that the phenotypes are only observed in temperature-sensitive dauer-constitutive mutant backgrounds, and not in wild-type dauers. That is, wild-type animals exiting dauer do not require SAX-1 for dendrite elimination.

While this does not undermine the importance of the results, it does require more explanation. The authors write that "the requirement for sax-1... relies on specific physiological states of the dauer stage," but I do not understand what this means. Are they saying that daf-7 and daf-2 dauers are in a different "physiological state" than wild-type dauers? In what way? What is the evidence for this? A more rigorous explanation is needed.

To me, the simplest genetic explanation is that daf-7 and daf-2 are partially required for branch retraction in a manner redundant with sax-1, and the ts mutants are not fully wild-type at 15C. Thus, the sax-1 requirement is revealed only in these mutant backgrounds. Can the authors examine starvation-induced dauers of daf-7 or daf-2 raised continuously at 15C?

daf-7 and daf-2 ts strains can form "partial dauers" that have a dauer-like appearance but are not SDS resistant. Could the difference between partial dauers and full dauers account for the difference in sax-1-dependence? The authors could use SDS selection of the daf-7 strain at 25C to ensure they are examining full dauers.

The Bargmann lab has created a daf-2 FLP-OUT strain (ky1095ky1087) that allows cell-type-specific removal of daf-2. Could this be used to test for a cell-autonomous role of daf-2 in IL2Q related to branch elimination?

These ideas are not a list of specific experiments the authors need to complete, rather they are meant to illustrate some possible approaches to the question. Whatever approach they use, it is important for them to more rigorously explain why SAX-1 is not required for branch removal in wild-type animals.

2. The SAX-2 localization (Fig. 4) and endocytosis assay (Fig. 6) results were not clear to me

from the data shown. Overall a more rigorous analysis and presentation of the data would be important to make these conclusions convincing. This may involve refining the data presentation in the figures, modifying the claims (e.g., "we propose" vs "we find"), or saving some of the data to be more fully explored in a future paper. In my view, these figures are the biggest weak point of the manuscript and also are not important for the central conclusions (which are well supported and convincing), indeed these results are barely mentioned in the Abstract or last paragraph of Introduction.

- In Fig. 4, where in the head are we looking? It would help to show a more low-magnification view of the entire cell.
- In Fig. 4D, why is SAX-2 visible throughout the entire neuron and why is the "punctum" marked with an arrow also seen in the tagRFP channel? One gets the impression that some of the puncta may be background, bleed-through, or artifacts due to cell varicosities.
- In Fig. 4C, the distinction between puncta in the primary or higher-order dendrites is not clear to me, and several puncta that I would have scored as primary are marked as higher-order.
- Related to this, in Fig. 4B are the two arrows meant to be white as in the top panel, or yellow as in the bottom panel?
- The main sax-1 phenotype is increased SAX-2 puncta in dauer, but the branch retraction defect is in post-dauers. How is this relevant to the phenotype?
- The number of SAX-2 puncta in sax-1 mutants decreases almost to normal in post dauers. Is there a correlation between the number of remaining branches and the number of SAX-2 puncta? That is, do the many wild-type animals with "excess" SAX-2 puncta also fail to retract branches?
- The control post-dauer data in Fig. 4F and 4H are identical (re-used data) but the corresponding control dauer data in Fig. 4F and 4G are different. What is going on here?
- Why are sample sizes so small for both strains in Fig. 4G compared to Fig. 4F and 4H?
- In Fig. 6C, why are the tagRFP (blue) puncta larger than the neurite? Aren't these meant to represent vesicles inside the surrounding neurite? One gets the impression that this is bleed-through from the GFP channel.
- In Fig. 6E and 6F, why are there no tagRFP (blue) puncta? Is CD8 not endocytosed at all if it lacks the nanobody sequence? One would expect the tagRFP (blue) signal to be the same in both strains and simply to lack yellow if the nanobody is not present.
- In Fig. 6E and 6H, why are there so many GFP (yellow) puncta outside the neuron? What are these structures and why are they absent in the strain with the nanobody?
- What is the large central blue structure in Fig. 6H - is this the soma? - and why are puncta in this region not counted?
- The authors report a decrease in endocytic events in sax-1, but qualitatively it looks like there are vastly more puncta inside the neuron in Fig. 6H than in 6G.
- minor: there is text reading "40-" in the bottom panel of Fig. 6H. It is visible when printed but not on screen - adjust levels in Photoshop to reveal it.
- Related to both Fig. 4 and Fig. 6, where does SAX-1 localize in IL2Q in dauer and post-dauer? Does its expression or localization change during branch retraction? Does it co-localize with SAX-2 or endocytic puncta?

Minor points:

1. At several points the authors emphasize the relationship of neurite remodeling to stress, e.g.

Abstract and Discussion: "we adapted C. elegans IL2 sensory dendrites as a model [of...] stress-mediated dendrite pruning". It seems unnecessary and potentially misleading to treat this as a neuronal stress response. First, it conflates organismal and cellular stress - there is no reason to think that IL2 neurons are under cellular stress in dauer. In fact parasitic nematodes go through dauer-like stages as part of healthy development and probably have similar remodeling of IL2. Second, dendrite pruning occurs during dauer exit, which is the opposite of a stress response - it reflects a return to favorable conditions.
2. In Fig. 1A, C. elegans is shown going directly from L1 to dauer in response to unfavorable conditions, which is incorrect. Animals proceed through L2 (in many cases actually an alternative L2d pre-dauer) and then molt into dauer (an alternative L3 stage) after completing L2.
3. In Fig. 1B, please check if it is correct that hypodermis contacts the pharynx basement membrane as drawn. The schematic in the top panel makes it look like there is a single secondary branch and the quartenary branches are similar in length to the primary dendrite. The schematic in the bottom panel makes it look like the entire neuron is a small fraction of the length of the pharynx. Could these be drawn closer to scale?

Referee cross-commenting

I think we all touched on similar points. I wanted to follow up on Reviewer 3's comment, "Is the failure to eliminate branches an indication of incomplete dauer recovery? Do sax-1 mutants retain additional characteristics of dauer morphology in post dauer adults." I thought this was an excellent point. It made me wonder if that might explain why the defect is only seen in daf-7 and daf-2 mutant backgrounds - maybe these strains retain partial dauer traits even after exit. Is there a specific experiment that they could do? Did you have specific characteristics of dauer morphology in mind for them to check? (Ideally something in the nervous system that can be scored quantitatively.)

2. Significance:

Significance (Required)

A major strength of this work is the pioneering use of a novel system to study neuronal branch retraction. C. elegans has provided a powerful model for studying how dendrite branches form, but much less attention has been paid to how excess neuronal branches are removed. The post-dauer remodeling of IL2Q neurons provides an exciting and dramatic physiological example to explore this question.

This paper is notable for taking the first steps towards developing this innovative model. It does exactly what is needed at the outset of a new exploration - a forward genetic screen to discover the main regulators of the process. Using a combination of classical and modern genetic approaches, the authors bootstrap their way to a sizeable list of factors and a solid understanding of the properties of this system, for example that retraction of higher vs lower order dendrites show different genetic requirements.

3. How much time do you estimate the authors will need to complete the suggested revisions:

Estimated time to Complete Revisions (Required)

(Decision Recommendation)

Between 3 and 6 months

4. *Review Commons* values the work of reviewers and encourages them to get credit for their work. Select 'Yes' below to register your reviewing activity at Web of Science Reviewer Recognition Service (formerly Publons); note that the content of your review will not be visible on Web of Science.

Web of Science Reviewer Recognition

No

Review #2

1. Evidence, reproducibility and clarity:

Evidence, reproducibility and clarity (Required)

In this manuscript, the authors establish C. elegans IL2 neurons as a system in which to study dendrite pruning. They use the system to perform a genetic screen for pruning regulators and find an allele of sax-1. Unexpectedly sax-1 is only required for post-dauer pruning in two different genetic backgrounds that induce dauer formation, but not starvation-induced dauer formation. Sax-1/NDR kinase reduction has previously been associated with increased outgrowth and branching in other systems, so this is a new role for this protein. However, the authors show that proteins that work with Sax-1 in other systems, like sax-2/fry, also play a role in this pathway. The genetic experiments are beautiful and the findings are all clearly explained and strongly supported. The authors also examine sax-2 localization, which localizes sax-1 in other systems, and show it in puncta in dendrites that increase with dauer exit, consistent with function at the time of pruning. They also show that membrane trafficking regulators associated with NDR kinases function in the same pathway here, hinting that endocytosis may play a role during pruning as in Drosophila. The link to endocytosis was a little weak (see Major point below).

Overall, this study describes a new system to study pruning and identifies NDR/fry/Rabs as regulators of pruning during dauer exit. The work is very high quality and both the imaging and genetics are extremely well done.

Major points

1. The only place where there were any questions about the data was the last figure (6G and I). Here they use uptake of GFP secreted from muscle as a readout of endocytosis in IL2 neurons. They nicely show that more internalized puncta accumulate as animals exit dauer. The claim that this is reduced in sax-1 mutants doesn't seem to match the images shown well. In the image there are many more puncta in the GFP channel and much more accumulation of the RFP-tagged receptor everywhere. It seems like some additional analysis of this data is important to fully capture what is going on and whether this really represents an endocytic defect.
2. For context, it might be helpful to know whether branching of other dendrites is increased in sax-1 mutants (as expected based on phenotypes in other animals) or decreased like IL@ neurons.

Minor:

"shy87 mutant dauers showed a minor reduction in secondary and tertiary branches compared to control (Figure 1G). These results indicate that shy87 is specifically required for the elimination of dauer-generated dendrite branches." Maybe temper the specificity claim some as the reduction in branches is definitely there.

"three complimentary approaches" should be complementary

"In control animals, SAX-2 was mostly concentrated in the cell body (data not shown)" It might be nice to include some overview images that show the cell body for completeness.

2. Significance:

Significance (Required)

Neurite pruning is important in all animals with neurons. Genetic approaches have primarily been applied to the problem using Drosophila, so identifying a new model system in which to study it is an important step. Using this system, a pathway known to function in a different context is linked to pruning. Thus the study provides new insights into both pruning and this pathway.

3. How much time do you estimate the authors will need to complete the suggested revisions:

Estimated time to Complete Revisions (Required)

(Decision Recommendation)

Between 1 and 3 months

4. *Review Commons* values the work of reviewers and encourages them to get credit for their work. Select 'Yes' below to register your reviewing activity at Web of Science Reviewer Recognition Service (formerly Publons); note that the content of your review will not be visible on Web of Science.

Web of Science Reviewer Recognition

Yes

Review #3

1. Evidence, reproducibility and clarity:

Evidence, reproducibility and clarity (Required)

Summary:

Figueroa-Delgado et al. use a C. elegans neuro plasticity model to examine how dendrites are eliminated upon recovery from the stress induced larval stage, dauer. The authors performed a mutagenesis screen to identify novel regulators of dendrite elimination and revealed some surprising results. Branch elimination mechanism varies between 2{degree sign}, 3{degree sign}, and 4{degree sign} branches. The NDR kinase, SAX-1 and it's interactors (SAX-2 and MOB-2) are required for elimination of second and third order branches but not fourth order branches. Interestingly they showed that branch elimination varies depending on the stimulus of dendrite outgrowth such that the NDR kinase is required for branch elimination after genetically inducing the dauer stage but is not required if dauers are produced through food deprivation. The authors go a step further to include a small candidate screen looking at various pathways of membrane remodeling and identify additional regulators of dendrite elimination related to membrane trafficking including RABI-1, RAB-8, RAB-10, and RAB-11.2.

Major comments:

- While I find the data promising and exciting, several of the experiments have concerningly low sample sizes. Fig 3G, Fig 4G, Fig 5J and L, and Fig 6I all contain data sets that are fewer than 10

animals. Sample sizes should be stated specifically in the figure legends for all data represented in the graphs.
- All statements based on data not shown should be amended to include the data as a supplemental figure or edited to omit the statement based on withheld data.
- Rescue experiments (Fig 2J) should demonstrate failure to rescue from neighboring tissue types (hypodermis and muscle) to conclude cell autonomous rescue rather than a broadly acting factor.
- Fig 4 needs quantification of higher order branches and SAX-2 proximity to branch nodes as these are discussed in the text.

Minor comments:

- Fig 1C-F, It appears like the shy87 allele produces animals of significantly different body sizes. It would improve rigor to normalize the dendrite coverage to body size in the quantification.
- Fig 1G-H, are shy87 second and third order branch counts statistically different between dauer and post dauer adults? This comparison would strengthen the claim that these order branches fail to eliminate all together rather than undergo a partial elimination.
- Discussion of Fig 1G from the text claims that shy87 is specifically required for branch elimination yet the data shows significant defects in branch outgrowth as well. This raises the question, are the branches abnormally stabilized that results in early underdevelopment and late atrophy? Authors should acknowledge alternative hypotheses.
- Is the failure to eliminate branches an indication of incomplete dauer recovery? Do sax-1 mutants retain additional characteristics of dauer morphology in post dauer adults.
- The text references multiple transgenic lines tested in Fig 2I-J but only one line is shown.
- Fig 4B-E Indicate branch order in the images, this is unclear and a point that is focused on in the text.
- Fig 4F, Additional timepoints would enhance the sax-1 localization result and might provide insight into mechanism of action for sax-1.
- Authors reference a branch elimination process but don't outline what this would entail and where their results fit in.
- Fig 6I Control and sax-1(ky491) example images should be provided in the supplement.

Referee cross-commenting

I agree that we shared many of the same concerns.

There are several general assays for dauer characteristics that could be used here to determine if the post-dauer animals retain other characteristics of the dauer stage in addition to IL2 branches (SDS resistance, alae remodeling, pharyngeal bulb morphology, nictation behavior). The nictation behavior has been connected very nicely with IL2 neurons (Junho Lee's group). Additionally, FLP dendrites occupy the same space as the IL2 branches and outgrowth in post-dauers occurs in coordination with IL2 branch elimination - this might be another optional experiment, to check if FLP growth is impeded by persistent IL2 branches. All of these could be quantified similar to how the authors have already established with their IL2 model (FLP dendrite branches) or with a binary statistic.

2. Significance:

Significance (Required)

These results describe a new role for the NDR kinase complex in dendrite pruning that has clinical significance to our understanding of human brain development and human health concerns in which pruning is dysregulated, such as observed in the case of autism. The authors use an established neuro-plasticity, C. elegans model (Schroeder et al. 2013) which provides a tractable and reproduceable platform for discovering the mechanism of dendrite pruning. These results would influence future work in the fields of cell biology of the neuron and disease models of brain development.

My expertise is in the field of C. elegans neuroscience and stress biology and have sufficient expertise to evaluate all aspects of this work.

3. How much time do you estimate the authors will need to complete the suggested revisions:

Estimated time to Complete Revisions (Required)

(Decision Recommendation)

Between 1 and 3 months

4. *Review Commons* values the work of reviewers and encourages them to get credit for their work. Select 'Yes' below to register your reviewing activity at Web of Science Reviewer Recognition Service (formerly Publons); note that the content of your review will not be visible on Web of Science.

Web of Science Reviewer Recognition

Yes

Revision Plan

Manuscript number: RC- 2025-03073
Corresponding author(s): Shaul Yogev

1. General Statements [optional]

We kindly thank our reviewers for their enthusiasm, thoughtful feedback, and constructive suggestions on how to strengthen our manuscript. Below, we provide a point-by-point response to reviewer comments and outline the experiments we will do to address every concern that has been raised.

2. Description of the planned revisions

Reviewer #1 (Evidence, reproducibility and clarity (Required)):

This interesting study uses an unbiased genetic screen in C. elegans to identify SAX-1/NDR kinase as a regulator of dendritic branch elimination. Loss of SAX-1 results in an excess branching phenotype that is striking and highly penetrant. The authors identify several additional regulators of branch elimination (SAX-2, MOB-1, RABI-1, RAB-11.2) by using a candidate genetic screen aimed at factors that interact physically or genetically with SAX-1. They propose that SAX-1 acts by promoting membrane retrieval based on the nature of these interactors and the results of an imaging-based in vivo assay for endocytic puncta.

Major comments.

1. My biggest concern is that the phenotypes are only observed in temperature-sensitive dauer-constitutive mutant backgrounds, and not in wild-type dauers. That is, wild-type animals exiting dauer do not require SAX-1 for dendrite elimination. While this does not undermine the importance of the results, it does require more explanation. The authors write that "the requirement for sax-1... relies on specific physiological states of the dauer stage," but I do not understand what this means. Are they saying that daf-7 and daf-2 dauers are in a different "physiological state" than wild-type dauers? In what way? What is the evidence for this? A more rigorous explanation is needed.

We agree that this is puzzling, and we thank the reviewer for recognizing that this does not undermine the importance of the results. There is ample evidence that *daf-2* and *daf-7* differ from starvation-induced dauers. For example, a recent preprint finds that the transcriptomes of these two mutants at dauer cluster much closer to each other than to starvation-induced dauers (Corchado et al. 2024). Older work has noted other differences, such as the time the dauer entry decision is made (Swanson and Riddle 1981), the synchronicity of dauer exit, the ability to force

dauer entry in *daf-d* mutants, as well as additional dauer-unrelated phenotypes (reviewed in Karp 2018). We agree with the reviewer that this merits further clarifications and will perform the experiments suggested by the reviewer below:

To me, the simplest genetic explanation is that daf-7 and daf-2 are partially required for branch retraction in a manner redundant with sax-1, and the ts mutants are not fully wild-type at 15C. Thus, the sax-1 requirement is revealed only in these mutant backgrounds. Can the authors examine starvation-induced dauers of daf-7 or daf-2 raised continuously at 15C?

We will do this experiment.

daf-7 and daf-2 ts strains can form "partial dauers" that have a dauer-like appearance but are not SDS resistant. Could the difference between partial dauers and full dauers account for the difference in sax-1-dependence? The authors could use SDS selection of the daf-7 strain at 25C to ensure they are examining full dauers.

We tested *daf-7* mutants with 1% SDS when we set up the system – they are fully dauer at 25°C and are SDS sensitive after exit. We will repeat this important control with *daf-7; sax-1* double mutants.

The Bargmann lab has created a daf-2 FLP-OUT strain (ky1095ky1087) that allows cell-type-specific removal of daf-2. Could this be used to test for a cell-autonomous role of daf-2 in IL2Q related to branch elimination?

We can attempt this experiment. However, since IL2 promoters turn on prior to dauer, the interpretation would not be straightforward – it would be hard to exclude that a cell autonomous defect in dauer entry does not account for the IL2 dauer exit phenotype, even if branching appears normal.

These ideas are not a list of specific experiments the authors need to complete, rather they are meant to illustrate some possible approaches to the question. Whatever approach they use, it is important for them to more rigorously explain why SAX-1 is not required for branch removal in wild-type animals.

We completely agree. We will carry out the 15°C experiment, examine morphological characteristics and test SDS resistance. In addition, we will test neuronal markers that differ between dauers and non-dauers to determine whether the mutants are full or partial dauers at the relevant timepoints.

2. The SAX-2 localization (Fig. 4) and endocytosis assay (Fig. 6) results were not clear to me from the data shown. Overall a more rigorous analysis and presentation of the data would be important to make these conclusions convincing. This may involve refining the data presentation in the figures, modifying the claims (e.g., "we propose" vs "we find"), or saving some of the data

to be more fully explored in a future paper. In my view, these figures are the biggest weak point of the manuscript and also are not important for the central conclusions (which are well supported and convincing), indeed these results are barely mentioned in the Abstract or last paragraph of Introduction.

We agree that the analysis and presentation of Figures 4 and 6 need to be improved. The presentation has already been updated, and the figures are clearer now. In the revision, we will increase sample size to provide stronger conclusions, consolidate some of the analysis and further improve presentation. While we agree with the reviewer that conclusions from these figures are not as strong as those drawn from genetic experiments, they do complement and support the conclusions of those other figures.

- In Fig. 4D, why is SAX-2 visible throughout the entire neuron and why is the "punctum" marked with an arrow also seen in the tagRFP channel? One gets the impression that some of the puncta may be background, bleed-through, or artifacts due to cell varicosities.

There is no bleed-through: this is most evident by looking at the brightest signals in the cell body (now labelled with an asterisk in a zoomed-out image) and noting that they do not bleed between channels. In *sax-1* mutants, the SAX-2::GFP puncta are very obvious and distinguishable from the tagRFP channel. In control, SAX-2::GFP is very faint in the dendrite, so we increased the contrast to allow visualization. The reviewer is correct that under these conditions, some puncta look like the cytosolic fill. In the revision, we will re-analyze the data and will not consider these as bona-fide SAX-2 puncta, but rather cytosolic SAX-2 that accumulates due to constrictions and varicosities in the dendrite.

- Related to both Fig. 4 and Fig. 6, where does SAX-1 localize in IL2Q in dauer and post-dauer? Does its expression or localization change during branch retraction? Does it co-localize with SAX-2 or endocytic puncta?

We generated an endogenously tagged *sax-1* with a 7xspGFP11 tag; however, this was below detection in the IL2s. For the revisions, we can test an overexpressed cDNA construct.

Referee cross-commenting

I think we all touched on similar points. I wanted to follow up on Reviewer 3's comment, "Is the failure to eliminate branches an indication of incomplete dauer recovery? Do sax-1 mutants retain additional characteristics of dauer morphology in post dauer adults." I thought this was an excellent point. It made me wonder if that might explain why the defect is only seen in daf-7 and daf-2 mutant backgrounds - maybe these strains retain partial dauer traits even after exit. Is there a specific experiment that they could do? Did you have specific characteristics of dauer morphology in mind for them to check? (Ideally something in the nervous system that can be scored quantitatively.)

Please see response to point #1 regarding experiments we will do to confirm the “dauer state” of *daf-7* and *daf-7; sax-1* double mutants.

Reviewer #1 (Significance (Required)):

A major strength of this work is the pioneering use of a novel system to study neuronal branch retraction. C. elegans has provided a powerful model for studying how dendrite branches form, but much less attention has been paid to how excess neuronal branches are removed. The post-dauer remodeling of IL2Q neurons provides an exciting and dramatic physiological example to explore this question.

This paper is notable for taking the first steps towards developing this innovative model. It does exactly what is needed at the outset of a new exploration - a forward genetic screen to discover the main regulators of the process. Using a combination of classical and modern genetic approaches, the authors bootstrap their way to a sizeable list of factors and a solid understanding of the properties of this system, for example that retraction of higher vs lower order dendrites show different genetic requirements.

We thank the reviewer for recognizing the novelty and significance of our work.

Reviewer #2 (Evidence, reproducibility and clarity (Required)):

In this manuscript, the authors establish C. elegans IL2 neurons as a system in which to study dendrite pruning. They use the system to perform a genetic screen for pruning regulators and find an allele of sax-1. Unexpectedly sax-1 is only required for post-dauer pruning in two different genetic backgrounds that induce dauer formation, but not starvation-induced dauer formation. Sax-1/NDR kinase reduction has previously been associated with increased outgrowth and branching in other systems, so this is a new role for this protein. However, the authors show that proteins that work with Sax-1 in other systems, like sax-2/fry, also play a role in this pathway. The genetic experiments are beautiful and the findings are all clearly explained and strongly supported. The authors also examine sax-2 localization, which localizes sax-1 in other systems, and show it in puncta in dendrites that increase with dauer exit, consistent with function at the time of pruning. They also show that membrane trafficking regulators associated with NDR kinases function in the same pathway here, hinting that endocytosis may play a role during pruning as in Drosophila. The link to endocytosis was a little weak (see Major point below). Overall, this study describes a new system to study pruning and identifies NDR/fry/Rabs as regulators of pruning during dauer exit. The work is very high quality and both the imaging and genetics are extremely well done.

We thank the reviewer for their positive assessment of the manuscript.

Major points

1. The only place where there were any questions about the data was the last figure (6G and I). Here they use uptake of GFP secreted from muscle as a readout of endocytosis in IL2 neurons. They nicely show that more internalized puncta accumulate as animals exit dauer. The claim that this is reduced in sax-1 mutants doesn't seem to match the images shown well. In the image there are many more puncta in the GFP channel and much more accumulation of the RFP-tagged receptor everywhere. It seems like some additional analysis of this data is important to fully capture what is going on and whether this really represents an endocytic defect.

We agree and will provide additional data in Figure 6. The specific discrepancy between the image and the quantification is because we showed a single focal plane rather than a projection. This does not capture all the puncta in a neurite. The current version shows a projection, making it evident that the mutants has fewer puncta compared to the control.

Reviewer #2 (Significance (Required)):

Neurite pruning is important in all animals with neurons. Genetic approaches have primarily been applied to the problem using Drosophila, so identifying a new model system in which to study it is an important step. Using this system, a pathway known to function in a different context is linked to pruning. Thus the study provides new insights into both pruning and this pathway.

We thank the reviewer for the positive assessment of our study’s significance.

Reviewer #3 (Evidence, reproducibility and clarity (Required)):

Summary:
Figueroa-Delgado et al. use a C. elegans neuro plasticity model to examine how dendrites are eliminated upon recovery from the stress induced larval stage, dauer. The authors performed a mutagenesis screen to identify novel regulators of dendrite elimination and revealed some surprising results. Branch elimination mechanism varies between 2{degree sign}, 3{degree sign}, and 4{degree sign} branches. The NDR kinase, SAX-1 and it's interactors (SAX-2 and MOB-2) are required for elimination of second and third order branches but not fourth order branches. Interestingly they showed that branch elimination varies depending on the stimulus of dendrite outgrowth such that the NDR kinase is required for branch elimination after genetically inducing the dauer stage but is not required if dauers are produced through food deprivation. The authors go a step further to include a small candidate screen looking at various pathways of membrane remodeling and identify additional regulators of dendrite elimination related to membrane trafficking including RABI-1, RAB-8, RAB-10, and RAB-11.2.

We thank the reviewer for their time and suggestions below

Major comments:

Review COMMONS

• While I find the data promising and exciting, several of the experiments have concerningly low sample sizes. Fig 3G, Fig 4G, Fig 5J and L, and Fig 6I all contain data sets that are fewer than 10 animals. Sample sizes should be stated specifically in the figure legends for all data represented in the graphs.

We thank the reviewer for finding the data exciting. We agree that the sample sizes in some panels is low and will increase it in the revised version. Sample sizes are now specifically listed in the figure legends.

• All statements based on data not shown should be amended to include the data as a supplemental figure or edited to omit the statement based on withheld data.

We agree. Some “not shown” data are already added to the current version of the manuscript and the rest will be added to the fully revised version, or the statements will be omitted.

• Rescue experiments (Fig 2J) should demonstrate failure to rescue from neighboring tissue types (hypodermis and muscle) to conclude cell autonomous rescue rather than a broadly acting factor.

Thank you for the suggestion. We will use a hypodermal promoter and a muscle promoter driving SAX-1 cDNA expression to strengthen the claim of cell autonomy.

• Fig 4 needs quantification of higher order branches and SAX-2 proximity to branch nodes as these are discussed in the text.

We will add this quantification.

Minor comments:

• Fig 1C-F, It appears like the shy87 allele produces animals of significantly different body sizes. It would improve rigor to normalize the dendrite coverage to body size in the quantification.

We do not see a biologically meaningful size difference between *shy87* and control, it may be the specific image shown. We will confirm this by measuring animal size for the final revision.

• Is the failure to eliminate branches an indication of incomplete dauer recovery? Do sax-1 mutants retain additional characteristics of dauer morphology in post dauer adults.

This important point was also raised by Reviewer 1. We will test SDS sensitivity, morphological markers, and molecular markers to determine the dauer “state” of the mutants used in this study. The results will be included in the final revision.

• The text references multiple transgenic lines tested in Fig 2I-J but only one line is shown.

Additional lines were visually examined under a fluorescent compound microscope but not imaged or quantified. We will add this quantification to the final revision.

• Fig 4F, Additional timepoints would enhance the sax-1 localization result and might provide insight into mechanism of action for sax-1.

We will add the localization in post-dauer adults.

• Fig 6I Control and sax-1(ky491) example images should be provided in the supplement.

We will add these images to the final revision.

Referee cross-commenting

I agree that we shared many of the same concerns.

There are several general assays for dauer characteristics that could be used here to determine if the post-dauer animals retain other characteristics of the dauer stage in addition to IL2 branches (SDS resistance, alae remodeling, pharyngeal bulb morphology, nictation behavior). The nictation behavior has been connected very nicely with IL2 neurons (Junho Lee's group). Additionally, FLP dendrites occupy the same space as the IL2 branches and outgrowth in post-dauers occurs in coordination with IL2 branch elimination - this might be another optional experiment, to check if FLP growth is impeded by persistent IL2 branches. All of these could be quantified similar to how the authors have already established with their IL2 model (FLP dendrite branches) or with a binary statistic.

Please see responses to Reviewer 1 and 3 above for the list of experiments to determine whether the animals fail to completely enter or exit dauer.

Reviewer #3 (Significance (Required)):

SIGNIFICANCE
============
These results describe a new role for the NDR kinase complex in dendrite pruning that has clinical significance to our understanding of human brain development and human health concerns in which pruning is dysregulated, such as observed in the case of autism. The authors use an established neuro-plasticity, C. elegans model (Schroeder et al. 2013) which provides a tractable and reproduceable platform for discovering the mechanism of dendrite pruning. These results would influence future work in the fields of cell biology of the neuron and disease models of brain development.

My expertise is in the field of C. elegans neuroscience and stress biology and have sufficient expertise to evaluate all aspects of this work.

3. Description of the revisions that have already been incorporated in the transferred manuscript

Reviewer #1

- In Fig. 4C, the distinction between puncta in the primary or higher-order dendrites is not clear to me, and several puncta that I would have scored as primary are marked as higher-order.

We apologize for a mistake in the arrowhead color and overall presentation of this figure. It has been fixed in the current version.

- Related to this, in Fig. 4B are the two arrows meant to be white as in the top panel, or yellow as in the bottom panel?

We thank Reviewer #1 for their observation, and we apologize for our oversight. We fixed this in the current version.

- In Fig. 4, where in the head are we looking? It would help to show a more low-magnification view of the entire cell.

We added zoomed-out images and indicated where the zoomed in insets are taken from. We thank the reviewer for helping us improve the clarity of the data.

- The main sax-1 phenotype is increased SAX-2 puncta in dauer, but the branch retraction defect is in post-dauers. How is this relevant to the phenotype?

This is a very good point. The increase in SAX-2 puncta in *sax-1* mutants is stronger during dauer-exit than in dauer, consistent with this being the time when SAX-1 functions. We agree that some earlier activity of SAX-1 cannot be excluded, and we do not assume that the effect on SAX-2 completely accounts for the pruning defects. This is now acknowledged in the text. However, given that both proteins function together in pruning, and given that the effect is strongest during dauer exit, we do believe that this data is informative and worth showing.

- The number of SAX-2 puncta in sax-1 mutants decreases almost to normal in post dauers. Is there a correlation between the number of remaining branches and the number of SAX-2 puncta? That is, do the many wild-type animals with "excess" SAX-2 puncta also fail to retract branches?

There is no correlation. In other words, the number of SAX-2 puncta does not instruct the extent of pruning. Please note the quantifications underestimate the number of SAX-2 puncta in the mutants, since they were only done on the primary dendrite. This is necessary because the mutant and control have different arbor size, so only branch order that can be appropriately compared are primary dendrites.

- The control post-dauer data in Fig. 4F and 4H are identical (re-used data) but the corresponding control dauer data in Fig. 4F and 4G are different. What is going on here?

We thank the reviewer for raising this point and apologize for the oversight in data presentation. In the revised manuscript, we now show all control and experimental data integrated into a single graph, ensuring that each dataset is represented accurately to provide a comparison between dauer and post dauer recovery conditions.

- Why are sample sizes so small for both strains in Fig. 4G compared to Fig. 4F and 4H?

We sincerely apologize for this mistake, some of the data was erroneously grouped in the original submission. The revised version contains an updated number of neurons, presented on the same graph, and in the final revision we will further increase sample size. We apologize again for this error.

- In Fig. 6C, why are the tagRFP (blue) puncta larger than the neurite? Aren't these meant to represent vesicles inside the surrounding neurite? One gets the impression that this is bleed-through from the GFP channel.

Based on EM, both an endocytic punctum and the diameter of the neuron are smaller than a single pixel. The apparent difference in size in fluorescence microscopy is because the puncta are brighter (they contain more membrane) and thus appear larger. In the current version, the improved presentation of the figure contains zoomed out images that clearly show that there is no bleed-through.

- In Fig. 6E and 6F, why are there no tagRFP (blue) puncta? Is CD8 not endocytosed at all if it lacks the nanobody sequence? One would expect the tagRFP (blue) signal to be the same in both strains and simply to lack yellow if the nanobody is not present.

CD8 lacks clear endocytosis motifs, which is why it is advantageous for labelling neurites and testing endocytosis when paired with an endocytic signal (Lee and Luo 1999; Kozik et al. 2010). Conversely, extracellular GFP binding to a membrane GFP antibody can induce endocytosis (for example, see (Tang et al., 2020)), likely by inducing clustering, although we are not familiar with work that explored the mechanism. In the updated version we included a rare example of an mCD8 punctum.

- The authors report a decrease in endocytic events in sax-1, but qualitatively it looks like there are vastly more puncta inside the neuron in Fig. 6H than in 6G.

We apologize for the presentation in the original version of Figure 6. This impression was because we showed single focal planes that only captured some of the signal. In the revised version we show projections, which makes it evident that there are fewer endocytic events in the mutant.

- In Fig. 6E and 6H, why are there so many GFP (yellow) puncta outside the neuron? What are these structures and why are they absent in the strain with the nanobody?

These puncta are secreted or muscle-associated GFP that has not been internalized by IL2Q neurons. They are present in all strains in this figure, this can be clearly seen in the zoomed-out images that have been added to the updated figure.

- What is the large central blue structure in Fig. 6H - is this the soma? - and why are puncta in this region not counted?

This is indeed the soma. In the updated version this can be clearly seen in the zoom-out. The large puncta in the soma were not counted because they may arise from the fusion of an unknown number of smaller puncta, and their precise number cannot be determined at the resolution of fluorescence microscopy.

- minor: there is text reading "40-" in the bottom panel of Fig. 6H. It is visible when printed but not on screen - adjust levels in Photoshop to reveal it.

We thank the reviewer for catching this oversight, it is now fixed.

Minor points:

1. At several points the authors emphasize the relationship of neurite remodeling to stress, e.g. Abstract and Discussion: "we adapted C. elegans IL2 sensory dendrites as a model [of...] stress-mediated dendrite pruning". It seems unnecessary and potentially misleading to treat this as a neuronal stress response. First, it conflates organismal and cellular stress - there is no reason to think that IL2 neurons are under cellular stress in dauer. In fact parasitic nematodes go through dauer-like stages as part of healthy development and probably have similar remodeling of IL2. Second, dendrite pruning occurs during dauer exit, which is the opposite of a stress response - it reflects a return to favorable conditions.

We agree. We modified the abstract and discussion to avoid conflating organismal stress (the alleviation of which is relevant for triggering pruning) and cellular stress. Thank you for pointing this out.

2. In Fig. 1A, C. elegans is shown going directly from L1 to dauer in response to unfavorable

conditions, which is incorrect. Animals proceed through L2 (in many cases actually an alternative L2d pre-dauer) and then molt into dauer (an alternative L3 stage) after completing L2.

We updated the schematic to include the L2d stage where commitment to dauer entry or resumption to reproductive development is made.

3. In Fig. 1B, please check if it is correct that hypodermis contacts the pharynx basement membrane as drawn. The schematic in the top panel makes it look like there is a single secondary branch and the quaternary branches are similar in length to the primary dendrite. The schematic in the bottom panel makes it look like the entire neuron is a small fraction of the length of the pharynx. Could these be drawn closer to scale?

The hypodermis does contact the pharynx basement membrane. We redrew the schematic for clarity.

Reviewer #2

2. For context, it might be helpful to know whether branching of other dendrites is increased in sax-1 mutants (as expected based on phenotypes in other animals) or decreased like IL2 neurons.

We examined the branching pattern of PVD, a polymodal nociceptive neuron (new Supplemental Figure 3). We find no significant difference between control and *sax-1* or *sax-2* mutants, suggesting that these genes function in the context of pruning. Recent work (Zhao et al. 2022) confirms that *sax-1* is not required for PVD branching.

Minor:

"shy87 mutant dauers showed a minor reduction in secondary and tertiary branches compared to control (Figure 1G). These results indicate that shy87 is specifically required for the elimination of dauer-generated dendrite branches." Maybe temper the specificity claim some as the reduction in branches is definitely there.

We agree, the claim was tempered.

"three complimentary approaches" should be complementary

Thank you for noticing. We fixed this.

"In control animals, SAX-2 was mostly concentrated in the cell body (data not shown)" It might be nice to include some overview images that show the cell body for completeness.

We added zoomed-out images to the revised figure, thank you for the suggestion.

Reviewer #3

Minor comments:

• Fig 1G-H, are shy87 second and third order branch counts statistically different between dauer and post dauer adults? This comparison would strengthen the claim that these order branches fail to eliminate all together rather than undergo a partial elimination.

We added this to Figure S2. The *shy87* mutants show a complete failure in eliminating secondary branches (i.e. no difference between dauer and post-dauer) and a strong but incomplete defect in eliminating tertiary branches.

• Fig 4B-E Indicate branch order in the images, this is unclear and a point that is focused on in the text.

Done.

• Discussion of Fig 1G from the text claims that shy87 is specifically required for branch elimination yet the data shows significant defects in branch outgrowth as well. This raises the question, are the branches abnormally stabilized that results in early underdevelopment and late atrophy? Authors should acknowledge alternative hypotheses.

We agree and will revise the text accordingly. The difference between *shy87* and control dauers, while statistically significant, is relatively minor and can only be detected by careful quantification, it is not apparent from looking at the images (in contrast for example to *rab-8* and *rab-10* mutants, where we acknowledge in the text that their branching defects might affect subsequent pruning.

• Authors reference a branch elimination process but don't outline what this would entail and where their results fit in.

We apologize for being unclear. Given that *sax-1* and *sax-2* function together, one would intuitively expect to see SAX-2 being reduced in *sax-1* mutants, yet the opposite is observed. On potential explanation is that SAX-1 does not directly control SAX-2 abundance, but that clearance of SAX-2 is part of the pruning process that both proteins regulate. This would explain the enrichment of SAX-2 in *sax-1* mutants. However, additional models cannot be excluded, and we acknowledge this in the revised text.

References:

Corchado, Johnny Cruz, Abhishiktha Godthi, Kavinila Selvarasu, and Veena Prahlad. 2024. “Robustness and Variability in Caenorhabditis Elegans Dauer Gene Expression.” Preprint, bioRxiv, August 26. https://doi.org/10.1101/2024.08.15.608164.

Karp, Xantha. 2018. “Working with Dauer Larvae.” *WormBook*, August 9, 1–19. https://doi.org/10.1895/wormbook.1.180.1.

Kozik, Patrycja, Richard W Francis, Matthew N J Seaman, and Margaret S Robinson. 2010. “A Screen for Endocytic Motifs.” *Traffic (Copenhagen, Denmark)* 11 (6): 843–55. https://doi.org/10.1111/j.1600-0854.2010.01056.x.

Lee, T., and L. Luo. 1999. “Mosaic Analysis with a Repressible Cell Marker for Studies of Gene Function in Neuronal Morphogenesis.” *Neuron* 22 (3): 451–61.

Swanson, M. M., and D. L. Riddle. 1981. “Critical Periods in the Development of the Caenorhabditis Elegans Dauer Larva.” *Developmental Biology* 84 (1): 27–40. https://doi.org/10.1016/0012-1606(81)90367-5.

Tang, Rui, Christopher W Murray, Ian L Linde, et al. n.d. “A Versatile System to Record Cell-Cell Interactions.” *eLife* 9: e61080. https://doi.org/10.7554/eLife.61080.

Zhao, Ting, Liying Guan, Xuehua Ma, Baohui Chen, Mei Ding, and Wei Zou. 2022. “The Cell Cortex-Localized Protein CHDP-1 Is Required for Dendritic Development and Transport in C. Elegans Neurons.” *PLOS Genetics* 18 (9): e1010381. https://doi.org/10.1371/journal.pgen.1010381.

4. Description of analyses that authors prefer not to carry out

Dear Dr. Yogev,

Thank you for submitting your manuscript for consideration by the EMBO Journal. I apologise for the delay in the assessment of your submission due to the unusually high manuscript submission rate to our office at the moment.

I have now read your manuscript, the reviewer comments and your response to them. Based on our editorial assessment and the referees' positive evaluations, I would like to invite you to submit a revised version of the manuscript along the lines indicated in your revision plan.

We generally allow three months as standard revision time. Should you foresee a problem in meeting this three-month deadline, please let us know in advance in order to arrange an extension.

As a matter of policy, competing manuscripts published during this period will not negatively impact on our assessment of the conceptual advance presented by your study. However, please contact me as soon as possible upon publication of any related work to discuss the appropriate course of action.

When preparing your letter of response to the referees' comments, please bear in mind that this will form part of the Review Process File and will therefore be available online to the community. For more details on our Transparent Editorial Process, please visit our website: https://www.embopress.org/page/journal/14602075/authorguide#transparentprocess. Please also see the attached instructions for further guidelines on preparation of the revised manuscript.

Please feel free to contact me if you have any further questions regarding the revision. Thank you for the opportunity to consider your work for publication. I look forward to receiving your revised manuscript.

With best wishes,

Ieva

Ieva Gailite, PhD
Senior Scientific Editor
The EMBO Journal
Meyerhofstrasse 1
D-69117 Heidelberg
Tel: +4962218891309
i.gailite@embojournal.org

Instructions for preparing your revised manuscript:

Please make sure you upload a letter of response to the referees' comments together with the revised manuscript.

Please also check that the title and abstract of the manuscript are brief, yet explicit, even to non-specialists.

When assembling figures, please refer to our figure preparation guideline in order to ensure proper formatting and readability in print as well as on screen:
https://bit.ly/EMBOPressFigurePreparationGuideline
See also figure legend guidelines: https://www.embopress.org/page/journal/14602075/authorguide#figureformat

At EMBO Press we ask authors to provide source data for the main manuscript figures. You will receive a separate email with instructions for providing source data with your revised manuscript, including how to upload and organize the files.

IMPORTANT: When you send the revision we will require
- a point-by-point response to the referees' comments, with a detailed description of the changes made (as a word file).
- a word file of the manuscript text.
- individual production quality figure files (one file per figure)
- a complete author checklist, which you can download from our author guidelines (https://www.embopress.org/page/journal/14602075/authorguide).
- Expanded View files (replacing Supplementary Information)
Please see out instructions to authors

https://www.embopress.org/page/journal/14602075/authorguide#expandedview
- a Reagents and Tools Table as part of the Methods section, which can be downloaded from our author guidelines (https://www.embopress.org/page/journal/14602075/authorguide#structuredmethods)

Please remember: Digital image enhancement is acceptable practice, as long as it accurately represents the original data and conforms to community standards. If a figure has been subjected to significant electronic manipulation, this must be noted in the figure legend or in the 'Materials and Methods' section. The editors reserve the right to request original versions of figures and the original images that were used to assemble the figure.

Further information is available in our Guide For Authors: https://www.embopress.org/page/journal/14602075/authorguide

We realize that it is difficult to revise to a specific deadline. In the interest of protecting the conceptual advance provided by the work, we recommend a revision within 3 months (30th Nov 2025). Please discuss the revision progress ahead of this time with the editor if you require more time to complete the revisions. Use the link below to submit your revision:

Link Not Available

***

Rev_Com_number: RC-2025-03073
New_manu_number: EMBOJ-2025-122251-T
Corr_author: Yogev
Title: NDR kinase SAX-1 controls dendrite branch-specific elimination during neuronal remodeling in C. elegans

Dear editor, reviewers,

Thank you for the opportunity to revise our manuscript. We kindly thank our reviewers for their enthusiasm, thoughtful feedback, and constructive suggestions on how to strengthen our manuscript.

We have carefully considered all reviewer comments and concerns and have carried out additional experiments to address the concerns. We apologize for the lengthy revision process, it is a result of working with post dauers: we raise and cross all animals at 15C, and each experiment requires inducing dauer, waiting a week, and inducing dauer exit. We sincerely appreciate the editor's and the reviewers' patience.

Below, we provide a point-by-point response to reviewer comments and the corresponding changes implemented in the revised manuscript.

Reviewer #1 (Evidence, reproducibility and clarity (Required)):

This interesting study uses an unbiased genetic screen in C. elegans to identify SAX-1/NDR kinase as a regulator of dendritic branch elimination. Loss of SAX-1 results in an excess branching phenotype that is striking and highly penetrant. The authors identify several additional regulators of branch elimination (SAX-2, MOB-1, RABI-1, RAB-11.2) by using a candidate genetic screen aimed at factors that interact physically or genetically with SAX-1. They propose that SAX-1 acts by promoting membrane retrieval based on the nature of these interactors and the results of an imaging-based in vivo assay for endocytic puncta.

Major comments.

1. My biggest concern is that the phenotypes are only observed in temperature-sensitive dauer-constitutive mutant backgrounds, and not in wild-type dauers. That is, wild-type animals exiting dauer do not require SAX-1 for dendrite elimination.

While this does not undermine the importance of the results, it does require more explanation. The authors write that "the requirement for sax-1... relies on specific physiological states of the dauer stage," but I do not understand what this means. Are they saying that daf-7 and daf-2 dauers are in a different "physiological state" than wild-type dauers? In what way? What is the evidence for this? A more rigorous explanation is needed.

We agree that this is puzzling, and we thank the reviewer for recognizing that this does not undermine the importance of the results. There is ample evidence that *daf-2* and *daf-7* dauers differ from stress-induced dauers. For example, a recent preprint finds that the transcriptomes of these two mutants at dauer cluster much closer to each other than to temperature-induced N2 dauers (Corchado et al. 2024). Older work has noted other differences, such as the time the dauer entry decision is made (Swanson and Riddle 1981), the synchronicity of dauer exit, the ability to force dauer entry in *daf-c* mutants, as well as additional dauer-unrelated phenotypes (reviewed in Karp 2018). We agree with the reviewer that this merits further clarifications. In addition to mentioning the above references in the manuscript, we have performed the following experiments suggested by the reviewer below:

To me, the simplest genetic explanation is that daf-7 and daf-2 are partially required for branch retraction in a manner redundant with sax-1, and the ts mutants are not fully wild-type at 15C.

Thus, the sax-1 requirement is revealed only in these mutant backgrounds. Can the authors examine starvation-induced dauers of daf-7 or daf-2 raised continuously at 15C?

We examined *daf-7* mutants continuously raised at 15C, starved to form dauers, and subsequently treated with 1% SDS for dauer selection. *sax-1(shy87)* did not fully eliminate the dauer-born arbor, as shown here.

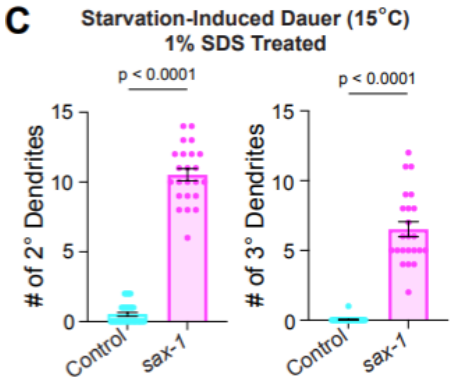

Unfortunately, the interpretation of this result is tricky, since even at 15C, these ts mutants are very different from N2. For example, some enter dauer even at 15C, and at least some of those that do not are believed to go through L2d. Dauer exit of *daf-7 (e1372 ts)* mutants is also different from exit of N2 strains – it is slower and asynchronous. These differences and other are summarized in the excellent dauer chapter of WormBook. Hence, this experiment may support a direct role in pruning or an indirect role in dauer. Since *daf-2*, which has a very different biological role from *daf-7* – except that both are *daf-c* – is also required for *sax-1* pruning defects, we believe that the most parsimonious explanation is that dauers generated by both mutants differ from N2 dauers by some characteristic that is important for SAX-1-dependent pruning. In the revision, we speculate that this difference may be in expression of cell surface molecules, given that their expression is different in the different dauers, and that their endocytosis is important for pruning (Zhang et al. 2014; Corchado et al. 2024). Because of the difficulty of interpreting this experiment, we show it here to the reviewers but prefer to leave it out of the manuscript.

daf-7 and daf-2 ts strains can form "partial dauers" that have a dauer-like appearance but are not SDS resistant. Could the difference between partial dauers and full dauers account for the difference in sax-1-dependence? The authors could use SDS selection of the daf-7 strain at 25C to ensure they are examining full dauers.

We tested *daf-7* mutants and *daf-7; sax-1* double mutants with SDS. They are fully dauer (SDS-resistant) at 25°C and fully SDS-sensitive after dauer exit (**Supplemental Figure 4**). These results indicate that the requirement for *daf-7* is not due to it forming partial dauers.

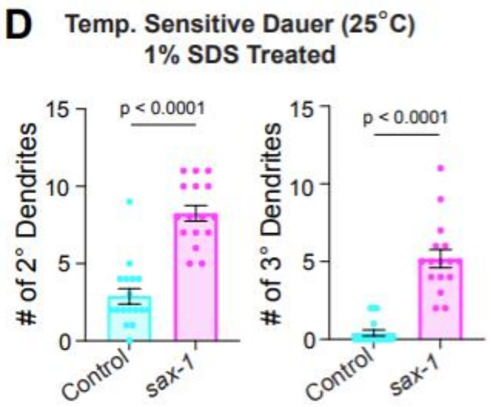

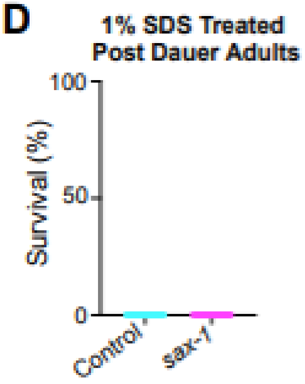

The Bargmann lab has created a daf-2 FLP-OUT strain (ky1095ky1087) that allows cell-type-specific removal of daf-2. Could this be used to test for a cell-autonomous role of daf-2 in IL2Q related to branch elimination? These ideas are not a list of specific experiments the authors need to complete, rather they are meant to illustrate some possible approaches to the question. Whatever approach they use, it is important for them to more rigorously explain why SAX-1 is not required for branch removal in wild-type animals.

We agree with the reviewer (and reviewer #3) that this needs further clarifications.

We could not get reliable FLPing-out with the *daf-2* allele from the Bargmann lab (note that in their hands the efficiency was also variable and their methods section describes an error in the design of the allele which makes it hard to determine if recombination occurred – the mCherry is out of frame). Troubleshooting this issue would have further extended the revisions. We are also not sure how this experiment would be interpreted, since IL2 promoters turn on before dauer any interpretation would have to take earlier functions into account.

We have taken various other steps to ensure that remnant branches in *daf-7; sax-1* double mutants are not because these mutants maintain the IL2s in a dauer-like state (this was the major concern arising from the discussion between Reviewer 1 and Reviewer 3). At the animal morphology level, we confirmed the lack of dauer characteristics after *daf-7; sax-1* double mutants are induced to exit dauer: (1) SDS resistance in dauer and sensitivity after exit, as described above. (2) lack of a buccal plug in post dauer adults (**Figure S4**). (3) Alae morphology at dauer and post dauer adults (**Figure S4**). (4) Pharynx morphology (**Figure S4**).

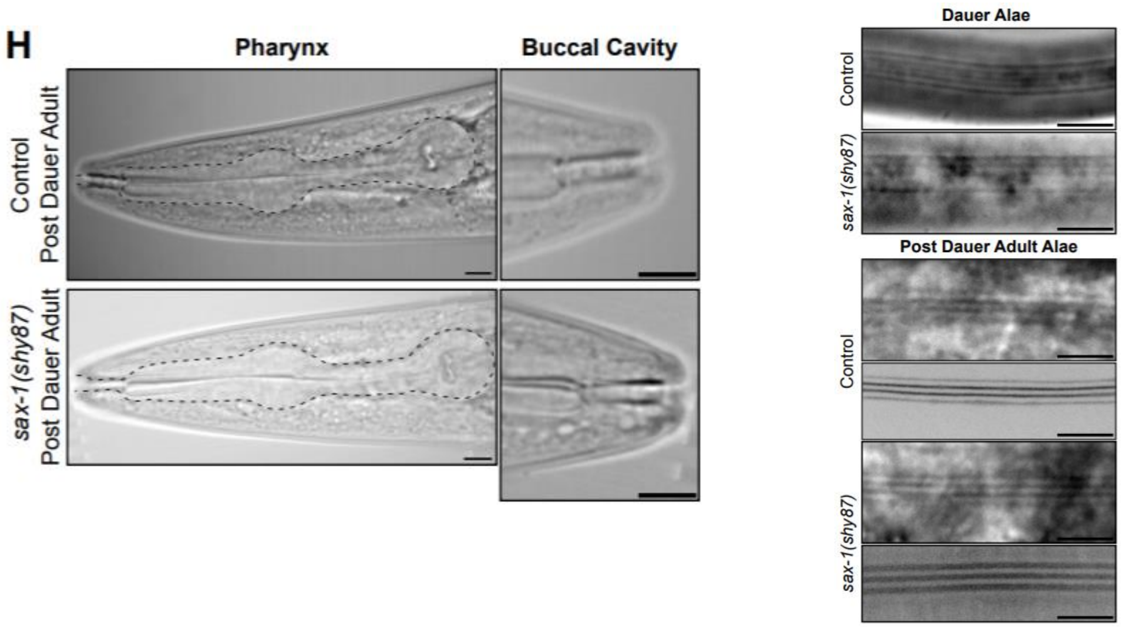

At a molecular level, we sought neuronal markers for the dauer state. We tested *sru*-12p::GFP, a neuronal GPCR transgenic strain that is reported to have decreased expression at dauer that is later recovered when the animal resumes reproductive development (Vidal et al. 2018). Counting cells expressing *sru*-12p::GFP in adults revealed no differences between *daf-7; sax-1* and control, suggesting that IL2s exited dauer. Note, however, that this array is expressed in nearby neurons that we could not always distinguish from the IL2s and were therefore included

in the count, leading to more *sru*-12p::GFP than 6. Therefore, we feel that this experiment – while supportive of our conclusions – is not sufficiently conclusive and so we only report it here, not in the manuscript.

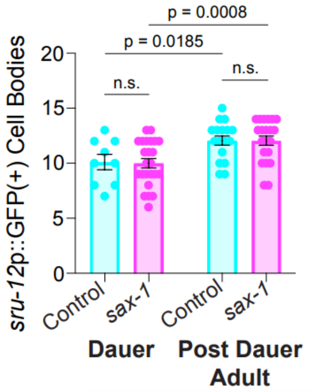

As a more robust readout for dauer exit at a single neuron level we examined PVD. Similar to the IL2s, it is a highly branched sensory neuron. PVD arrests dendrite growth in dauer and resumes it post-dauer (Richardson et al. 2019). We observe that post-dauer *daf-7; sax-1* mutants have similar dendritic arbors as control, indicating that PVD successfully exited dauer (**Figure S4**).

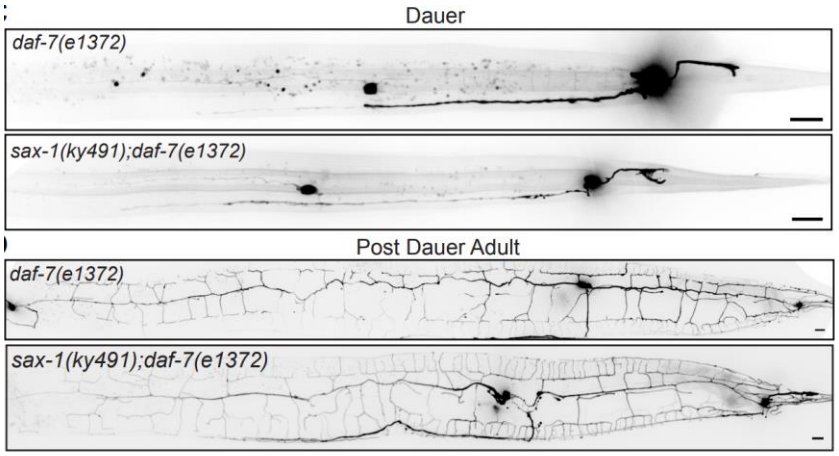

We conclude that *sax-1* loss does not leave *daf-7* mutant neurons in a dauer-like state, which in the case of IL2 would remain unbranched. Our best guess is that the differential requirement for *sax-1* might be due to a different repertoire of cell surface molecules, as these differ between N2 and *daf-2/7* dauers and play important roles in endocytosis-mediated pruning (Corchado et al. 2024; Zhang et al. 2014). This is added to the Discussion in the revised manuscript.
We thank the reviewer for pushing us to do these experiments, they have significantly strengthened the manuscript.

2. The SAX-2 localization (Fig. 4) and endocytosis assay (Fig. 6) results were not clear to me from the data shown. Overall a more rigorous analysis and presentation of the data would be important to make these conclusions convincing. This may involve refining the data presentation in the figures, modifying the claims (e.g., "we propose" vs "we find"), or saving some of the data to be more fully explored in a future paper. In my view, these figures are the biggest weak point

of the manuscript and also are not important for the central conclusions (which are well supported and convincing), indeed these results are barely mentioned in the Abstract or last paragraph of Introduction.

We agree that the analysis and presentation of Figures 4 and 6 needed to be improved. The presentation has been updated, and the figures are clearer now. We significantly increased sample size and consolidated some of the analyses to provide clearer conclusions. While we agree with the reviewer that conclusions from these figures are not as foundational to the overall model as the genetic results reported in other figures, we believe that they do complement and support the conclusions of these other figures.

- In Fig. 4, where in the head are we looking? It would help to show a more low-magnification view of the entire cell.
We added zoomed-out images and indicated where the zoomed in insets are taken from - see example below. We thank the reviewer for helping us improve the clarity of the data.

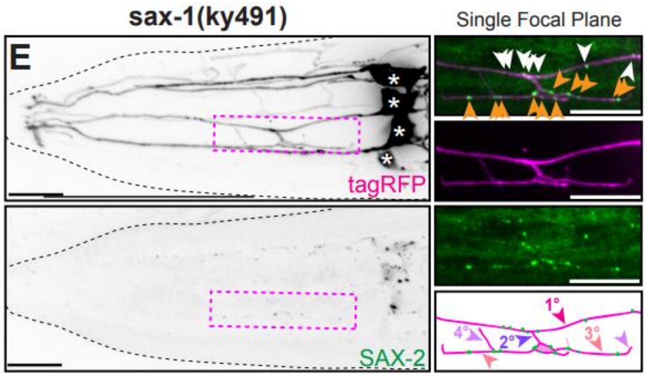

- In Fig. 4D, why is SAX-2 visible throughout the entire neuron and why is the "punctum" marked with an arrow also seen in the tagRFP channel? One gets the impression that some of the puncta may be background, bleed-through, or artifacts due to cell varicosities.
We do not detect significant bleed-through – many bright signals are associated with only one channel (also see example below). In some cases, particularly in controls (where SAX-2::GFP forms fewer puncta and is more diffuse), SAX-2::GFP is more visible in varicosities or branchpoints, which have a higher volume. We now highlight such an event in **Figure 4F** (and see below) where varicosity-localized SAX-2 is right next to a SAX-2 punctum that is not associated with stronger RFP signal. This also supports that there is no bleed-through. In *sax-1* mutants, the SAX-2::GFP puncta are very obvious and distinguishable from the RFP channel.

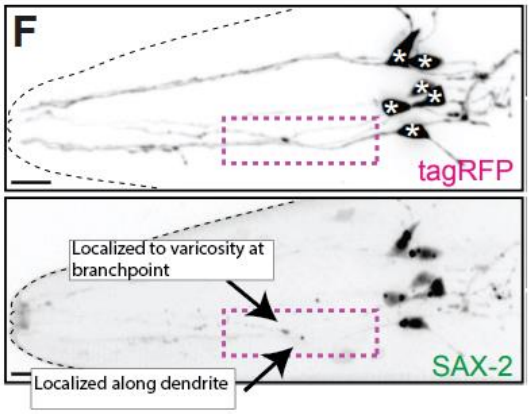

- In Fig. 4C, the distinction between puncta in the primary or higher-order dendrites is not clear to me, and several puncta that I would have scored as primary are marked as higher-order.

We apologize for a mistake in the arrowhead color and overall presentation of this figure. It has been fixed in the manuscript.

- Related to this, in Fig. 4B are the two arrows meant to be white as in the top panel, or yellow as in the bottom panel?
We thank the reviewer for their observation, and we apologize for our oversight. We fixed this in the revised manuscript.

The main sax-1 phenotype is increased SAX-2 puncta in dauer, but the branch retraction defect is in post-dauers. How is this relevant to the phenotype?
In the revised manuscript we added additional imaging time points, increased n, and also report on higher order branches. *sax-1* mutants consistently show more puncta than controls, with a peak between 16-20 Hrs post dauer. While this corresponds to a time when pruning occurs, the reviewer is correct that the difference is also observed at other timepoints. This suggests that SAX-2 puncta numbers are not instructive for pruning (see also next question), which we acknowledge in the revised Discussion. However, the fact that both proteins function together in pruning does suggest that this increase may be meaningful. Our hypothesis is that regulation of membrane dynamics by SAX-1 and SAX-2 is particularly important during pruning, but occurs at baseline levels across development and adulthood. This is also included in the Discussion.

- The number of SAX-2 puncta in sax-1 mutants decreases almost to normal in post dauers. Is there a correlation between the number of remaining branches and the number of SAX-2 puncta? That is, do the many wild-type animals with "excess" SAX-2 puncta also fail to retract branches?
There is no correlation. In other words, the number of SAX-2 puncta does not instruct the extent of pruning – this is included in the revised Discussion. Please note that the quantifications in the first submission underestimated the number of SAX-2 puncta in the mutants because we only showed 1ary dendrites (since higher order branches become reduced in number over time in the control). In the revised version we show both primary a higher order and normalize to branch length. This shows more clearly the extent of the phenotype, which extends into post dauers.

The control post-dauer data in Fig. 4F and 4H are identical (re-used data) but the corresponding control dauer data in Fig. 4F and 4G are different. What is going on here?
We thank the reviewer for spotting this point and apologize for the oversight. In the revised manuscript, we ensured that each dataset is represented accurately.

Why are sample sizes so small for both strains in Fig. 4G compared to Fig. 4F and 4H?
We sincerely apologize for this mistake, some of the data was erroneously grouped in the original submission. The revised version contains a significantly larger number of neurons, with correct grouping.

In Fig. 6C, why are the tagRFP (blue) puncta larger than the neurite? Aren't these meant to represent vesicles inside the surrounding neurite? One gets the impression that this is bleed-through from the GFP channel.
Based on EM, both an endocytic punctum and the diameter of the neuron are smaller than a single pixel. The apparent difference in size in fluorescence microscopy is because the puncta are brighter (they contain more membrane) and thus appear larger. In the current version, the improved presentation does not provide this impression and many pixels contain bright GFP signal but no RFP, clearly showing that there is no bleed-through.

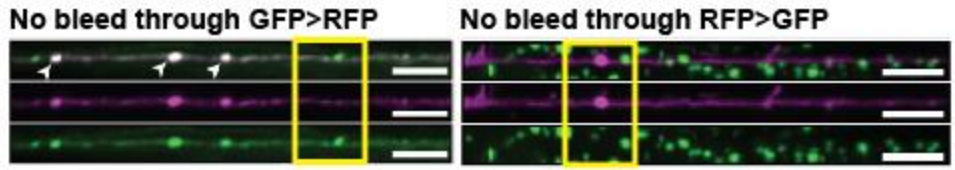

In Fig. 6E and 6F, why are there no tagRFP (blue) puncta? Is CD8 not endocytosed at all if it lacks the nanobody sequence? One would expect the tagRFP (blue) signal to be the same in both strains and simply to lack yellow if the nanobody is not present.

CD8 lacks clear endocytosis motifs, which is why it is advantageous for labelling neurites and testing endocytosis when paired with an endocytic signal (Lee and Luo 1999; Kozik et al. 2010). Conversely, extracellular GFP binding to a membrane GFP antibody can induce endocytosis (for example, see (Tang et al., 2020)), *likely* by inducing clustering, although we are not familiar with work that explored the mechanism. In the updated version we included a rare example of an mCD8 punctum (also shown below).

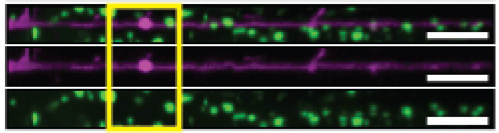

In Fig. 6E and 6H, why are there so many GFP (yellow) puncta outside the neuron? What are these structures and why are they absent in the strain with the nanobody?

These puncta are secreted or muscle-associated GFP that has not been internalized by IL2Q neurons. They are present in all strains in this figure, this can be better appreciated in the zoomed-out images that have been added to the updated figure (see example below). We apologize again for the poor presentation in the original version.

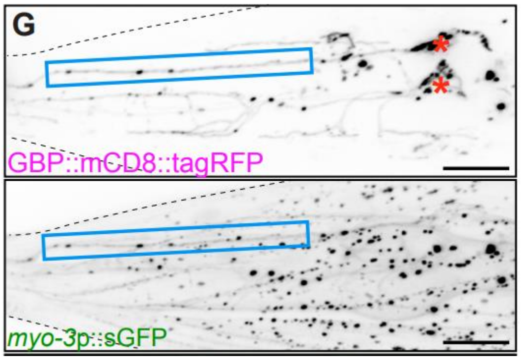

What is the large central blue structure in Fig. 6H - is this the soma? - and why are puncta in this region not counted?

Yes, the large central blue structures were indeed the soma. In the updated version, this can be appreciated in the zoomed-out projections (see example above). The large puncta in the soma were not counted as they may arise from the fusion of an unknown number of smaller puncta, and their precise number cannot be determined at the resolution of confocal microscopy.

The authors report a decrease in endocytic events in sax-1, but qualitatively it looks like there are vastly more puncta inside the neuron in Fig. 6H than in 6G.

The decrease in endocytic events is very evident in the quantifications (now with increased sample size) and we replaced the example image for better agreement with the quantifications.

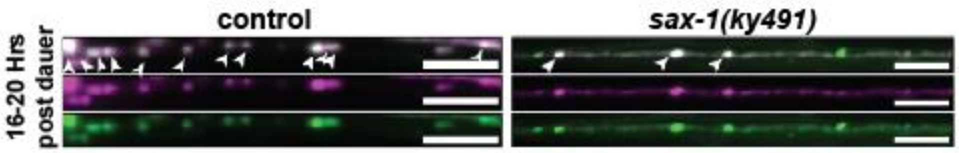

Minor: there is text reading "40-" in the bottom panel of Fig. 6H. It is visible when printed but not on screen - adjust levels in Photoshop to reveal it.
We thank the reviewer for catching this oversight, it is now fixed.

Related to both Fig. 4 and Fig. 6, where does SAX-1 localize in IL2Q in dauer and post-dauer? Does its expression or localization change during branch retraction? Does it co-localize with SAX-2 or endocytic puncta?
The Bargmann lab reported cytosolic expression of transgenic SAX-1::GFP (Zallen et al. 2000). We generated a 7xspGFP11 endo-tag but the signal was below detection. We used transgenic expression of a rescuing SAX-1::GFP and confirmed cytosolic expression. In the revised manuscript we report that we confirmed the result from the Bargmann lab. Unfortunately, this localization is not informative for understanding SAX-1 function in pruning.

Minor points:
1. At several points the authors emphasize the relationship of neurite remodeling to stress, e.g. Abstract and Discussion: "we adapted C. elegans IL2 sensory dendrites as a model [of...] stress-mediated dendrite pruning". It seems unnecessary and potentially misleading to treat this as a neuronal stress response. First, it conflates organismal and cellular stress - there is no reason to think that IL2 neurons are under cellular stress in dauer. In fact parasitic nematodes go through dauer-like stages as part of healthy development and probably have similar remodeling of IL2. Second, dendrite pruning occurs during dauer exit, which is the opposite of a stress response - it reflects a return to favorable conditions.
We agree. We modified the abstract and discussion to avoid conflating organismal stress (the alleviation of which is relevant for triggering pruning) and cellular stress. Thank you for pointing this out.

2. In Fig. 1A, C. elegans is shown going directly from L1 to dauer in response to unfavorable conditions, which is incorrect. Animals proceed through L2 (in many cases actually an alternative L2d pre-dauer) and then molt into dauer (an alternative L3 stage) after completing L2.
We updated the schematic to include the L2d stage where commitment to dauer entry or resumption to reproductive development is made.

3.In Fig. 1B, please check if it is correct that hypodermis contacts the pharynx basement membrane as drawn. The schematic in the top panel makes it look like there is a single secondary branch and the quartenary branches are similar in length to the primary dendrite. The schematic in the bottom panel makes it look like the entire neuron is a small fraction of the length of the pharynx. Could these be drawn closer to scale?
The hypodermis does contact the pharynx basement membrane. We redrew the schematic for clarity.

Referee cross-commenting

I think we all touched on similar points. I wanted to follow up on Reviewer 3's comment, "Is the failure to eliminate branches an indication of incomplete dauer recovery? Do sax-1 mutants retain additional characteristics of dauer morphology in post dauer adults." I thought this was an excellent point. It made me wonder if that might explain why the defect is only seen in daf-7 and daf-2 mutant backgrounds - maybe these strains retain partial dauer traits even after exit. Is there a specific experiment that they could do? Did you have specific characteristics of dauer morphology in mind for them to check? (Ideally something in the nervous system that can be scored quantitatively.)
We agree with both reviewers that this is an important point. Please see the various experiments we did to address it in point #1 above. Our experiments indicate that *sax-1* mutants do not retain observable dauer characteristics.

Reviewer #1 (Significance (Required)):

A major strength of this work is the pioneering use of a novel system to study neuronal branch retraction. C. elegans has provided a powerful model for studying how dendrite branches form, but much less attention has been paid to how excess neuronal branches are removed. The post-dauer remodeling of IL2Q neurons provides an exciting and dramatic physiological example to explore this question.

This paper is notable for taking the first steps towards developing this innovative model. It does exactly what is needed at the outset of a new exploration - a forward genetic screen to discover the main regulators of the process. Using a combination of classical and modern genetic approaches, the authors bootstrap their way to a sizeable list of factors and a solid understanding of the properties of this system, for example that retraction of higher vs lower order dendrites show different genetic requirements.

We thank the reviewer for recognizing the novelty and significance of our work and thank them again for their time and excellent comments.

Reviewer #2 (Evidence, reproducibility and clarity (Required)):

In this manuscript, the authors establish C. elegans IL2 neurons as a system in which to study dendrite pruning. They use the system to perform a genetic screen for pruning regulators and find an allele of sax-1. Unexpectedly sax-1 is only required for post-dauer pruning in two different genetic backgrounds that induce dauer formation, but not starvation-induced dauer formation. Sax-1/NDR kinase reduction has previously been associated with increased outgrowth and branching in other systems, so this is a new role for this protein. However, the authors show that proteins that work with Sax-1 in other systems, like sax-2/fry, also play a role in this pathway. The genetic experiments are beautiful and the findings are all clearly explained and strongly supported. The authors also examine sax-2 localization, which localizes sax-1 in other systems, and show it in puncta in dendrites that increase with dauer exit, consistent with function at the time of pruning. They also show that membrane trafficking regulators associated with NDR kinases function in the same pathway here, hinting that endocytosis may play a role during pruning as in Drosophila. The link to endocytosis was a little weak (see Major point below). Overall, this study describes a new system to study pruning and identifies NDR/fry/Rabs as regulators of pruning during dauer exit. The work is very high quality and both the imaging and genetics are extremely well done.

We thank the reviewer for their positive assessment of the manuscript.

1. The only place where there were any questions about the data was the last figure (6G and I). Here they use uptake of GFP secreted from muscle as a readout of endocytosis in IL2 neurons. They nicely show that more internalized puncta accumulate as animals exit dauer. The claim that this is reduced in sax-1 mutants doesn't seem to match the images shown well. In the image there are many more puncta in the GFP channel and much more accumulation of the RFP-tagged receptor everywhere. It seems like some additional analysis of this data is important to fully capture what is going on and whether this really represents an endocytic defect.

We apologize for the poor presentation of Figure 6 in the original version, which was also commented on by the other reviewers. The perceived inconsistency was because we only showed a single focal plane. The revised version contains zoomed-out projections along with zoom-ins. The n is also significantly increased. The data now clearly shows the phenotype of reduced endocytic events in the mutant.

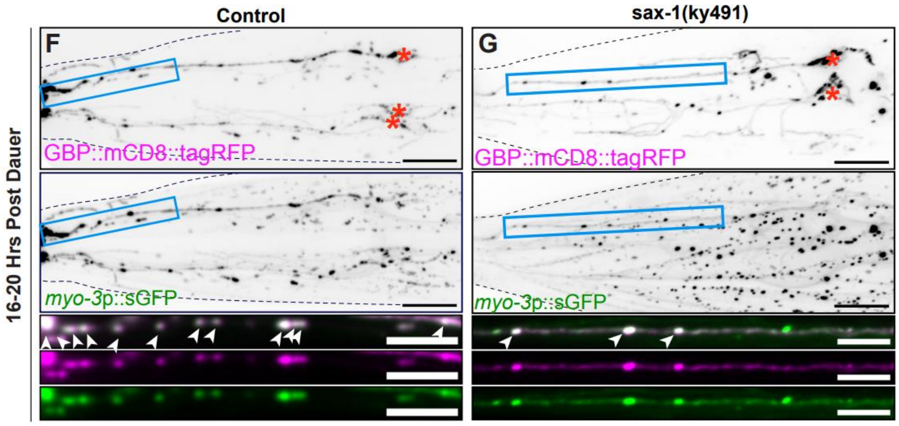

2. For context, it might be helpful to know whether branching of other dendrites is increased in sax-1 mutants (as expected based on phenotypes in other animals) or decreased like IL2 neurons.

We examined the branching pattern of PVD, a polymodal nociceptive neuron. PVD was recently reported to develop normally in *sax-1* mutants (Zhao et al. 2022). We confirmed this observation and tested *sax-2* mutants as well, which also showed normal development. This is added as **Figure S3**.

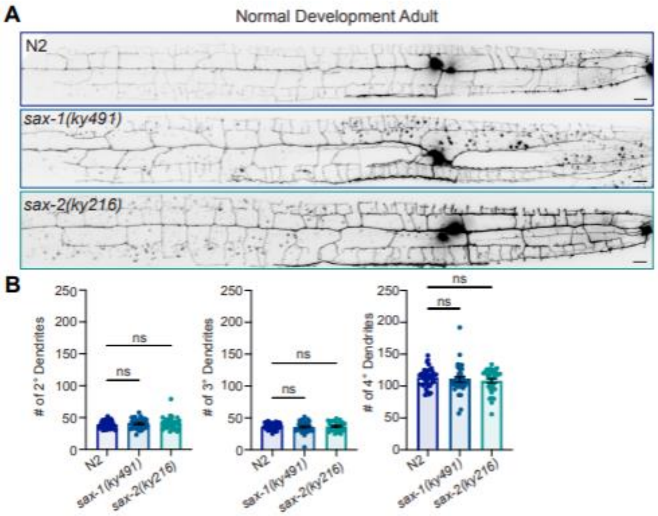

Minor:

"shy87 mutant dauers showed a minor reduction in secondary and tertiary branches compared to control (Figure 1G). These results indicate that shy87 is specifically required for the elimination of dauer-generated dendrite branches." Maybe temper the specificity claim some as the reduction in branches is definitely there.
We agree, the claim was tempered in the revised manuscript.

"three complimentary approaches" should be complementary
Thank you for noticing. We fixed this.

"In control animals, SAX-2 was mostly concentrated in the cell body (data not shown)" It might be nice to include some overview images that show the cell body for completeness.
Thank you for the suggestion. In the revised figure, we have included zoomed-out maximum projections that include the cell bodies.

Reviewer #2 (Significance (Required)):

Neurite pruning is important in all animals with neurons. Genetic approaches have primarily been applied to the problem using Drosophila, so identifying a new model system in which to study it is an important step. Using this system, a pathway known to function in a different context is linked to pruning. Thus the study provides new insights into both pruning and this pathway.

We thank the reviewer for the positive assessment of our study's significance.

Reviewer #3 (Evidence, reproducibility and clarity (Required)):

Summary:
Figueroa-Delgado et al. use a C. elegans neuro plasticity model to examine how dendrites are eliminated upon recovery from the stress induced larval stage, dauer. The authors performed a mutagenesis screen to identify novel regulators of dendrite elimination and revealed some surprising results. Branch elimination mechanism varies between 2{degree sign}, 3{degree sign}, and 4{degree sign} branches. The NDR kinase, SAX-1 and it's interactors (SAX-2 and

MOB-2) are required for elimination of second and third order branches but not fourth order branches. Interestingly they showed that branch elimination varies depending on the stimulus of dendrite outgrowth such that the NDR kinase is required for branch elimination after genetically inducing the dauer stage but is not required if dauers are produced through food deprivation. The authors go a step further to include a small candidate screen looking at various pathways of membrane remodeling and identify additional regulators of dendrite elimination related to membrane trafficking including RABI-1, RAB-8, RAB-10, and RAB-11.2.

We thank the reviewer for their time and suggestions below.

Major comments:

• While I find the data promising and exciting, several of the experiments have concerningly low sample sizes. Fig 3G, Fig 4G, Fig 5J and L, and Fig 6I all contain data sets that are fewer than 10 animals. Sample sizes should be stated specifically in the figure legends for all data represented in the graphs.
We agree with the reviewer. Sample size is increased in the revision across all figures and stated in the figure legends.

• All statements based on data not shown should be amended to include the data as a supplemental figure or edited to omit the statement based on withheld data.
We now show all the data that is described in the text.

• Rescue experiments (Fig 2J) should demonstrate failure to rescue from neighboring tissue types (hypodermis and muscle) to conclude cell autonomous rescue rather than a broadly acting factor.
In our revised manuscript, we ectopically expressed SAX-1 cDNA in the hypodermis and muscle, driven by *dpy-7* and *myo-3* promoter, respectively. We assessed multiple transgenic lines per construct and observed a failure to rescue (**Figure 2H-I;K**), concluding that SAX-1 acts cell autonomously.

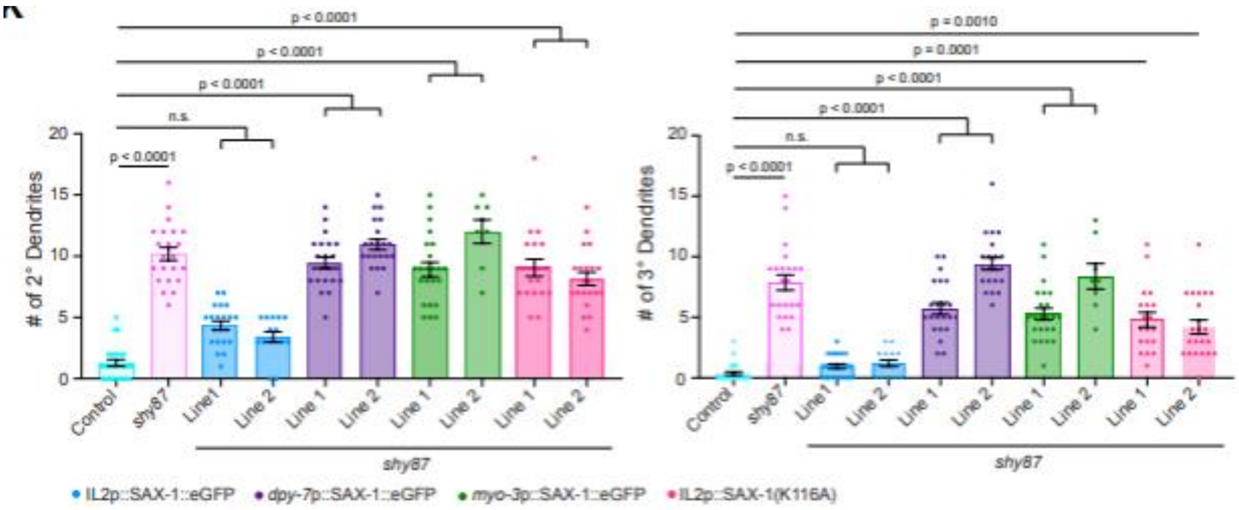

• Fig 4 needs quantification of higher order branches and SAX-2 proximity to branch nodes as these are discussed in the text.

We significantly changed the presentation of Figure 4, including higher n, additional timepoints and the requested quantification of SAX-2 in puncta in higher order branches (where we normalized by branch numbers to account for the pruning that takes place in control but not sax-1 mutants). We removed the claim about preferential localization to branchpoint, because as correctly argued by reviewer #1, we cannot distinguish at these sites a real punctum from accumulation of soluble SAX-2 in a varicosity.

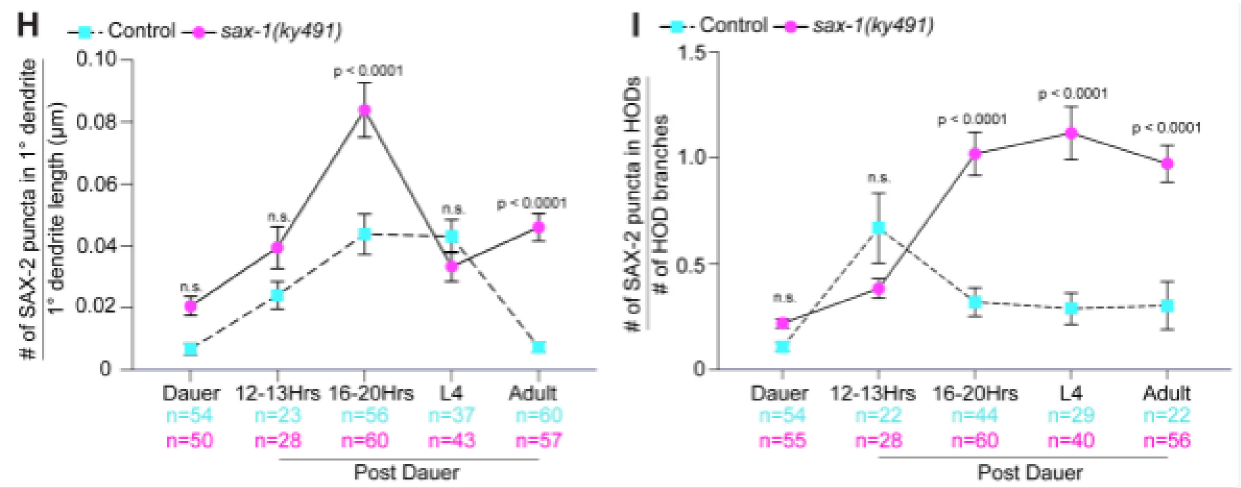

Minor comments:

• Fig 1C-F, It appears like the shy87 allele produces animals of significantly different body sizes. It would improve rigor to normalize the dendrite coverage to body size in the quantification.

We measured animal size carefully and it is not meaningfully changed in sax-1 mutants (**Figure S4**). Some of the representative images have been changed in the revision and we hope that this corrects the impression of a different body size.

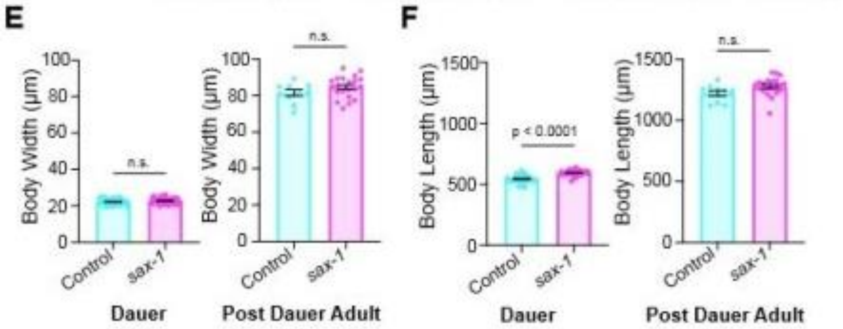

• Fig 1G-H, are shy87 second and third order branch counts statistically different between dauer and post dauer adults? This comparison would strengthen the claim that these order branches fail to eliminate all together rather than undergo a partial elimination.

We have added this data in **Figure S2** and added a "pruning index" to **Figure 1**. *shy87* mutants show a near-complete failure to eliminate secondary branches (although the difference with dauer shows statistical significance). The defect in tertiary branch elimination is strong but incomplete. Quaternary branches are nearly unaffected.

• Discussion of Fig 1G from the text claims that shy87 is specifically required for branch elimination yet the data shows significant defects in branch outgrowth as well. This raises the question, are the branches abnormally stabilized that results in early underdevelopment and late atrophy? Authors should acknowledge alternative hypotheses.

We revised the text to acknowledge this. We would like to emphasize that the difference between *shy87* and control dauers, while statistically significant, is quite minor (small effect size) and can only be detected by careful quantification, it is not apparent from looking at the images.

• Is the failure to eliminate branches an indication of incomplete dauer recovery? Do sax-1 mutants retain additional characteristics of dauer morphology in post dauer adults.
This question was a major issue for reviewer #1 and arose in the discussion between the reviewers. We therefore did extensive experiments to address it.

In short – we have looked at morphological criteria (SDS resistance, buccal plug, pharynx morphology, animal size and alae structure) and do not find evidence that the *sax-1* mutation causes *daf-7* mutants to retain dauer characteristics. We have also looked at neuronal markers – (1) a reporter from the Hobert lab the is meant to be expressed in post-dauer IL2 and (2) PVD, which stops dendrite growth in dauer and resumes it post-dauer. Again, we do not find evidence that *sax-1* affects dauer exit in neurons. Some of this data is shown in **Figure S4** and all of it is presented in the response to reviewer #1 (it is not copied here because reviewer 3 considered this a minor issue). We also discuss in the text a possible explanation for the differential requirement for *sax-1* between N2 and *daf-2/7*, citing a manuscript that details many transcriptional differences between N2 and *daf-2/7* dauers, including expression of cell surface molecules, which may account for this observation (Corchado et al. 2024; Zhang et al. 2014).

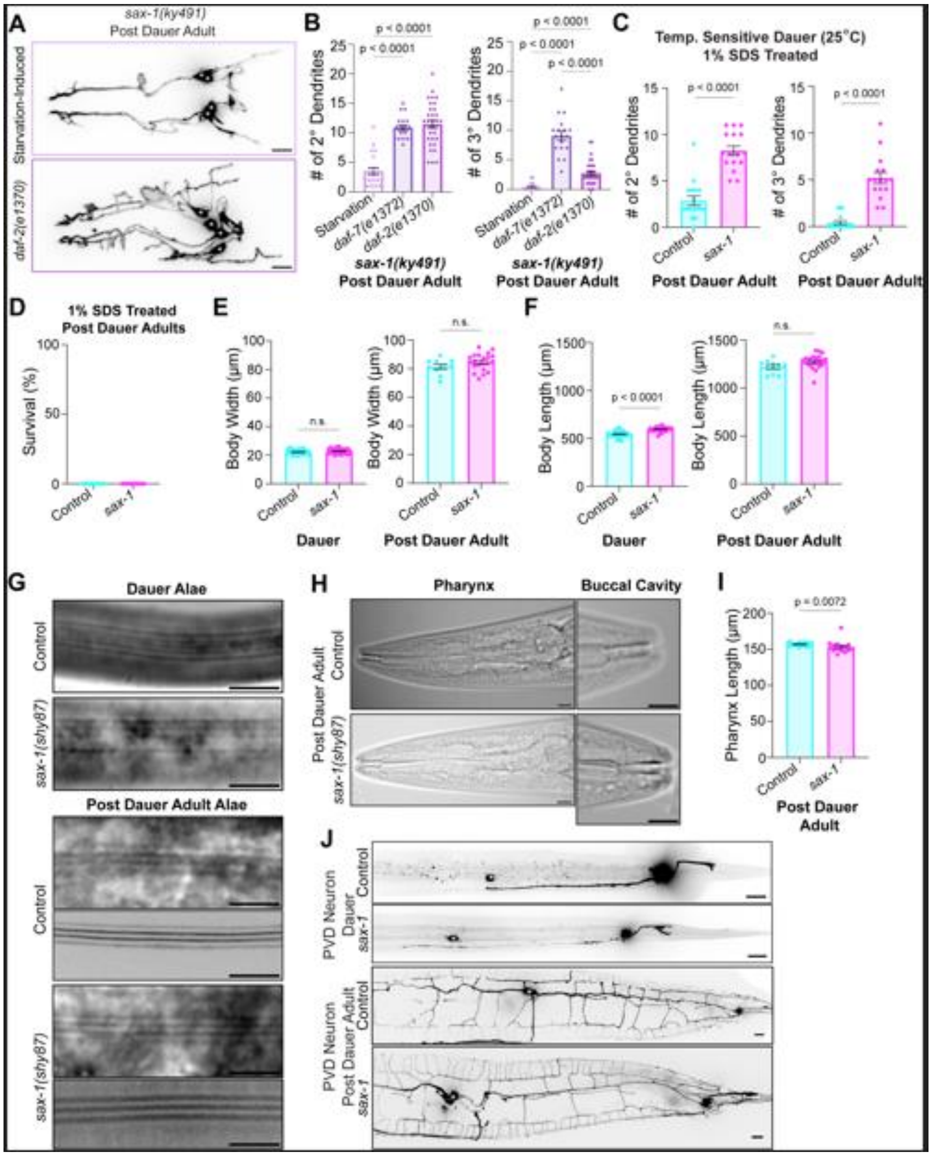

• The text references multiple transgenic lines tested in Fig 2I-J but only one line is shown.

We now show several transgenic lines, including for the failed rescue from hypodermis and muscle.

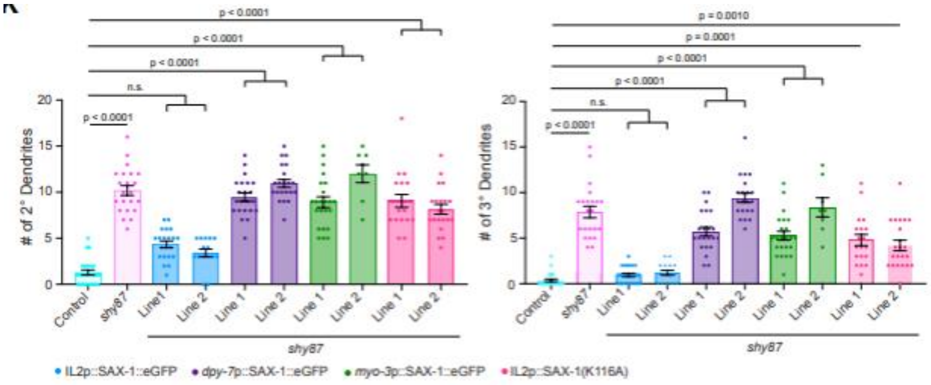

• Fig 4B-E Indicate branch order in the images, this is unclear and a point that is focused on in the text.
We have significantly overhauled Figure 4. The new version indicates branch order through schematics, see example below.

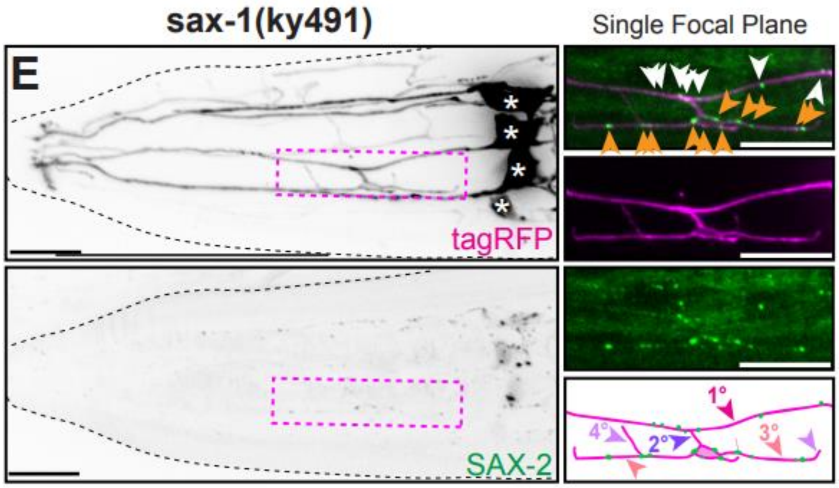

• Fig 4F, Additional timepoints would enhance the sax-1 localization result and might provide insight into mechanism of action for sax-1.
We added timepoints and included an analysis of SAX-2 in primary and higher order dendrites.

• Authors reference a branch elimination process but don't outline what this would entail and where their results fit in.
We apologize for being unclear. Given that *sax-1* and *sax-2* function together, one would intuitively expect SAX-2 to be reduced in *sax-1* mutants, yet the opposite is observed. One potential explanation is that SAX-1 does not directly control SAX-2 abundance, but that clearance of SAX-2 is part of the pruning process that both proteins regulate. This would explain the enrichment of SAX-2 in *sax-1* mutants. However, additional models cannot be excluded, and we acknowledge this in the revised text.

• Fig 6I Control and sax-1(ky491) example images should be provided in the supplement.
Figure 6 has been significantly changed (presentation, n, analysis) and the reported data is now shown in the figure. An example is shown below.

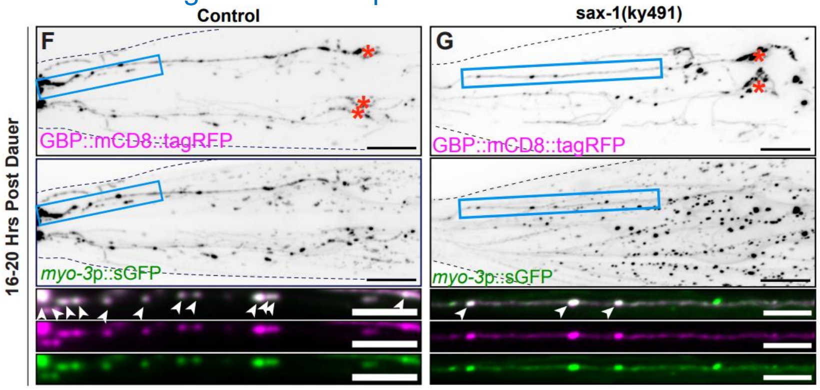

Referee cross-commenting

I agree that we shared many of the same concerns.
There are several general assays for dauer characteristics that could be used here to determine if the post-dauer animals retain other characteristics of the dauer stage in addition to IL2 branches (SDS resistance, alae remodeling, pharyngeal bulb morphology, nictation behavior). The nictation behavior has been connected very nicely with IL2 neurons (Junho Lee's group). Additionally, FLP dendrites occupy the same space as the IL2 branches and outgrowth in post-dauers occurs in coordination with IL2 branch elimination - this might be another optional experiment, to check if FLP growth is impeded by persistent IL2 branches. All of these could be quantified similar to how the authors have already established with their IL2 model (FLP dendrite branches) or with a binary statistic.
Please see our detailed response to reviewer #1 for the various assays we have used to test if *sax-1* mutants show dauer characteristics. Using both morphological criteria and neuronal markers we could not find evidence for such an effect.

Reviewer #3 (Significance (Required)):

These results describe a new role for the NDR kinase complex in dendrite pruning that has clinical significance to our understanding of human brain development and human health concerns in which pruning is dysregulated, such as observed in the case of autism. The authors use an established neuro-plasticity, C. elegans model (Schroeder et al. 2013) which provides a tractable and reproduceable platform for discovering the mechanism of dendrite pruning. These results would influence future work in the fields of cell biology of the neuron and disease models of brain development.

My expertise is in the field of C. elegans neuroscience and stress biology and have sufficient expertise to evaluate all aspects of this work.

We thank the reviewer for their time and for their helpful suggestions.

References:

Corchado, Johnny Cruz, Abhishiktha Godthi, Kavinila Selvarasu, and Veena Prahlad. 2024. “Robustness and Variability in Caenorhabditis Elegans Dauer Gene Expression.” Preprint, bioRxiv, August 26. https://doi.org/10.1101/2024.08.15.608164.

Karp, Xantha. 2018. “Working with Dauer Larvae.” *WormBook*, August 9, 1–19. https://doi.org/10.1895/wormbook.1.180.1.

Kozik, Patrycja, Richard W. Francis, Matthew N. J. Seaman, and Margaret S. Robinson. 2010. “A Screen for Endocytic Motifs.” *Traffic (Copenhagen, Denmark)* 11 (6): 843–55. https://doi.org/10.1111/j.1600-0854.2010.01056.x.

Lee, T., and L. Luo. 1999. “Mosaic Analysis with a Repressible Cell Marker for Studies of Gene Function in Neuronal Morphogenesis.” *Neuron* 22 (3): 451–61.

Richardson, Claire E., Callista Yee, and Kang Shen. 2019. “A Hormone Receptor Pathway Cell-Autonomously Delays Neuron Morphological Aging by Suppressing Endocytosis.” *PLoS Biology* 17 (10): e3000452. https://doi.org/10.1371/journal.pbio.3000452.

Swanson, M. M., and D. L. Riddle. 1981. “Critical Periods in the Development of the Caenorhabditis Elegans Dauer Larva.” *Developmental Biology* 84 (1): 27–40. https://doi.org/10.1016/0012-1606(81)90367-5.

Tang, Rui, Christopher W. Murray, Ian L. Linde, et al. n.d. “A Versatile System to Record Cell-Cell Interactions.” *eLife* 9: e61080. https://doi.org/10.7554/eLife.61080.

Vidal, Berta, Ulkar Aghayeva, Haosheng Sun, et al. 2018. “An Atlas of Caenorhabditis Elegans Chemoreceptor Expression.” *PLOS Biology* 16 (1): e2004218. https://doi.org/10.1371/journal.pbio.2004218.

Zallen, J. A., E. L. Peckol, D. M. Tobin, and C. I. Bargmann. 2000. “Neuronal Cell Shape and Neurite Initiation Are Regulated by the Ndr Kinase SAX-1, a Member of the Orb6/COT-1/Warts Serine/Threonine Kinase Family.” *Molecular Biology of the Cell* 11 (9): 3177–90. https://doi.org/10.1091/mbc.11.9.3177.

Zhang, Heng, Yan Wang, Jack Jing Lin Wong, et al. 2014. “Endocytic Pathways Downregulate the L1-Type Cell Adhesion Molecule Neuroglian to Promote Dendrite Pruning in Drosophila.” *Developmental Cell* 30 (4): 463–78. https://doi.org/10.1016/j.devcel.2014.06.014.

Zhao, Ting, Liying Guan, Xuehua Ma, Baohui Chen, Mei Ding, and Wei Zou. 2022. “The Cell Cortex-Localized Protein CHDP-1 Is Required for Dendritic Development and Transport in C. Elegans Neurons.” *PLOS Genetics* 18 (9): e1010381. https://doi.org/10.1371/journal.pgen.1010381.

1st Revision - Editorial Decision 5th May 2026

Dear Shaul,

Thank you for submitting a revised version of your manuscript. I sincerely apologise for the unusually prolonged assessment process due to delays in reviewer report submission and the high number of primary and revised submissions that we receive at the moment. We have now received input from two of the original reviewers. While reviewer #3 has not provided specific comments, they have indicated that they are satisfied with the final revision.

Therefore, there now remain only a few editorial and formatting points that need to be addressed before I can extend official acceptance of the manuscript:

1. Please note that it is The EMBO Journal policy for the transcript of the editorial process (containing referee reports and your response letters) to be published as an online supplement to each paper. If you should prefer removal of any referee-only figures included in the point-by-point response(s), e.g. because they may still be used for future publication or because they have been reproduced from published work by others, please do let us know immediately via response email.
More information is available here: https://www.embopress.org/transparent-process#Review_Process
2. Please check that the funding information is correct and identical both in the manuscript and our online system. Currently, NIH National Institute of General Medical Science (1S10OD030363-01A1); NIH Office of Research Infrastructure Programs (P40 OD010440); grant GE016776 to PVFD are not listed in the submission system.
3. The manuscript sections should be in the following order: Title page - Abstract - Introduction - Results - Discussion - Methods - Data Availability - Acknowledgments - Disclosure and Competing Interests Statement - References - Figure Legends - (Main Tables with legends if applicable) - Expanded View Figure Legends.
4. In the Author Checklist, please enter corresponding author's name, journal title and manuscript ID in the box on the top.
5. CRediT has replaced the traditional author contributions section because it offers a systematic, machine-readable author contributions format that allows for more effective research assessment. Please remove the Authors Contributions from the manuscript and use the free text boxes beneath each contributing author's name in our online submission system to add specific details on the author's contribution. More information is available in our guide to authors.
6. Please rename "Declaration of interests" section into "Disclosure and competing interests statement".
7. Please update references according to The EMBO Journal style - they should be listed alphabetically. Where there are more than 10 authors on a paper, the first 10 should be listed, followed by 'et al.' DOIs should only be used for preprints and datasets that have not been published yet.
8. Figure panels 2B, 3A,D, 5A,G are not mentioned in the manuscript text; please add the corresponding callouts.
9. Figure panel 4J is cited in the manuscript text but is not included in the figure.
10. There are references to Supplemental Figure 1 in the manuscript text, while such figure is not available. Please check and correct.
11. Please remove the Reagents and Tools Table from the manuscript text file and upload in our online system as a separate file choosing the file type "Reagent Table".
12. Please upload source data files as one zip folder per figure; source data should be clearly labelled according to the corresponding figure panel.
13. In our standard image check, we noticed potential image reuse between the following panels: Figure 1C (Control) and Figure EV1A (Control). If this was intentional, please indicate the reuse in the figure legends.
14. Our data editors have flagged the following issues in figure legends that need correcting:
- Please indicate the statistical test used for data analysis in the legends of figures 3G-I; EV4 I.
- Please provide information on the number and nature of replicates in the legends of figures 3G-I; 5J, K, M
- Please define the error bars in the legends of figures 1G, 5G, K, M; EV2 C, D; EV4 C, E, F, I.
- Please define the scale bar for figure EV4 J.
- Please define the asterisk in the legend of figure 1C, D; 2G-J; 6F, G.
15. Papers published in The EMBO Journal are accompanied online by a 'Synopsis' to enhance discoverability of the manuscript. It consists of A) a short (1-2 sentences) summary of the findings and their significance, B) 3-4 bullet points highlighting key results and C) a synopsis image that is 550x300 pixels large (width x height, jpeg or png format). You can either show a model or key data in the synopsis image. Please note that the image size is rather small and that text needs to be readable at the final size.

Please let me know if you have any questions regarding any of these points. You can use the link below to upload the revised files.

Thank you once more for giving us the chance to consider your manuscript for The EMBO Journal. I look forward to receiving the final version.

With best wishes,

Ieva

Ieva Gailite, PhD
Senior Scientific Editor
The EMBO Journal
Meyerhofstrasse 1
D-69117 Heidelberg
Tel: +4962218891309
i.gailite@embojournal.org

We realize that it is difficult to revise to a specific deadline. In the interest of protecting the conceptual advance provided by the work, we recommend a revision within 3 months (3rd Aug 2026). Please discuss the revision progress ahead of this time with the editor if you require more time to complete the revisions.

Referee #1:

The authors are commended for their rigorous and scholarly engagement with the reviewer comments. They have thoughtfully addressed all of my points. Congratulations on a strong contribution to the literature that you should be proud of.

As a minor note, in the Discussion the authors say that "We hypothesize that the relevant difference for the dependence on sax-1 may be the expression of specific cell-surface proteins. These proteins show differential expression between dauer types, and their endocytosis has been shown to be important for dendrite pruning in other systems." It sounds like the authors are referring to specific candidates ("these proteins show...") and it would be very helpful to explicitly name them.

The authors addressed remaining editorial issues.

Dear Shaul,

I sincerely apologise for the delays in the processing of your manuscript caused by conference travel and the high number of submissions that we currently receive. Thank you for incorporating the final editorial and formatting requests in the manuscript. I am now pleased to inform you that your manuscript has been accepted for publication. Congratulations with a nice study!

Before we forward your manuscript to our publishers, we would like to propose some edits in the manuscript abstract and synopsis. We have also selected a short blurb that will accompany the title of your manuscript in our online table of contents. Please take a look at the proposed textual changes in the attached text file - any corrections or edits from your side are very welcome.

Once I have received your input on these points, I will be happy to forward your manuscript to our publishers for typesetting. Your manuscript will be processed for publication by EMBO Press. It will be copy edited and you will receive page proofs prior to publication. Please note that you will be contacted by Springer Nature Author Services to complete licensing and payment information.

You may qualify for financial assistance for your publication charges - either via a Springer Nature fully open access agreement or an EMBO initiative. Check your eligibility: https://link.springer.com/journal/44318/how-to-publish-with-us

Should you be planning a Press Release on your article, please notify the production team via this form in order to coordinate publication and release dates: https://forms.cloud.microsoft/e/eH4A8JVP6g

I look forward to hearing from you soon regarding the proposed textual changes, If you have any questions, please do not hesitate to contact the Editorial Office or me directly. Thank you for this interesting contribution to The EMBO Journal!

Best wishes,

Ieva

Ieva Gailite, PhD
Senior Scientific Editor
The EMBO Journal
Meyerhofstrasse 1
D-69117 Heidelberg
Tel: +4962218891309
i.gailite@embojournal.org

*****
Please note that it is The EMBO Journal policy for the transcript of the editorial process (containing referee reports and your response letters) to be published as an online supplement to each paper. If you should prefer removal of any referee-only figures included in the point-by-point response(s), e.g. because they may still be used for future publication or because they have been reproduced from published work by others, please do let us know immediately via response email.
More information is available here: https://link.springer.com/partners/embo-press/editorial-policies#Peer%20review
*****

***
Rev_Com_number: RC-2025-03073
New_manu_number: EMBOJ-2025-122251R1
Corr_author: Yogev
Title: NDR kinase SAX-1 controls dendrite branch-specific pruning during neuronal remodeling in C. elegans

EMBO Press Author Checklist

	USEFUL LINKS FOR COMPLETING THIS FORM
Corresponding Author Name: Shaul Yogev	The EMBO Journal - Author Guidelines
Journal Submitted to: EMBO Journal	EMBO Reports - Author Guidelines
Manuscript Number: EMBOJ-2025-122251R	Molecular Systems Biology - Author Guidelines
	EMBO Molecular Medicine - Author Guidelines

Reporting Checklist for Life Science Articles (updated January
This checklist is adapted from Materials Design Analysis Reporting (MDAR) Checklist for Authors. MDAR establishes a minimum set of requirements in transparent reporting in the life sciences (see Statement of Task: 10.31222/osf.io/9sm4x). Please follow the journal's guidelines in preparing your
Please note that a copy of this checklist will be published alongside your article.

Abridged guidelines for figures
1. Data
The data shown in figures should satisfy the following conditions:

- the data were obtained and processed according to the field's best practice and are presented to reflect the results of the experiments in an accurate and unbiased manner.
- ideally, figure panels should include only measurements that are directly comparable to each other and obtained with the same assay.
- plots include clearly labeled error bars for independent experiments and sample sizes. Unless justified, error bars should not be shown for technical
- if n<5, the individual data points from each experiment should be plotted. Any statistical test employed should be justified.
- Source Data should be included to report the data underlying figures according to the guidelines set out in the authorship guidelines on Data

2. Captions
Each figure caption should contain the following information, for each panel where they are relevant:

- a specification of the experimental system investigated (eg cell line, species name).
- the assay(s) and method(s) used to carry out the reported observations and measurements.
- an explicit mention of the biological and chemical entity(ies) that are being measured.
- an explicit mention of the biological and chemical entity(ies) that are altered/varied/perturbed in a controlled manner.
- the exact sample size (n) for each experimental group/condition, given as a number, not a range;
- a description of the sample collection allowing the reader to understand whether the samples represent technical or biological replicates (including how many animals, litters, cultures, etc.).
- a statement of how many times the experiment shown was independently replicated in the laboratory.
- definitions of statistical methods and measures:
  - common tests, such as t-test (please specify whether paired vs. unpaired), simple χ2 tests, Wilcoxon and Mann-Whitney tests, can be unambiguously identified by name only, but more complex techniques should be described in the methods section;
  - are tests one-sided or two-sided?
  - are there adjustments for multiple comparisons?
  - exact statistical test results, e.g., P values = x but not P values < x;
  - definition of 'center values' as median or average;
  - definition of error bars as s.d. or s.e.m.

Please complete ALL of the questions below.
Select "Not Applicable" only when the requested information is not relevant for your study.

Materials

Newly Created Materials	Information included in the manuscript?	In which section is the information available? (Reagents and Tools Table, Materials and Methods, Figures, Data Availability Section)
New materials and reagents need to be available; do any restrictions apply?	Yes	Reagents and tools table - no restrictions apply

Antibodies	Information included in the manuscript?	In which section is the information available? (Reagents and Tools Table, Materials and Methods, Figures, Data Availability Section)
For **antibodies** provide the following information:<br>- Commercial antibodies: RRID (if possible) or supplier name, catalogue number and or/clone number<br>- Non-commercial: RRID or citation	Not Applicable	

DNA and RNA sequences	Information included in the manuscript?	In which section is the information available? (Reagents and Tools Table, Materials and Methods, Figures, Data Availability Section)
**Short novel DNA or RNA including primers, probes**: provide the sequences.	Yes	Reagents and tools table

Cell materials	Information included in the manuscript?	In which section is the information available? (Reagents and Tools Table, Materials and Methods, Figures, Data Availability Section)
**Cell lines:** Provide species information, strain. Provide accession number in repository **OR** supplier name, catalog number, clone number, and/**OR** RRID.	Not Applicable	
**Primary cultures:** Provide species, strain, sex of origin, genetic modification status.	Not Applicable	
Report if the cell lines were recently **authenticated** (e.g., by STR profiling) and tested for mycoplasma contamination.	Not Applicable	

Experimental animals	Information included in the manuscript?	In which section is the information available? (Reagents and Tools Table, Materials and Methods, Figures, Data Availability Section)
**Laboratory animals or Model organisms:** Provide species, strain, sex, age, genetic modification status. Provide accession number in repository **OR** supplier name, catalog number, clone number, **OR** RRID.	Yes	Reagents and tools table
**Animal observed in or captured from the field:** Provide species, sex, and age where possible.	Not Applicable	
Please detail **housing and husbandry conditions**.	Yes	Materials and Methods

Plants and microbes	Information included in the manuscript?	In which section is the information available? (Reagents and Tools Table, Materials and Methods, Figures, Data Availability Section)
**Plants:** provide species and strain, ecotype and cultivar where relevant, unique accession number if available, and source (including location for collected wild specimens).	Not Applicable	
**Microbes:** provide species and strain, unique accession number if available, and source.	Not Applicable	

Human research participants	Information included in the manuscript?	In which section is the information available? (Reagents and Tools Table, Materials and Methods, Figures, Data Availability Section)
If collected and within the bounds of privacy constraints report on age, sex and gender or ethnicity for all study participants.	Not Applicable	

Core facilities	Information included in the manuscript?	In which section is the information available? (Reagents and Tools Table, Materials and Methods, Figures, Data Availability Section)
If your work benefited from core facilities, was their service mentioned in the acknowledgments section?	Yes	Acknowledgements

Design

Study protocol	Information included in the manuscript?	In which section is the information available? (Reagents and Tools Table, Materials and Methods, Figures, Data Availability Section)
If study protocol has been **pre-registered, provide DOI in the manuscript**. For clinical trials, provide the trial registration number **OR** cite DOI.	Not Applicable	
Report the **clinical trial registration number** (at ClinicalTrials.gov or equivalent), where applicable.	Not Applicable	

Laboratory protocol	Information included in the manuscript?	In which section is the information available? (Reagents and Tools Table, Materials and Methods, Figures, Data Availability Section)
Provide DOI OR other citation details if **external detailed step-by-step protocols** are available.	Not Applicable	

Experimental study design and statistics	Information included in the manuscript?	In which section is the information available? (Reagents and Tools Table, Materials and Methods, Figures, Data Availability Section)
Include a statement about **sample size** estimate even if no statistical methods were used.	Yes	Figure legends
Were any steps taken to minimize the effects of subjective bias when allocating animals/samples to treatment (e.g. **randomization procedure**)? If yes, have they been described?	Not Applicable	
Include a statement about **blinding** even if no blinding was done.	Not Applicable	
Describe **inclusion/exclusion criteria** if samples or animals were excluded from the analysis. Were the criteria pre-established?<br>If sample or data points were omitted from analysis, report if this was due to attrition or intentional exclusion and provide justification.	Not Applicable	
For every figure, are **statistical tests** justified as appropriate? Do the data meet the assumptions of the tests (e.g., normal distribution)? Describe any methods used to assess it. Is there an estimate of variation within each group of data? Is the variance similar between the groups that are being statistically compared?	Yes	All samples were tested for normality and appropriate parametric or non-parametric statistical tests were used.

Sample definition and in-laboratory replication	Information included in the manuscript?	In which section is the information available? (Reagents and Tools Table, Materials and Methods, Figures, Data Availability Section)
In the figure legends: state number of times the experiment was **replicated** in laboratory.	Yes	Figure legends report sample size
In the figure legends: define whether data describe **technical or biological replicates**.	Yes	Figure legends report sample size

Ethics

Ethics	Information included in the manuscript?	In which section is the information available? (Reagents and Tools Table, Materials and Methods, Figures, Data Availability Section)
Studies involving **human participants**: State details of **authority granting ethics approval** (IRB or equivalent committee(s), provide reference number for approval.	Not Applicable	
Studies involving **human participants**: Include a statement confirming that **informed consent** was obtained from all subjects and that the experiments conformed to the principles set out in the WMA Declaration of Helsinki and the Department of Health and Human Services Belmont Report.	Not Applicable	
Studies involving **human participants: For publication of patient photos**, include a statement confirming that consent to publish was obtained.	Not Applicable	
Studies involving experimental **animals**: State details of **authority granting ethics approva**l (IRB or equivalent committee(s), provide reference number for approval. Include a statement of compliance with ethical regulations.	Not Applicable	
Studies involving **specimen and field samples:** State if relevant **permits** obtained, provide details of authority approving study; if none were required, explain why.	Not Applicable	

Dual Use Research of Concern (DURC)	Information included in the manuscript?	In which section is the information available? (Reagents and Tools Table, Materials and Methods, Figures, Data Availability Section)
Could your study fall under dual use research restrictions? Please check biosecurity documents and list of **select agents and toxins** (CDC): https://www.selectagents.gov/sat/list.htm	Not Applicable	
If you used a select agent, is the security level of the lab appropriate and reported in the manuscript?	Not Applicable	
If a study is subject to dual use research of concern regulations, is the name of the **authority granting approval and reference number** for the regulatory approval provided in the manuscript?	Not Applicable	

Reporting

The MDAR framework recommends adoption of discipline-specific guidelines, established and endorsed through community initiatives. Journals have their own policy about requiring specific guidelines and recommendations to complement MDAR.

Adherence to community standards	Information included in the manuscript?	In which section is the information available? (Reagents and Tools Table, Materials and Methods, Figures, Data Availability Section)
State if relevant guidelines or checklists (e.g., **ICMJE, MIBBI, ARRIVE, PRISMA**) have been followed or provided.	Not Applicable	
For **tumor marker prognostic studies,** we recommend that you follow the **REMARK** reporting guidelines (see link list at top right). See author guidelines, under 'Reporting Guidelines'. Please confirm you have followed these guidelines.	Not Applicable	
For **phase II and III randomized controlled trials**, please refer to the **CONSORT** flow diagram (see link list at top right) and submit the CONSORT checklist (see link list at top right) with your submission. See author guidelines, under 'Reporting Guidelines'. Please confirm you have submitted this list.	Not Applicable	

Data Availability

Data availability	Information included in the manuscript?	In which section is the information available? (Reagents and Tools Table, Materials and Methods, Figures, Data Availability Section)
Have **primary datasets** been deposited according to the journal's guidelines (see 'Data Deposition' section) and the respective accession numbers provided in the Data Availability Section?	Yes	Primary data is uploaded with other manuscript files
Were **human clinical and genomic datasets** deposited in a public access-controlled repository in accordance to ethical obligations to the patients and to the applicable consent agreement?	Not Applicable	
Are **computational models** that are central and integral to a study available without restrictions in a machine-readable form? Were the relevant accession numbers or links provided?	Not Applicable	
If publicly available data were reused, provide the respective **data citations in the reference list**.	Not Applicable	
